# A Total of Eight Novel Steroidal Glycosides Based on Spirostan, Furostan, Pseudofurostan, and Cholestane from the Leaves of *Cestrum newellii*

**DOI:** 10.3390/molecules25194462

**Published:** 2020-09-28

**Authors:** Tomoki Iguchi, Naoki Takahashi, Yoshihiro Mimaki

**Affiliations:** School of Pharmacy, Tokyo University of Pharmacy and Life Sciences, 1432-1, Horinouchi, Hachioji, Tokyo 192-0392, Japan; y20612@toyaku.ac.jp (N.T.); mimakiy@toyaku.ac.jp (Y.M.)

**Keywords:** *Cestrum newellii*, spirostanol glycoside, furostanol glycoside, pseudofurostanol glycoside, cholestane glycoside, cytotoxic activity, HL-60 cell

## Abstract

Previously, various steroidal glycosides were reported from plants of *Cestrum* species. However, phytochemical investigation has not been conducted on *Cestrum newellii*. A systematic phytochemical investigation of the leaves of *C. newellii* resulted in the isolation of eight novel steroidal glycosides (**1**–**8**), which were classified into three spirostanol glycosides (**1**–**3**), two furostanol glycosides (**4** and **5**), two pseudofurostanol glycosides (**6** and **7**), and one cholestane glycoside (**8**). In addition, three known cholestane glycosides (**9**–**11**) were isolated and identified. The structures of the new compounds were determined based on spectroscopic data and chemical transformations. Compounds **1** and **2** are spirostanol glycosides having hydroxy groups at C-2, C-3, C-12, and C-24 of the aglycone moiety. Although *C. newellii* is known to be a poisonous plant, the 3-(4,5-dimethylthiazol-2-yl)-2,5-diphenyl-2*H*-tetrazolium bromide assay exhibited that none of the isolated compounds were cytotoxic to HL-60 human promyelocytic leukemia cells.

## 1. Introduction

Plants in the genus *Cestrum* (Solanaceae) are native to warm subtropical and tropical areas of America, and are now cultivated all over the world for ornamental purposes [1]. *Cestrum* species are rich sources of steroidal glycosides, and structurally diverse steroidal glycosides have been isolated from *C. laevigatum* [2,3], *C. schlechtendahlii* [4], *C. ruizteranianum* [5], *C. parqui* [6,7,8], *C. diurnum* [9,10], *C. sendtenerianum* [11,12], and *C. nocturnum* [13,14,15]. *C. newellii* is reputed to be a poisonous plant. However, a literature survey suggests that no phytochemical investigation has been done on *C. newellii*. Therefore, a systematic phytochemical analysis of the leaves of *C. newellii* was conducted with a focus on steroidal glycosides. This paper deals with the structural determination of new compounds (**1**–**8**) on the basis of spectroscopic data and chemical transformations. Furthermore, the cytotoxic activities of the isolated compounds (**1**–**11**) were evaluated.

## 2. Results and Discussion

**Figure 1:**
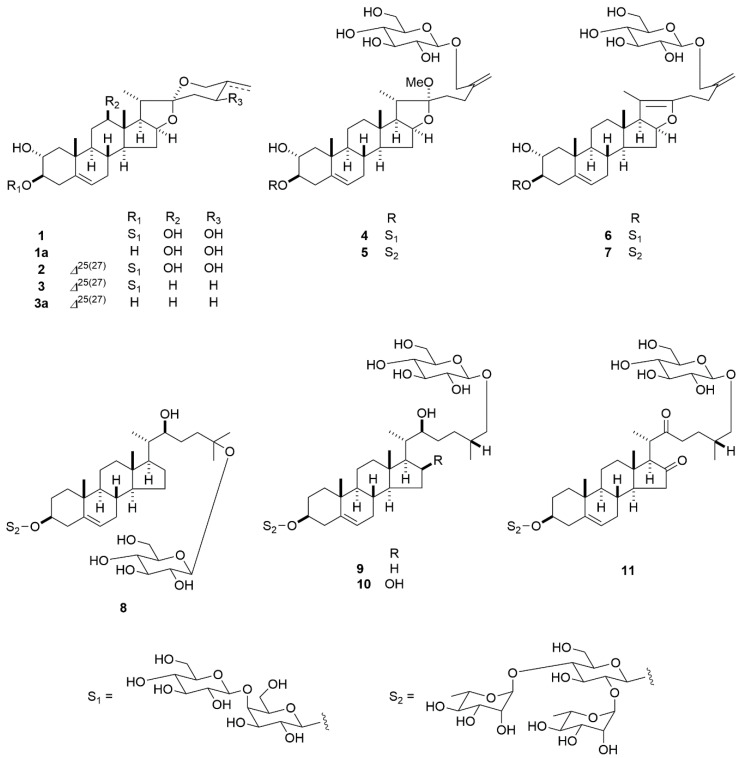
Structures of **1**, **1a**, **2**, **3**, **3a**, **4**–**11**.

The MeOH extract of the leaves of *C. newellii* was fractionated by column chromatography (CC) and preparative HPLC to obtain 11 compounds (**1**-**11**) (Figure 1). Compounds **9**–**11** were identified as (22*S*,25*R*)-26-[(β-d-glucopyranosyl)oxy]-22-hydroxycholest-5-en-3β-yl *O*-α-l-rhamnopyranosyl-(1→2)-*O*-[α-l-rhamnopyranosyl-(1→4)]-β-d-glucopyranoside (**9**) [16], (22*S*,25*R*)-26-[(β-d-glucopyranosyl)oxy]-16β,22-dihydroxycholest-5-en-3β-yl *O*-α-l-rhamnopyranosyl-(1→2)-*O*-[α-l-rhamnopyranosyl-(1→4)]-β-d-glucopyranoside (**10**) [17], and (25*R*)-26-[(β-d-glucopyranosyl)oxy]-3β-[(*O*-α-l-rhamnopyranosyl-(1→2)-*O*-[α-l-rhamnopyranosyl-(1→4)]-β-d-glucopyranosyl)oxy]-cholest-5-ene-16,22-dione (**11**) [18], respectively.

**Figure 2:**
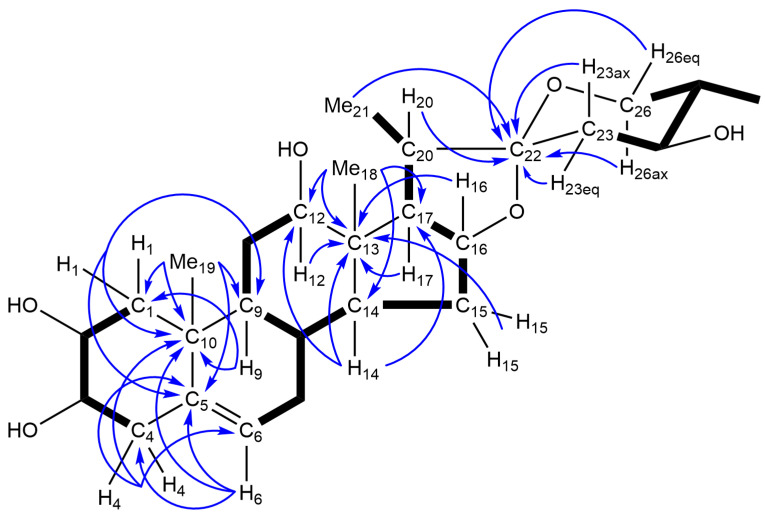
HMBC and ^1^H-^1^H spin-coupling correlations of **1a**. Bold lines indicate the ^1^H-^1^H spin couplings traced by ^1^H-^1^H COSY spectrum and arrows indicate ^1^H/^13^C long-range correlations observed in the HMBC spectrum.

Compound **1** was obtained as an amorphous powder. Its molecular formula was determined to be C_39_H_62_O_16_ based on high-resolution electrospray ionization-time of flight-mass spectroscopy (HRESI-TOF-MS) and ^13^C-NMR data. The infrared (IR) spectrum of **1** exhibited absorption bands for hydroxy groups at 3381 cm^−1^. The ^1^H- and ^13^C-NMR spectra of **1** displayed signals for two tertiary methyl groups at δ_H_ 1.04 (s, Me-18) and 0.94 (s, Me-19); δ_C_ 20.3 (C-19) and 10.9 (C-18), two secondary methyl groups at δ_H_ 1.42 (d, *J* = 6.9 Hz, Me-21) and 1.07 (d, *J* = 6.5 Hz, Me-27); δ_C_ 14.2 (C-21) and 13.6 (C-27), an olefinic group at δ_H_ 5.30 (br d, *J* = 3.6 Hz, H-6); δ_C_ 140.0 (C-5) and 121.9 (C-6), two quaternary carbons at δ_C_ 46.1 (C-13) and 37.9 (C-10), an acetal carbon at δ_C_ 112.0 (C-22), and two anomeric protons and carbons at δ_H_ 5.22 (d, *J* = 7.9 Hz) and 4.91 (d, *J* = 7.8 Hz); δ_C_ 106.9 and 103.2. The above spectroscopic data imply that **1** had a spirost-5-ene diglycoside framework. Enzymatic hydrolysis of **1** with naringinase yielded **1a** (C_27_H_42_O_6_) as the aglycone, and d-glucose and d-galactose as the carbohydrate moieties. Treatment of **1a** with Ac_2_O in pyridine gave tetraacetate (**1b**) of **1a**, indicating that **1a** had four hydroxy groups. In the heteronuclear multiple bond correlation (HMBC) spectrum of **1a**, the angular methyl singlet at δ_H_ 1.09 showed long-range correlations with C-1 at δ_C_ 46.5, C-5 at δ_C_ 141.2, C-9 at δ_C_ 50.1, and C-10 at δ_C_ 38.6, and was assigned to Me-19. The olefinic proton at δ_H_ 5.41 attributed to H-6 exhibited HMBC correlations with C-4 at δ_C_ 40.7, C-5, and C-10. In the heteronuclear multiple quantum coherence (HMQC) spectrum of **1a**, the C-1 and C-4 carbons were correlated to the one-bond coupled protons at δ_H_ 2.39 (dd, *J* = 12.6 and 4.4 Hz, H-1eq) and 1.42 (dd, *J* = 12.6, 12.3 Hz, H-1ax), and 2.69 (H_2_-4), respectively. The H-1eq and H-1ax protons showed spin-coupling correlations with the hydroxymethine proton at δ_H_ 4.12 (ddd, *J* = 12.3, 11.2, 4.4 Hz), whereas the H_2_-4 protons exhibited spin-couplings with the hydroxymethine proton centered at δ_H_ 3.82 (m, *W*_1/2_ = 18.9 Hz) in the ^1^H-^1^H correlation spectroscopy (COSY) spectrum of **1a**. A spin-coupling correlation was observed between the two hydroxymethine protons (H-2 and H-3) with a *J* value of 11.2 Hz. These data are consistent with the presence of a hydroxy group at C-2 and C-3. Another angular methyl singlet at δ_H_ 1.08 assigned to Me-18 showed HMBC correlations with C-12 at δ_C_ 78.9, C-13 at δ_C_ 46.2, C-14 at δ_C_ 55.4, and C-17 at δ_C_ 62.3. The C-12 carbon was associated with the one-bond coupled proton at δ_H_ 3.58 (dd, *J* = 11.2 and 4.4 Hz) in the HMQC spectrum, from which spin-coupling correlations were observed for the H_2_-11 methylene protons at δ_H_ 2.03 (m, H-11eq) and 1.75 (q-like, *J* = 11.2 Hz, H-11ax). Thus, a hydroxy group was shown to be present at C-12. The methine proton at δ_H_ 1.85 (m) assignable to H-25 displayed spin-coupling correlations with the Me-27 protons at δ_H_ 1.10 (d, *J* = 6.7 Hz), H_2_-26 methylene protons at δ_H_ 3.72 (dd, *J* = 11.2, 4.9 Hz, H-26eq) and 3.63 (dd, *J* = 12.3, 11.2 Hz, H-26ax), and H-24 methine proton at δ_H_ 4.05 (ddd, *J* = 10.5, 10.5, 4.8 Hz). These correlations are indicative of the presence of a hydroxy group at C-24 (Figure 2).

**Figure 3:**
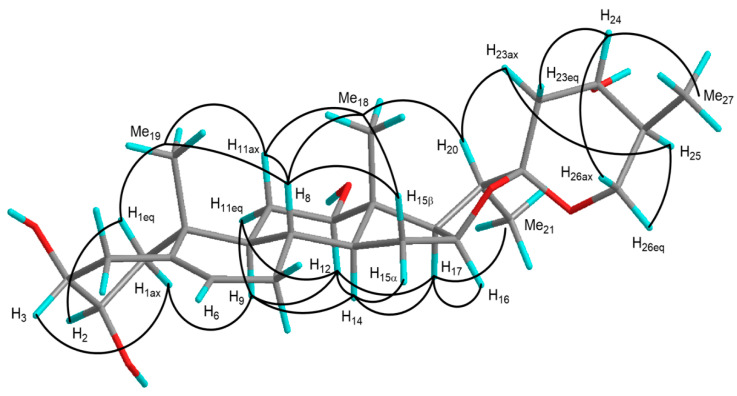
NOE correlations of **1a**.

Accordingly, the planar structure of **1a** was identified as spirost-5-ene-2,3,12,24-tetrol. NOE correlations in the nuclear Overhauser enhancement spectroscopy (NOESY) and proton spin-coupling constants allowed the stereochemistry of **1a** to be determined. The B/C-*trans*, C/D-*trans*, and D/E-*cis* ring junctions, and the configurations of 20α and 22α were confirmed by the following NOE correlations: between H-8 and H-11ax/H-15α/Me-18/Me-19, H-9 and H-11eq/H-12/H-14, H-14 and H-12/H-15α/H-17, H-17 and H-16/Me-21, Me-18 and H-20, and between H-20 and H-23ax (Figure 3). The configurations of the C-2, C-3, and C-12 hydroxy groups were assigned as 2α, 3β, and 12β, respectively, based on the proton spin-coupling constants, ^3^*J*_H-1ax,H-2_ = 12.3 Hz, ^3^*J*_H-1eq,H-2_ = 4.4 Hz, ^3^*J*_H-11ax,H-12_ = 11.2 Hz, and ^3^*J*_H-11eq,H-12_ = 4.4 Hz, and NOE correlations were observed between H-1eq and H-2/Me-19, H-1ax and H-3/H-9, and between H-12 and H-9/H-11eq/H-14/H-17 (Figure 3). The proton spin-coupling constants, ^3^*J*_H-23ax,H-24_ = 10.5 Hz, ^3^*J*_H-23eq,H-24_ = 4.8 Hz, ^3^*J*_H-24,H-25_ = 10.5 Hz, ^3^*J*_H-25,H-26ax_ = 12.3 Hz, and ^3^*J*_H-25,H-26eq_ = 4.9 Hz, and NOE correlations between H-25 and H-23ax/H-26eq, and between H-24 and H-23eq/H-26ax/Me-27 were consistent with the 24*S* and 25*S* configurations (Figure 3). Thus, **1a** was identified as (24*S*,25*S*)-spirost-5-ene-2α,3β,12β,24-tetrol. The ^1^H-^1^H COSY and HMQC spectra of **1** suggest that the sugar moiety of **1** comprised a 4-substituted β-d-galactopyranosyl unit [Gal: δ_H_ 4.91 (1H, d, *J* = 7.8 Hz); δ_C_ 103.2, 73.0, 75.0, 79.9, 75.8, and 60.9 (C-1′–6′)] and a terminal β-d-glucopyranosyl unit [Glc: δ_H_ 5.22 (1H, d, *J* = 7.9 Hz); δ_C_ 106.9, 75.7, 78.5, 72.0, 78.3, and 62.8 (C-1′′–6′′)]. In the HMBC spectrum of **1**, long-range correlations were observed between H-1′′ of Glc (δ_H_ 5.22) and C-4′ of Gal (δ_C_ 79.9), and between H-1′ of Gal (δ_H_ 4.91) and C-3 of the aglycone (δ_C_ 84.5). Based on the above data, **1** was identified as (24*S*,25*S*)-2α,12β,24-trihydroxyspirost-5-en-3β-yl *O*-β-d-glucopyranosyl-(1→4)-β-d-galactopyranoside.

The ^1^H- and ^13^C-NMR spectral data of **2** (C_39_H_60_O_16_) suggest that **2** is analogous to **1**, including the diglycoside moiety attached to C-3 of the aglycone. However, the molecular formula of **2** was smaller than that of **1** by two hydrogen atoms. When the ^1^H- and ^13^C-NMR spectra of **2** were compared with those of **1**, the Me-27 group was revealed to be displaced by an exomethylene group [δ_H_ 5.66 and 5.10 (each br s, H_2_-27); δ_C_ 106.4 (C-27) and 149.3 (C-25)] in **2**. Thus, it was speculated that **2** corresponded to the C-25/27 dehydroxy derivative of **1**. This was supported by HMBC correlations from H_2_-27 (δ_H_ 5.66 and 5.10) to C-24 (δ_C_ 67.0)/C-25 (δ_C_ 149.3)/C-26 (δ_C_ 64.6). Accordingly, **2** was identified as (24*S*)-2α,12β,24-trihydroxyspirosta-5,25(27)-dien-3β-yl *O*-β-d-glucopyranosyl-(1→4)-β-d-galactopyranoside.

Compound **3** (C_39_H_60_O_14_) was obtained as an amorphous solid. The ^1^H- and ^13^C-NMR spectral data of **3** were similar to those of **2**, including the signals of the diglycoside unit bound to C-3 of the aglycone of **3**. However, the molecular formula of **3** was found to be smaller than that of **2** by two oxygen atoms, suggesting that the aglycone of **3** had two less hydroxy groups than **2**. Acid hydrolysis of **3** with 1 M HCl (dioxane/H_2_O, 1:1) gave aglycone (**3a**), d-galactose, and d-glucose. The ^1^H- and ^13^C-NMR spectra of **3a** showed signals for two angular methyl groups at δ_H_ 1.07 (s, Me-19) and 0.82 (s, Me-18); δ_C_ 20.6 (C-19) and 16.3 (C-18), a secondary methyl group at δ_H_ 1.08 (d, *J* = 6.4 Hz, Me-21); δ_C_ 14.9 (C-21), an exomethylene group at δ_H_ 4.81 and 4.77 (each br s, H_2_-27); δ_C_ 108.7 (C-27) and 144.4 (C-25), an acetal carbon at δ_C_ 109.4 (C-22), an olefinic group at δ_H_ 5.40 (br d, *J* = 4.4 Hz, H-6); δ_C_ 141.2 (C-5) and 121.2 (C-6), and two vicinal hydroxy groups at δ_H_ 4.16 (ddd, *J* = 12.0, 11.2, 4.2 Hz, H-2) and 3.84 (m, *W*_1/2_ = 20.1 Hz, H-3); δ_C_ 72.6 (C-2) and 76.7 (C-3). These spectroscopic data imply that **3a** was spirosta-5,25(27)-diene-2α,3β-diol. The HMBC spectrum provided evidence that the *O*-β-d-glucopyranosyl-(1→4)-β-d-galactopyranosyl group was present at C-3 of the aglycone in **3**. Therefore, **3** was deduced to be 2α-hydroxyspirosta-5,25(27)-dien-3β-yl *O*-β-d-glucopyranosyl-(1→4)-β-d-galactopyranoside.

Compound **4** was obtained as an amorphous solid, and its molecular formula was determined to be C_46_H_74_O_20_ based on HRESI-TOF-MS and ^13^C-NMR data. In the ^1^H- and ^13^C-NMR spectra of **4**, the following signals were observed: three steroidal methyl groups at δ_H_ 1.14 (d, *J* = 6.9 Hz, Me-21), 0.93 (s, Me-19), and 0.76 (s, Me-18); δ_C_ 20.3 (C-19), 16.1 (C-21), and 16.0 (C-18), an exomethylene group at δ_H_ 5.34 and 5.04 (each br s, H_2_-27); δ_C_ 146.7 (C-25) and 111.0 (C-27), an olefinic group at δ_H_ 5.30 (br d, *J* = 4.6 Hz, H-6); δ_C_ 140.0 (C-5) and 121.8 (C-6), and three anomeric protons and carbons at δ_H_ 5.23 (d, *J* = 7.9 Hz, H-1′′), 4.93 (d, *J* = 7.8 Hz, H-1′), and 4.90 (d, *J* = 7.8 Hz, H-1′′′); δ_C_ 106.9 (C-1′′), 103.7 (C-1′′′), and 103.3 (C-1′). In addition, an acetal carbon signal at δ_C_ 112.3, a methoxy proton and carbon signals at δ_H_ 3.23 (s); δ_C_ 47.3, and a positive color reaction in Ehrlich’s test suggested that **4** was a 22-methoxyfurostanol glycoside. Compound **4** was treated with β-d-glucosidase to obtain the corresponding spirostanol glycoside (**3**) and d-glucose. A ^3^*J*_C,H_ correlation from H-1′′′ of β-d-glucopyranosyl (δ_H_ 4.90) to C-26 of the aglycone (δ_C_ 71.9) was observed in the HMBC spectrum of **4**. The C-22α configuration was confirmed by the NOE correlation observed between -OMe (δ_H_ 3.23) and H-16 (δ_H_ 4.41) of the aglycone. Thus, **4** was determined to be 26-[(β-d-glucopyranosyl)oxy]-2α-hydroxy-22α-methoxyfurosta-5,25(27)-dien-3β-yl *O*-β-d-glucopyranosyl-(1→4)-β-d-galactopyranoside.

The ^1^H- and ^13^C-NMR spectroscopic features of **5** (C_52_H_84_O_23_) were similar to those of **4**, except for the signals assignable to the sugar moiety attached to C-3 of the aglycone. The molecular formula of **5** was larger than that of **4** by C_6_H_10_O_3_, corresponding to a hexosyl unit. Acid hydrolytic cleavage of **5** with 1 M HCl (dioxane/H_2_O, 1:1) afforded **3a**, d-glucose, and l-rhamnose. Analysis of the ^1^H-^1^H COSY and HMQC spectra for the sugar moieties of **5** indicated the presence of a 2,4-disubstituted β- d-glucopyranosyl unit [Glc (I): δ_H_ 4.96 (1H, d, *J* = 7.2 Hz, H-1′); δ_C_ 100.9, 77.6, 77.6, 78.5, 76.9, and 61.0 (C-1′–6′)], a terminal β-d-glucopyranosyl unit [Glc (II): δ_H_ 4.90 (1H, d, *J* = 7.8 Hz, H-1′’’’); δ_C_ 103.7, 75.0, 78.5, 71.6, 78.4, and 62.7 (C-1′′′′–6′′′′)], and two terminal α-l-rhamnopyranosyl units [Rha (I): δ_H_ 6.33 (1H, br s, H-1′’); δ_C_ 101.9, 72.2, 72.7, 73.9, 69.4, and 18.5 (C-1′′–6′′); Rha (II): δ_H_ 5.79 (1H, br s, H-1′′′); δ_C_ 102.7, 72.3, 72.6, 73.8, 70.3, and 18.4 (C-1′′′–6′′′)]. In the HMBC spectrum of **5**, long-range correlations were observed between H-1′′ of Rha (I) (δ_H_ 6.33) and C-2′ of Glc (I) (δ_C_ 77.6), H-1′′′ of Rha (II) (δ_H_ 5.79) and C-4′ of Glc (I) (δ_C_ 78.5), H-1′ of Glc (I) (δ_H_ 4.96) and C-3 of the aglycone (δ_C_ 84.9), and between H-1′′′′ of Glc (II) (δ_H_ 4.90) and C-26 of the aglycone (δ_C_ 71.9). Therefore, **5** was characterized as 26-[(β-d-glucopyranosyl)oxy]-2α-hydroxy-22α-methoxyfurosta-5,25(27)-dien-3β-yl *O*-α-l-rhamnopyranosyl-(1→2)-*O*-[α-l-rhamnopyranosyl-(1→4)]-β-d-glucopyranoside.

**Figure 4:**
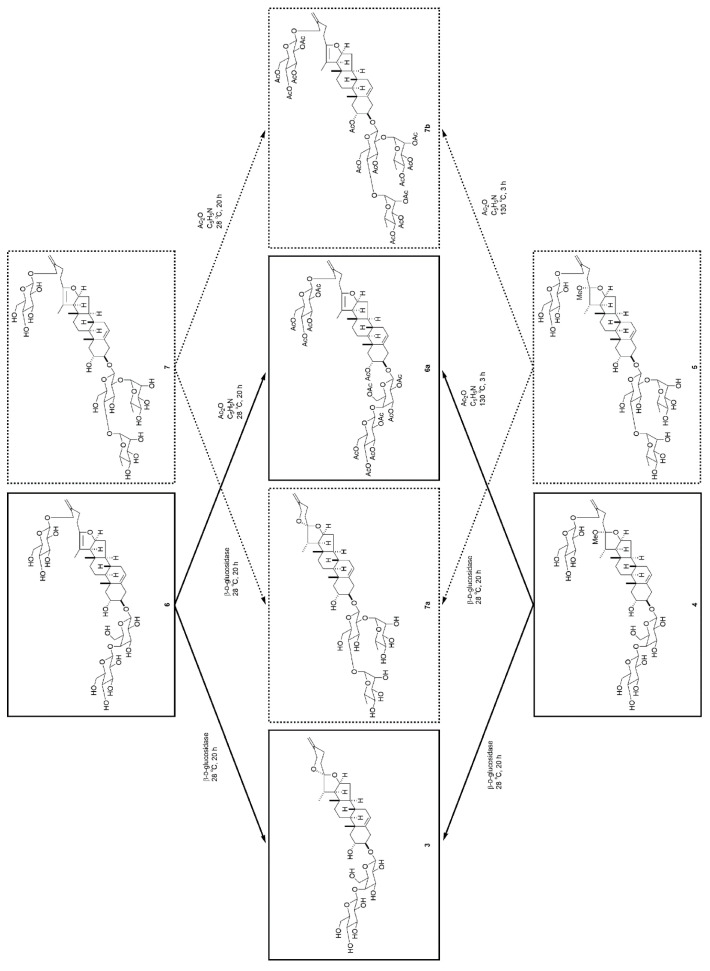
Chemical transformations of **4**–**7**.

The ^1^H- and ^13^C-NMR spectra of **6** (C_45_H_70_O_19_) and **7** (C_51_H_80_O_22_) were closely related to those of **4** and **5**, respectively, except for the signals attributable to the E-ring part of the aglycone. Instead of the secondary methyl signal for Me-21 [**4**: δ_H_ 1.14 (d, *J* = 6.9 Hz); δ_C_ 16.1; **5**: δ_H_ 1.14 (d, *J* = 6.9 Hz); δ_C_ 16.1] and a methoxy signal [**4**: δ_H_ 3.23 (s); δ_C_ 47.3; **5**: δ_H_ 3.24 (s); δ_C_ 47.3], the signals arising from the tertiary methyl groups [**6**: δ_H_ 1.60 (s); δ_C_ 11.7; **7**: δ_H_ 1.59 (s); δ_C_ 11.7] and tetrasubstituted olefinic carbons [**6**: δ_C_ 151.6 and 103.9; **7**: δ_C_ 151.6 and 103.9] were observed in the ^1^H- and ^13^C-NMR spectra of **6** and **7**. Thus, **6** and **7** were thought to be the corresponding pseudofurostanol glycosides of **4** and **5**, respectively. The structures of **6** and **7** were confirmed by the following chemical transformations (Figure 4). Enzymatic hydrolysis of **6** with β-d-glucosidase gave **3** and d-glucose. Furthermore, complete acetylation of **6** with Ac_2_O in pyridine afforded dodecaacetate (**6a**), which agreed with the peracetate of the dehydro derivative of **4** prepared by treating **4** with Ac_2_O in pyridine at 130 °C for 3 h. On the other hand, enzymatic hydrolysis of **7** with β-d-glucosidase gave the spirostanol glycoside (**7a**), which is in agreement with the glycoside obtained by enzymatic hydrolysis **5**. Tridecaacetate (**7b**) of **7** was consistent with the product prepared from **5** upon treatment of **5** with Ac_2_O in pyridine at 130 °C for 3 h. Accordingly, **6** and **7** were identified as 26-[(β-d-glucopyranosyl)oxy]-2α-hydroxyfurosta-5,20(22),25(27)-trien-3β-yl *O*-β-d-glucopyranosyl-(1→4)-β-d-galactopyranoside and 26-[(β-d-glucopyranosyl)oxy]-2α-hydroxyfurosta-5,20(22),25(27)-trien-3β-yl *O*-α-l-rhamnopyranosyl-(1→2)-*O*-[α-l-rhamnopyranosyl-(1→4)]-β-d-glucopyranoside, respectively.

Compound **8** (C_51_H_86_O_21_) was obtained as an amorphous solid. The molecular formula of **8** was the same as that of **9**, and the ^1^H- and ^13^C-NMR spectra of **8** were very similar to those of **9**, except for the signals attributable to the side chain of the aglycone units. Compound **8** was enzymatically hydrolyzed with naringinase, yielding (22*S*)-cholest-5-ene-3β,22,25-triol (**8a**) [19], d-glucose, and l -rhamnose. The ^1^H- and ^13^C-NMR spectra of **8** imply the presence of a 2,4-disubstituted β-d-glucopyranosyl unit [Glc (I)], two terminal α-l-rhamnopyranosyl units [Rha (I) and Rha (II)], and a terminal β-d-glucopyranosyl unit [Glc (II): δ_H_ 5.08 (1H, d, *J* = 7.8 Hz, H-1′′′′); δ_C_ 98.6, 75.4, 78.8, 71.8, 78.0, and 62.9 (C-1′′′′–6′′′′)] in this molecule. In the HMBC spectrum of **8**, a ^3^*J*_C,H_ correlation was observed between H-1′′′′ of Glc (II) (δ_H_ 5.08) and C-25 of the aglycone moiety (δ_C_ 77.4). Therefore, **8** was determined to be (22*S*)-25-[(β-d-glucopyranosyl)oxy]-22-hydroxycholest-5-en-3β-yl *O*-α-l-rhamnopyranosyl-(1→2)-*O*-[α-l-rhamnopyranosyl-(1→4)]-β-d-glucopyranoside.

The isolated compounds (**1**–**11**) were evaluated for their cytotoxic activity toward HL-60 human promyelocytic leukemia cells using a modified 3-(4,5-dimethylthiazol-2-yl)-2,5-diphenyl-2*H*-tetrazolium bromide (MTT) assay method. None of **1**–**11** exhibited cytotoxicity at a sample concentration of up to 10 μM. Although *C. newellii* is regarded to be a poisonous plant, the MTT assay exerted that none of the isolated compounds were cytotoxic against HL-60 cells.

## 3. Materials and Methods

### 3.1. General

Optical rotations were measured on a JASCO P-1030 and a JASCO DIP-360 (JASCO, Tokyo, Japan) automatic digital polarimeter. IR spectra were obtained using a FT/IR-620 (JASCO) spectrophotometer. NMR spectral data were recorded on a DRX-500 (500 MHz for ^1^H-NMR, 125 MHz for ^13^C-NMR) spectrometer using standard Bruker pulse programs at 300 K (Bruker, Karlsruhe, Germany). Chemical shifts are given as δ values with reference to tetramethylsilane (TMS) as an internal standard. HRESI-TOF-MS data were obtained using a Waters Micromass LCT mass spectrometer (Waters, MA, USA). Diaion HP-20 porous polymer polystyrene resin (Mitsubishi-Chemical, Tokyo, Japan), silica gel Chromatrex BW-300 (Fuji-Silysia Chemical, Aichi, Japan), and ODS silica gel COSMOSIL 75C_18_-OPN (Nacalai Tesque, Kyoto, Japan) were used for CC. Thin-layer chromatography (TLC) analysis was conducted using precoated silica gel 60F_254_ or RP_18_ F_254_S plates (0.25 mm thick; Merck, Darmstadt, Germany), and the spots were made visible by spraying the plates with H_2_SO_4_/H_2_O (1:9), followed by heating. TLC was used to check the progress of the separation of fractions and to confirm the purity of the isolated compounds. A Tosoh CCPM (Tosoh, Tokyo, Japan) or a Tosoh-8020 (Tosoh), Tosoh RI-8020 (Tosoh), or Shodex OR-2 (Showa-Denko, Tokyo, Japan) detector, and a Rheodyne injection port (Rohnert Park, CA, USA) constituted the HPLC system. A Capcell Pak C_18_ UG120 column (10 mm i.d. × 250 mm, 5 μm, Shiseido, Tokyo, Japan) was used for preparative HPLC. Enzymatic hydrolysis was carried out using β-d-glucosidase (EC 232-589-7; Sigma, St. Louis, MO, USA) or naringinase (EC 232-962-4; Sigma, St. Louis, MO, USA).

### 3.2. Plant Material

The leaves of *C. newellii* were purchased from Sakata Seed Corporation (Kanagawa, Japan) and grown in the medicinal botanical garden of Tokyo University of Pharmacy and Life Sciences (TUPLS). A voucher specimen was kept at the herbarium of the TUPLS.

### 3.3. Extraction and Isolation

*C. newellii* leaves (dry weight, 3.6 kg) were extracted with MeOH at 60 °C for 2 h, and concentrated under reduced pressure to obtain the MeOH extract (180 g). Then, all MeOH extract was loaded onto a Diaion HP-20 column, and successively eluted with MeOH/H_2_O (3:7), EtOH, and EtOAc (each 12 L). The EtOH eluted fraction was separated by silica gel CC and eluted with a stepwise gradient mixture of CHCl_3_/MeOH (9:1, 6:1, 3:1, 1:1) to obtain three fractions (Frs. I–III). Fraction II was further divided by ODS silica gel CC eluted with MeCN/H_2_O (1:3, 1:1, 3:1, 5:1) to yield seven subfractions (Frs. II-1–II-7). Fraction II-1 was purified by silica gel CC eluted with CHCl_3_/MeOH/H_2_O (20:10:1), ODS silica gel CC eluted with MeCN/H_2_O (1:3), and preparative HPLC using MeCN/H_2_O (5:11) to obtain **6** (21 mg) and **7** (12 mg). Fraction II-2 was subjected to silica gel CC eluted with CHCl_3_/MeOH/H_2_O (20:10:1; 7:4:1), ODS silica gel CC eluted with MeCN/H_2_O (2:7), and preparative HPLC using MeCN/H_2_O (1:3) to obtain **4** (1.8 g). Fraction II-3 was separated by silica gel CC eluted with CHCl_3_/MeOH/H_2_O (25:10:1; 20:10:1), ODS silica gel CC eluted with MeCN/H_2_O (5:13; 5:14), and preparative HPLC using MeCN/H_2_O (10:27) to yield **1** (49 mg) and **2** (8.3 mg). Fraction II-5 was applied to silica gel CC eluted with CHCl_3_/MeOH/H_2_O (30:10:1; 25:10:1; 20:10:1; 7:4:1) and ODS silica gel CC eluted with MeCN/H_2_O (1:1; 1:2; 5:12; 2:5; 10:27; 1:3), and preparative HPLC using MeCN/H_2_O (5:12) to afford **5** (22 mg), **8** (29 mg), **9** (20 mg), **10** (65 mg), and **11** (71 mg). Fraction II-7 was chromatographed on silica gel and ODS silica gel eluted with CHCl_3_/MeOH/H_2_O (30:10:1) and MeCN/H_2_O (1:1), respectively, to furnish **3** (25 mg).

### 3.4. Structural Characterization

Compound **1**: Amorphous solid; [α]_D_^25^ − 62.5 (*c* = 0.10, MeOH); IR (film) ν_max_: 3381 (OH), 2907 (CH) cm^−1^; HRESI-TOF-MS *m/z*: 787.4146 [M + H]^+^ (calcd. for C_39_H_63_O_16_: 787.4116). ^1^H-NMR spectral data (500 MHz, C_5_D_5_N): δ_H_ 5.30 (1H, br d, *J* = 3.6 Hz, H-6), 5.22 (1H, d, *J* = 7.9 Hz, H-1′′), 4.91 (1H, d, *J* = 7.8 Hz, H-1′), 4.63 (1H, br d, *J* = 3.5 Hz, H-4′), 4.61 (1H, q-like, *J* = 8.4 Hz, H-16), 4.56 (1H, dd, *J* = 10.9, 8.0 Hz, H-6′a), 4.53 (1H, dd, *J* = 11.2, 2.4 Hz, H-6′′a), 4.41 (1H, dd, *J* = 9.3, 7.8 Hz, H-2′), 4.25 (1H, dd, *J* = 9.3, 3.5 Hz, H-3′), 4.23 (1H, dd, *J* = 9.5, 8.6 Hz, H-3′’), 4.22 (1H, dd, *J* = 10.9, 4.2 Hz, H-6′b), 4.19 (1H, dd, *J* = 11.2, 4.7 Hz, H-6′′b), 4.09 (1H, m, H-5′), 4.08 (1H, dd, *J* = 8.6, 7.9 Hz, H-2′′), 4.07 (1H, dd, *J* = 9.5, 8.8 Hz, H-4′′), 4.03 (1H, m, H-24), 4.01 (1H, m, H-2), 3.96 (1H, ddd, *J* = 8.8, 4.7, 2.4 Hz, H-5′′), 3.79 (1H, m, H-3), 3.70 (1H, dd, *J* = 11.2, 4.8 Hz, H-26eq), 3.60 (1H, dd, *J* = 12.2, 11.2 Hz, H-26ax), 3.55 (1H, dd, *J* = 11.0, 4.2 Hz, H-12), 1.42 (3H, d, *J* = 6.9 Hz, Me-21), 1.07 (3H, d, *J* = 6.5 Hz, Me-27), 1.04 (3H, s, Me-18), 0.94 (3H, s, Me-19). For ^13^C-NMR spectral data, see Table 1. For NMR spectral data, see Supplementary Materials.

Compound **1a**: Amorphous solid; [α]_D_^25^ − 44.2 (*c* = 0.10, MeOH); IR (film) ν_max_: 3364 (OH), 2924 and 2872 (CH) cm^−1^; HRESI-TOF-MS *m/z*: 463.3084 [M + H]^+^ (calcd. for C_27_H_43_O_6_: 463.3060). ^1^H-NMR spectral data (500 MHz, C_5_D_5_N): δ_H_ 5.41 (1H, br d, *J* = 4.8 Hz, H-6), 4.63 (1H, q-like, *J* = 7.5 Hz, H-16), 4.12 (1H, ddd, *J* = 12.3, 11.2, 4.4 Hz, H-2), 4.05 (1H, ddd, *J* = 10.5, 10.5, 4.8 Hz, H-24), 3.82 (1H, m, *W*_1/2_ = 18.9 Hz, H-3), 3.72 (1H, dd, *J* = 11.2, 4.9 Hz, H-26eq), 3.63 (1H, dd, *J* = 12.3, 11.2 Hz, H-26ax), 3.58 (1H, dd, *J* = 11.2, 4.4 Hz, H-12), 1.45 (3H, d, *J* = 6.9 Hz, Me-21), 1.10 (3H, d, *J* = 6.7 Hz, Me-27), 1.09 (3H, s, Me-19), 1.08 (3H, s, Me-18). For ^13^C-NMR spectral data, see Table 1. For NMR spectral data, see Supplementary Materials.

Tetraacetate of compound **1a** (**1b**): ^1^H-NMR spectral data (500 MHz, C_5_D_5_N): δ_H_ 5.34 (1H, br d, *J* = 4.8 Hz, H-6), 5.04 (1H, m, H-3), 4.84 (1H, dd, *J* = 11.2, 4.5 Hz, H-12), 1.16 (3H, d, *J* = 6.8 Hz, Me-21), 1.06 (3H, s, Me-18), 0.92 (3H, s, Me-19), 0.80 (3H, d, *J* = 6.5 Hz, Me-27), 2.20, 2.10, 2.09 × 2 (each 3H, Ac × 4).

Compound **2**: Amorphous solid; [α]_D_^25^ − 78.0 (*c* = 0.10, MeOH); IR (film) ν_max_: 3381 (OH), 2921 (CH) cm^−1^; HRESI-TOF-MS *m*/*z*: 785.3943 [M + H]^+^ (calcd. for C_39_H_61_O_16_: 785.3960). ^1^H-NMR spectral data (500 MHz, C_5_D_5_N): δ_H_ 5.66 (1H, br s, H-27a), 5.31 (1H, br d, *J* = 4.8 Hz, H-6), 5.24 (1H, d, *J* = 7.9 Hz, H-1′′), 5.10 (1H, br s, H-27b), 5.06 (1H, dd, *J* = 11.3, 6.2 Hz, H-24), 4.92 (1H, d, *J* = 7.7 Hz, H-1′), 4.60 (1H, q-like, *J* = 6.9 Hz, H-16), 4.53 (1H, d, *J* = 12.8 Hz, H-26eq), 4.23 (1H, d, *J* = 12.8 Hz, H-26ax), 3.98 (1H, m, H-2), 3.80 (1H, m, H-3), 3.55 (1H, dd, *J* = 11.0, 4.4 Hz, H-12), 1.37 (3H, d, *J* = 6.9 Hz, Me-21), 1.05 (3H, s, Me-18), 0.95 (3H, s, Me-19). For ^13^C-NMR spectral data, see Table 1. For NMR spectral data, see Supplementary Materials.

Compound **3**: Amorphous solid; [α]_D_^25^ − 54.6 (*c* = 0.10, MeOH); IR (film) ν_max_: 3369 (OH), 2927 and 2852 (CH) cm^−1^; HRESI-TOF-MS *m*/*z*: 753.4058 [M + H]^+^ (calcd. for C_39_H_61_O_14_: 753.4061). ^1^H-NMR spectral data (500 MHz, C_5_D_5_N): δ_H_ 5.30 (1H, br d, *J* = 4.7 Hz, H-6), 5.23 (1H, d, *J* = 7.9 Hz, H-1′′), 4.93 (1H, d, *J* = 7.8 Hz, H-1′), 4.80 (1H, br s, H-27a), 4.77 (1H, br s, H-27b), 4.51 (1H, q-like, *J* = 7.9 Hz, H-16), 4.43 (1H, d, *J* = 12.4 Hz, H-26ax), 4.05 (1H, m, H-2), 4.02 (1H, d, *J* = 12.4 Hz, H-26eq), 3.82 (1H, m, H-3), 1.06 (3H, d, *J* = 7.0 Hz, Me-21), 0.94 (3H, s, Me-19), 0.79 (3H, s, Me-18). For ^13^C-NMR spectral data, see Table 1. For NMR spectral data, see Supplementary Materials.

Compound **3a**: Amorphous solid; [α]_D_^25^ − 78.6 (*c* = 0.10, MeOH); IR (film) ν_max_: 3351 (OH), 2926 and 2852 (CH) cm^−1^; HRESI-TOF-MS *m*/*z*: 429.3010 [M + H]^+^ (calcd. for C_27_H_41_O_4_: 429.3005). ^1^H-NMR spectral data (500 MHz, C_5_D_5_N): δ_H_ 5.40 (1H, br d, *J* = 4.4 Hz, H-6), 4.81 (1H, br s, H-27a), 4.77 (1H, br s, H-27b), 4.52 (1H, q-like, *J* = 7.1 Hz, H-16), 4.44 (1H, d, *J* = 12.1 Hz, H-26ax), 4.16 (1H, ddd, *J* = 12.0, 11.2, 4.2 Hz, H-2), 4.02 (1H, d, *J* = 12.1 Hz, H-26eq), 3.84 (1H, m, *W*_1/2_ = 20.1 Hz, H-3), 1.08 (3H, d, *J* = 6.4 Hz, Me-21), 1.07 (3H, s, Me-19), 0.82 (3H, s, Me-18). For ^13^C-NMR spectral data, see Table 1. For NMR spectral data, see Supplementary Materials.

Compound **4**: Amorphous solid; [α]_D_^25^ − 53.4 (*c* = 0.10, MeOH); IR (film) ν_max_: 3369 (OH), 2936 and 2899 (CH) cm^−1^; HRESI-TOF-MS *m*/*z*: 915.4611 [M + H − MeOH]^+^ (calcd. for C_45_H_71_O_19_: 915.4590). ^1^H-NMR spectral data (500 MHz, C_5_D_5_N): δ_H_ 5.34 (1H, br s, H-27a), 5.30 (1H, br d, *J* = 4.6 Hz, H-6), 5.23 (1H, d, *J* = 7.9 Hz, H-1′′), 5.04 (1H, br s, H-27b), 4.93 (1H, d, *J* = 7.8 Hz, H-1′), 4.90 (1H, d, *J* = 7.8 Hz, H-1′′′), 4.61 (1H, d, *J* = 12.8 Hz, H-26a), 4.53 (1H, dd, *J* = 11.9, 2.4 Hz, H-6′′′a), 4.41 (1H, m, H-16), 4.36 (1H, dd, *J* = 11.9, 5.6 Hz, H-6′′′b), 4.35 (1H, d, *J* = 12.8 Hz, H-26b), 4.26 (1H, dd, *J* = 8.7, 8.7 Hz, H-3′′′), 4.21 (1H, dd, *J* = 8.7, 8.7 Hz, H-4′′′), 4.06 (1H, dd, *J* = 8.7, 7.8 Hz, H-2′′′), 4.04 (1H, m, H-2), 3.94 (1H, m, H-5′′′), 3.81 (1H, m, H-3), 3.23 (3H, s, OMe), 1.14 (3H, d, *J* = 6.9 Hz, Me-21), 0.93 (3H, s, Me-19), 0.76 (3H, s, Me-18). For ^13^C-NMR spectral data, see Table 1. For NMR spectral data, see Supplementary Materials.

Compound **5**: Amorphous solid; [α]_D_^25^ − 52.0 (*c* = 0.10, MeOH); IR (film) ν_max_: 3382 (OH), 2924 (CH) cm^−1^; HRESI-TOF-MS *m*/*z*: 1045.5264 [M + H − MeOH]^+^ (calcd. for C_51_H_81_O_22_: 1045.5220). ^1^H-NMR spectral data (500 MHz, C_5_D_5_N): δ_H_ 6.33 (1H, br s, H-1′′), 5.79 (1H, br s, H-1′′′), 5.34 (1H, br s, H-27a), 5.32 (1H, br d, *J* = 4.4 Hz, H-6), 5.05 (1H, br s, H-27b), 4.96 (1H, d, *J* = 7.2 Hz, H-1′), 4.90 (1H, d, *J* = 7.8 Hz, H-1′′′′), 4.86 (1H, m, H-5′′), 4.85 (1H, m, H-5′′′), 4.81 (1H, br s, H-2′′), 4.65 (1H, br s, H-2′′′), 4.62 (1H, d, *J* = 12.7 Hz, H-26a), 4.61 (1H, dd, *J* = 9.0, 3.7 Hz, H-3′′), 4.53 (1H, dd, *J* = 12.5, 3.4 Hz, H-6′′′′a), 4.52 (1H, dd, *J* = 9.3, 3.4 Hz, H-3′′′), 4.42 (1H, q-like, *J* = 7.3 Hz, H-16), 4.37 (1H, br d, *J* = 12.5 Hz, H-6′′′′b), 4.36 (1H, d, *J* = 12.7 Hz, H-26b), 4.35 (1H, dd, *J* = 9.0, 9.0 Hz, H-4′′), 4.33 (1H, dd, *J* = 8.9, 8.9 Hz, H-4′), 4.32 (1H, dd, *J* = 9.3, 9.3 Hz, H-4′′′), 4.25 (1H, dd, *J* = 8.9, 8.9 Hz, H-4′′′′), 4.24 (1H, dd, *J* = 8.9, 8.9 Hz, H-3′′′′), 4.21 (1H, br d, *J* = 12.9 Hz, H-6′a), 4.20 (1H, dd, *J* = 8.9, 8.4 Hz, H-3′), 4.17 (1H, dd, *J* = 8.4, 7.2 Hz, H-2′), 4.16 (1H, m, H-2), 4.07 (1H, dd, *J* = 12.9, 4.6 Hz, H-6′b), 4.06 (1H, dd, *J* = 8.9, 7.8 Hz, H-2′′′′), 3.93 (1H, m, H-5′′′′), 3.82 (1H, m, H-3), 3.70 (1H, m, H-5′), 3.24 (3H, s, OMe), 1.67 (3H, d, *J* = 6.2 Hz, Me-6′′), 1.60 (3H, d, *J* = 6.2 Hz, Me-6′′′), 1.14 (3H, d, *J* = 6.9 Hz, Me-21), 1.04 (3H, s, Me-19), 0.77 (3H, s, Me-18). For ^13^C-NMR spectral data, see Table 1. For NMR spectral data, see Supplementary Materials.

Compound **6**: Amorphous solid; [α]_D_^25^ − 44.7 (*c* = 0.10, MeOH); IR (film) ν_max_: 3360 (OH), 2900 (CH) cm^−1^; HRESI-TOF-MS *m/z*: 915.4541 [M + H]^+^ (calcd. for C_45_H_71_O_19_: 915.4590). ^1^H-NMR spectral data (500 MHz, C_5_D_5_N): δ_H_ 5.36 (1H, br s, H-27a), 5.31 (1H, br d, *J* = 4.6 Hz, H-6), 5.25 (1H, d, *J* = 7.9 Hz, H-1′′), 5.05 (1H, br s, H-27b), 4.93 (1H, d, *J* = 7.7 Hz, H-1′), 4.90 (1H, d, *J* = 7.8 Hz, H-1′′′), 4.78 (1H, q-like, *J* = 7.7 Hz, H-16), 4.59 (1H, d, *J* = 12.5 Hz, H-26a), 4.35 (1H, d, *J* = 12.5 Hz, H-26b), 4.11 (1H, m, H-2), 3.82 (1H, m, H-3), 1.60 (3H, s, Me-21), 0.95 (3H, s, Me-19), 0.67 (3H, s, Me-18). For ^13^C-NMR spectral data, see Table 1. For NMR spectral data, see Supplementary Materials.

Dodecaacetate of Compound **6** (**6a**): ^1^H-NMR spectral data (500 MHz, C_5_D_5_N): δ_H_ 5.41 (1H, br s, H-27a), 5.40 (1H, br s, H-6), 5.06 (1H, br s, H-27b), 4.49 (1H, d, *J* = 12.5 Hz, H-26a), 4.30 (1H, d, *J* = 12.5 Hz, H-26b), 1.60 (3H, s, Me-21), 0.94 (3H, s, Me-19), 0.81 (3H, s, Me-18), 2.34, 2.17, 2.14, 2.12 × 2, 2.09, 2.05, 2.03, 2.00 × 3, 1.98 (each 3H, Ac × 12).

Compound **7**: Amorphous solid; [α]_D_^25^ − 51.0 (*c* = 0.10, MeOH); IR (film) ν_max_: 3380 (OH), 2919 (CH) cm^−1^; HRESI-TOF-MS *m*/*z*: 1045.5173 [M + H]^+^ (calcd. for C_51_H_81_O_22_: 1045.5219). ^1^H-NMR spectral data (500 MHz, C_5_D_5_N): δ_H_ 6.34 (1H, br s, H-1′′), 5.80 (1H, br s, H-1′′′), 5.36 (1H, br s, H-27a), 5.34 (1H, br d, *J* = 5.1 Hz, H-6), 5.04 (1H, br s, H-27b), 4.97 (1H, d, *J* = 6.6 Hz, H-1′), 4.90 (1H, d, *J* = 7.8 Hz, H-1′′′′), 4.78 (1H, q-like, *J* = 7.9 Hz, H-16), 4.59 (1H, d, *J* = 12.0 Hz, H-26a), 4.35 (1H, d, *J* = 12.0 Hz, H-26b), 4.16 (1H, m, H-2), 3.82 (1H, m, H-3), 1.67 (3H, d, *J* = 6.2 Hz, Me-6′′), 1.60 (3H, d, *J* = 6.1 Hz, Me-6′′′), 1.59 (3H, s, Me-21), 1.06 (3H, s, Me-19), 0.67 (3H, s, Me-18). For ^13^C-NMR spectral data, see Table 1. For NMR spectral data, see Supplementary Materials.

Compound **7a**: Amorphous solid; [α]_D_^25^ − 3.9 (*c* = 0.10, MeOH); IR (film) ν_max_: 3376 (OH), 2924 and 2852 (CH) cm^−1^; HRESI-TOF-MS *m*/*z*: 883.4738 [M + H]^+^ (calcd. for C_45_H_71_O_17_: 883.4691). ^1^H-NMR spectral data (500 MHz, C_5_D_5_N): δ_H_ 6.36 (1H, br s, H-1′′), 5.81 (1H, br s, H-1′′′), 5.35 (1H, br d, *J* = 4.6 Hz, H-6), 4.98 (1H, d, *J* = 7.2 Hz, H-1′), 4.81 (1H, br s, H-27a), 4.78 (1H, br s, H-27b), 4.53 (1H, m, H-16), 4.45 (1H, d, *J* = 12.1 Hz, H-26a), 4.16 (1H, m, H-2), 4.02 (1H, d, *J* = 12.1 Hz, H-26b), 3.83 (1H, m, H-3), 1.68 (3H, d, *J* = 6.2 Hz, Me-6′′), 1.61 (3H, d, *J* = 6.2 Hz, Me-6′′′), 1.07 (3H, d, *J* = 6.9 Hz, Me-21), 1.06 (3H, s, Me-19), 0.80 (3H, s, Me-18). ^13^C-NMR spectral data (125 MHz, C_5_D_5_N): δ_C_ 45.9, 70.2, 85.0, 37.1, 139.9, 121.9, 32.2, 31.1, 50.1, 37.9, 21.1, 39.6, 40.4, 56.4, 32.1, 81.4, 62.8, 16.2, 20.3, 41.8, 14.9, 109.4, 33.1, 28.9, 144.3, 64.9, 108.7 (C-1-27), 100.9, 77.7, 77.6, 78.6, 77.0, 61.0 (C-1′-6′), 101.9, 72.3, 72.7, 74.0, 69.5, 18.5 (C-1′′-6′′), 102.8, 72.4, 72.6, 73.8, 70.4, 18.4 (C-1′′′-6′′′).

Tridecaacetate of compound **7** (**7b**): ^1^H-NMR spectral data (500 MHz, C_5_D_5_N): δ_H_ 5.49 (1H, br s, H-6), 5.43 (1H, br s, H-27a), 5.34 (1H, br s, H-27b), 4.52 (1H, d, *J* = 13.1 Hz, H-26a), 4.32 (1H, d, *J* = 13.1 Hz, H-26b), 1.62 (3H, s, Me-21), 0.94 (3H, s, Me-19), 0.86 (3H, s, Me-18), 2.35, 2.19, 2.18, 2.16, 2.14, 2.09, 2.06, 2.04, 2.03 (×2), 2.01 (×2), 1.96 (each 3H, Ac × 13).

Compound **8**: Amorphous solid; [α]_D_^25^ − 48.9 (*c* = 0.10, MeOH); IR (film) ν_max_: 3381 (OH), 2932 (CH) cm^−1^; HRESI-TOF-MS *m*/*z*: 1035.5762 [M + H]^+^ (calcd. for C_51_H_87_O_21_: 1035.5740). ^1^H-NMR spectral data (500 MHz, C_5_D_5_N): δ_H_ 6.38 (1H, br s, H-1′′), 5.86 (1H, br s, H-1′′′), 5.34 (1H, br d, *J* = 4.9 Hz, H-6), 5.08 (1H, d, *J* = 7.8 Hz, H-1′′′′), 4.95 (1H, d, *J* = 7.7 Hz, H-1′). 3.91 (1H, m, H-22), 3.87 (1H, m, H-3), 1.77 (3H, d, *J* = 6.2 Hz, H-6′′), 1.62 (3H, d, *J* = 6.2 Hz, H-6′′′), 1.47 (3H × 2, each s, Me-26 and Me-27), 1.15 (3H, d, *J* = 6.7 Hz, Me-21), 1.07 (3H, s, Me-18), 0.70 (3H, s, Me-19). For ^13^C-NMR spectral data, see Table 1. For NMR spectral data, see Supplementary Materials.

Enzymatic hydrolysis of **1** and **4**–**8**: Compounds **1** (20 mg) and **8** (9.8 mg) were independently treated with naringinase (**1**: 168 mg, **8**: 102 mg) in AcOH/AcOK buffer (pH 4.3, 3.0 mL) at 28 °C for 132 h. Each reaction mixture was purified by silica gel CC eluted with CHCl_3_/MeOH/H_2_O (**1**; 9:1:0, 7:4:1, **8**; 9:1:0) to obtain **1a** (10.1 mg) from **1**, **8a** (3.0 mg) from **8**, and sugar fractions (6.1 mg from **1**, 2.4 mg from **8**), respectively. The sugar fraction was analyzed using HPLC under the following conditions: detection, refractive index, and optical rotation; column, Capcell Pak NH_2_ UG80 (4.6 mm i.d. × 250 mm, 5 μm, Shiseido); solvent, MeCN/H_2_O (17:3); flow rate, 1.0 mL/min. d-Galactose, d-glucose, and l-rhamnose were identified by comparing their retention times and optical rotations with those of authentic samples: d-galactose (12.13, positive optical rotation), d-glucose (13.62, positive optical rotation), and l-rhamnose (7.48, negative optical rotation). Compounds **4** (29.7 mg), **5** (10.1 mg), **6** (5.6 mg), and **7** (3.1 mg) were independently treated with β-d-glucosidase (**4**: 25 mg, **5**: 15 mg, **6**: 8.8 mg, **7**: 10 mg) in AcOH/AcONa (pH 5.0, 3.0 mL) at 28 °C for 20 h. Each reaction mixture was chromatographed on silica gel eluted with CHCl_3_/MeOH/H_2_O (**4**, **6**, and **7**; 20:10:1, **5**; 9:1:0) to collect **3** (17.4 mg) from **4**, **7a** (3.4 mg) from **5**, **3** (3.8 mg) from **6**, **7a** (1.0 mg) from **7**, and their sugar fractions. HPLC analysis of the sugar fractions under the same conditions as those of **1** exhibited the presence of d-galactose in **4** and **6**, d-glucose in **4**–**7**, and l-rhamnose in **5** and **7**.

Acid hydrolysis of **3** and **5**: Compounds **3** (18.2 mg) and **5** (10.1 mg) were independently treated with 1 M HCl (dioxane/H_2_O, 1:1, 3.0 mL) at 95 °C for 1 h under Ar atmosphere. The reaction solution was neutralized by passing through an Amberlite IRA-93 ZU (Organo, Tokyo, Japan) column and separated using a Sep-Pak C_18_ cartridge (Waters) eluted with MeOH/H_2_O (1:4) to yield sugar fractions (3.1 mg from **3** and 1.2 mg from **5**) and finally MeOH alone to obtain aglycone fractions. The sugar fractions were analyzed by HPLC under the same conditions as those of **1** showed the presence of d-galactose in **3**, d-glucose in **3** and **5**, and l-rhamnose in **5**. The aglycone fractions were independently subjected to silica gel CC eluted with CHCl_3_/MeOH (19:1) to furnish **3a** (8.3 mg) from **3**, and **3a** (2.8 mg) from **5**.

Acetylation of **4**–**7** and **1a**: Compounds **4** (100 mg) and **5** (4.5 mg) were independently applied to acetylation with Ac_2_O (2.0 mL) in pyridine (2.0 mL) at 130 °C for 3 h. The reaction solutions were distributed using Et_2_O (10 mL × 2). After concentration of the Et_2_O soluble phases, those were subjected to silica gel CC eluted with hexane hexane/Me_2_CO (1:1) to obtain **6a** (73. 0 mg) from **4**, and **7b** (3.4 mg) from **5**. Compounds **6** (10.2 mg), **7** (8.7 mg), and **1a** (8.8 mg) were independently acetylated with Ac_2_O (1.0 mL) in pyridine (1.0 mL) at 28 °C for 20 h. The reaction solutions were distributed and purified, as well as **4**, to afford **6a** (4.6 mg) from **6**, **7b** (4.1 mg) from **7**, and **1b** (4.0 mg) from **1a**.

### 3.5. Evaluation of Cytotoxic Activity

Cytotoxic activity of **1**–**11** against HL-60 cells (JCRB 0085; Human Science Research Resources Bank, Osaka, Japan) was examined by a modified MTT assay method as previously described [20]. In short, HL-60 cells were incubated at 37 °C for 24 h in RPMI-1640 medium with 10% heat-inactivated fetal bovine serum. The cell viability was evaluated using the MTT method.

## 4. Conclusions

A systematic phytochemical analysis of the leaves of *C. newellii* was conducted with a focus on steroidal glycosides. As a result, three new spirostanol glycosides (**1**–**3**), two new furostanol glycosides (**4** and **5**), two new pseudofurostanol glycosides (**6** and **7**), one new cholestane glycoside (**8**), and three known cholestane glycosides (**9**–**11**) were isolated. Compounds **1** and **2** are spirostanol glycosides having hydroxy groups at C-2, C-3, C-12, and C-24 of the aglycone moiety. Although *C. newellii* is known to be a poisonous plant, the MTT assay showed that none of the isolated compounds were cytotoxic toward HL-60 cells.

## Figures and Tables

**Table 1:** ^13^C-NMR (125MHz, C_5_D_5_N) spectral assignments for **1**, **1a**, **2**, **3**, **3a**, **4**–**8**.

Positions	1	1a	2	3	3a	4	5	6	7	8
1	45.7	46.5	45.7	45.8	46.5	45.8	45.9	45.8	46.0	37.5
2	69.9	72.5	69.9	70.0	72.6	70.0	70.1	70.0	70.1	30.2
3	84.5	76.7	84.5	84.5	76.7	84.5	84.9	84.6	84.9	78.1
4	37.4	40.7	37.5	37.5	40.8	37.5	37.1	37.6	37.1	39.0
5	140.0	141.2	140.0	140.0	141.2	140.0	139.8	140.0	139.8	140.8
6	121.9	121.3	121.9	121.8	121.2	121.8	121.9	121.8	121.9	122.0
7	31.9	32.0	31.9	32.1	32.2	32.0	32.0	32.2	32.3	32.2
8	30.2	30.3	30.2	31.0	31.1	30.9	31.0	30.8	30.8	32.1
9	49.8	50.1	49.8	50.1	50.3	50.1	50.1	50.1	50.1	50.4
10	37.9	38.6	38.0	37.8	38.4	37.8	37.8	37.8	37.8	36.9
11	31.4	31.5	31.4	21.1	21.2	21.0	21.0	21.3	21.2	21.3
12	78.7	78.9	78.7	39.6	39.7	39.5	39.5	39.5	39.5	40.1
13	46.1	46.2	46.2	40.4	40.4	40.3	40.3	43.2	43.2	42.3
14	55.2	55.4	55.2	56.4	56.5	56.3	56.3	54.7	54.7	57.0
15	31.7	31.8	31.7	32.0	32.1	32.1	32.1	34.4	34.4	24.5
16	81.4	81.5	81.8	81.3	81.4	81.3	81.3	84.4	84.4	28.2
17	62.2	62.3	62.2	62.7	62.8	63.9	63.9	64.4	64.4	53.1
18	10.9	11.0	11.0	16.2	16.3	16.0	16.1	14.1	14.0	12.0
19	20.3	20.6	20.3	20.3	20.6	20.3	20.3	20.4	20.3	19.4
20	43.2	43.2	43.0	41.7	41.8	40.7	40.7	103.9	103.9	41.9
21	14.2	14.2	14.1	14.9	14.9	16.1	16.1	11.7	11.7	12.5
22	112.0	112.1	112.0	109.4	109.4	112.3	112.3	151.6	151.6	73.2
23	41.8	41.9	43.5	33.1	33.1	31.5	31.5	24.6	24.6	30.5
24	70.6	70.6	67.0	28.8	28.9	28.0	28.0	31.0	31.0	39.3
25	39.8	39.9	149.3	144.3	144.4	146.7	146.7	146.1	146.1	77.4
26	65.2	65.3	64.6	64.9	64.9	71.9	71.9	71.6	71.6	27.1
27	13.6	13.6	106.4	108.7	108.7	111.0	111.0	111.6	111.6	27.1
OMe						47.3	47.3			
	Gal		Gal	Gal		Gal	Glc (I)	Gal	Glc (I)	Glc (I)
1′	103.2		103.3	103.3		103.3	100.9	103.4	100.9	100.2
2′	73.0		73.0	73.0		73.0	77.6	73.0	77.7	77.8
3′	75.0		75.1	75.1		75.0	77.6	75.1	77.7	77.9
4′	79.9		80.0	80.0		80.0	78.5	80.0	78.5	78.5
5′	75.8		75.9	75.9		75.8	76.9	75.9	77.0	76.9
6′	60.9		60.9	60.9		60.9	61.0	60.9	61.0	61.2
	Glc		Glc	Glc		Glc (I)	Rha (I)	Glc (I)	Rha (I)	Rha (I)
1′′	106.9		106.9	106.9		106.9	101.9	107.0	101.9	102.0
2′′	75.7		75.7	75.7		75.7	72.2	75.7	72.3	72.5
3′′	78.5		78.6	78.6		78.6	72.7	78.6	72.7	72.8
4′′	72.0		72.1	72.0		72.0	73.9	72.1	74.0	74.1
5′′	78.3		78.4	78.4		78.3	69.4	78.4	69.5	69.5
6′′	62.8		62.9	62.9		62.9	18.5	63.0	18.5	18.6
						Glc (II)	Rha (II)	Glc (II)	Rha (II)	Rha (II)
1′′′						103.7	102.7	103.7	102.7	102.8
2′′′						75.0	72.3	75.1	72.4	72.4
3′′′						78.5	72.6	78.5	72.6	72.7
4′′′						71.6	73.8	71.6	73.8	73.9
5′′′						78.4	70.3	78.4	70.3	70.4
6′′′						62.7	18.4	62.7	18.4	18.5
							Glc (II)		Glc (II)	Glc (II)
1′′′′							103.7		103.7	98.6
2′′′′							75.0		75.1	75.4
3′′′′							78.5		78.5	78.8
4′′′′							71.6		71.6	71.8
5′′′′							78.4		78.4	78.0
6′′′′							62.7		62.6	62.9

## Supplementary Materials

The following are available online. Figures S1–S52 showed NMR spectral data of **1**, **1a**, **2**, **3**, **3a**, **4**–**11**.
