# Supplementary material for: A Total of Eight Novel Steroidal Glycosides Based on Spirostan, Furostan, Pseudofurostan, and Cholestane from the Leaves of Cestrum newellii
Source: Molecules. 2020 Sep 28;25(19):4462. doi: 10.3390/molecules25194462 (PMC7582601; doi:10.3390/molecules25194462)
Supplement: Supplementary file 1 [file molecules-25-04462-s001.pdf]

Supplementary material

A Total of Eight Novel Steroidal Glycosides Based on Spirostan, Furostan, Pseudofurostan, and Cholestane from the Leaves of *Cestrum newellii*

Tomoki Iguchi *, Naoki Takahashi, and Yoshihiro Mimaki

School of Pharmacy, Tokyo University of Pharmacy and Life Sciences, 1432-1, Horinouchi, Hachioji, Tokyo 192-0392, Japan; y20612@toyaku.ac.jp (N.T.); mimakiy@toyaku.ac.jp (Y.M.)

* Correspondence: iguchit@toyaku.ac.jp; Tel. +81-42-676-4575

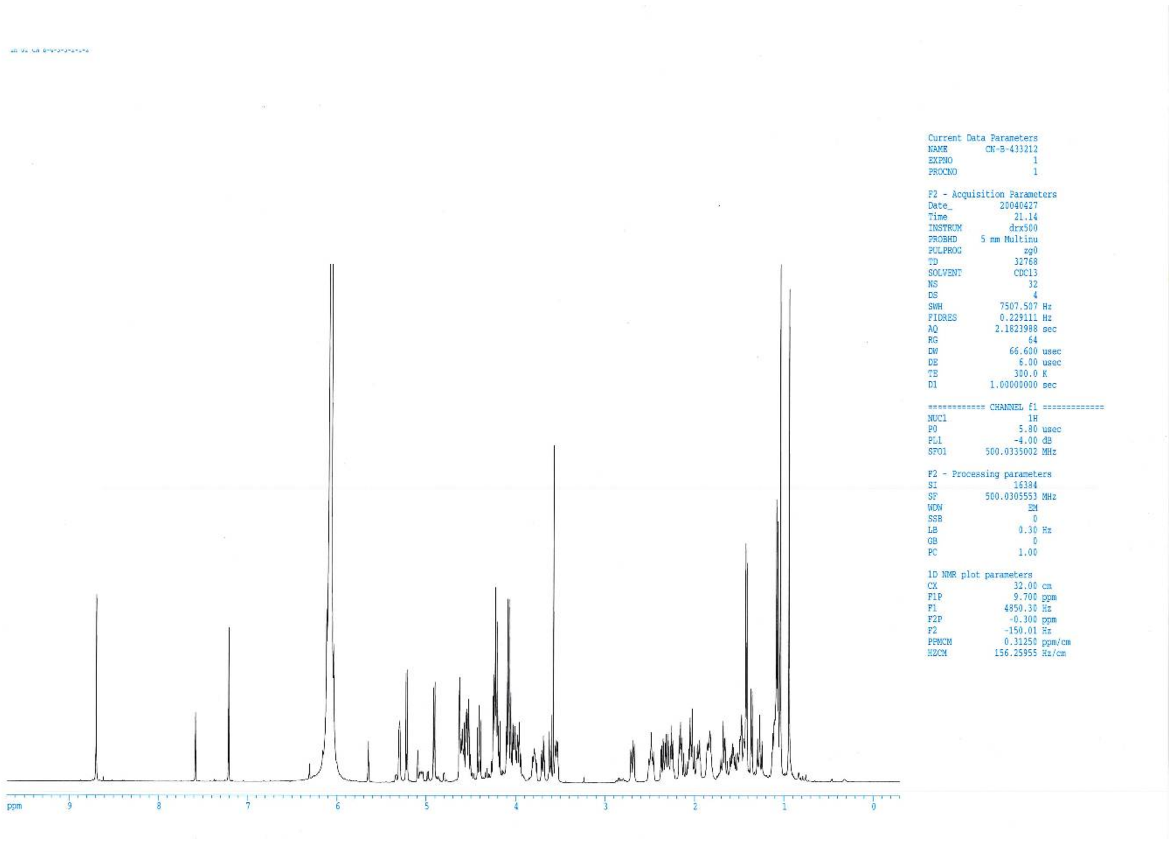

Figure S1. ^{1}H-NMR spectrum of **1**

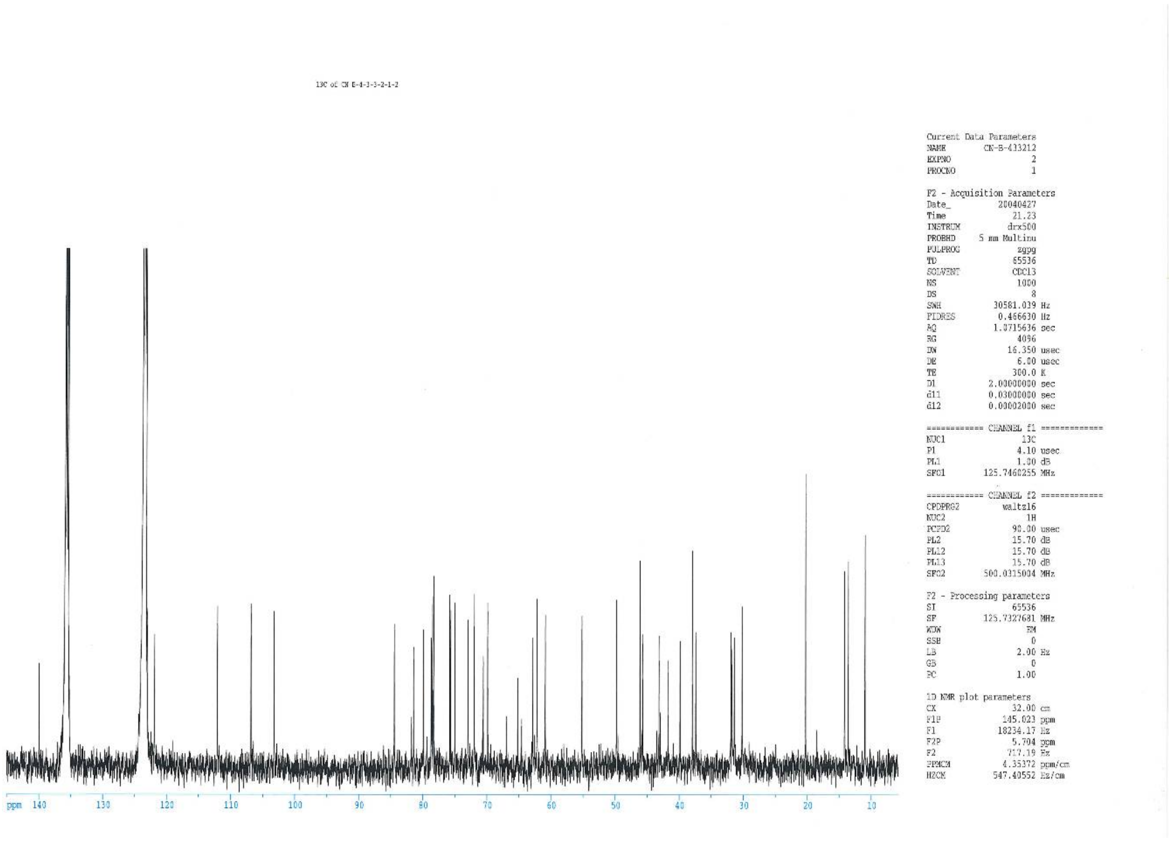

Figure S2. ^{13}C-NMR spectrum of **1**

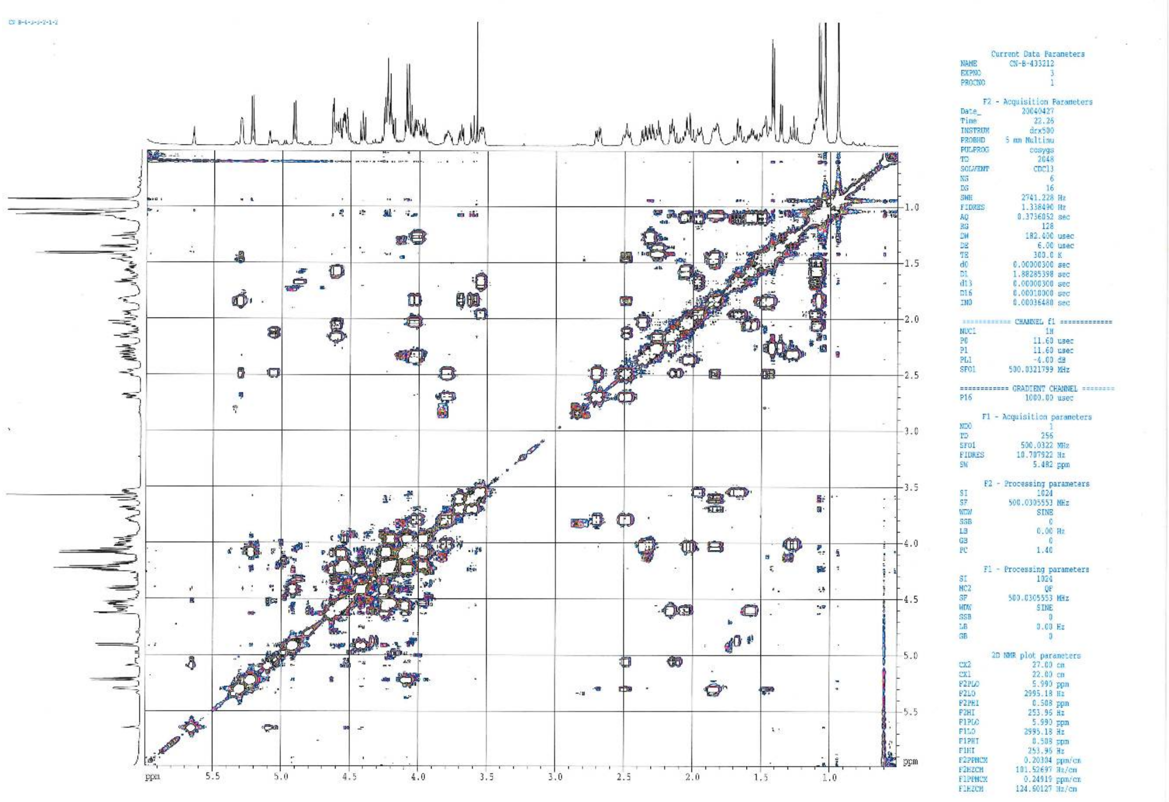

Figure S3. ^{1}H-^{1}H COSY spectrum of **1**

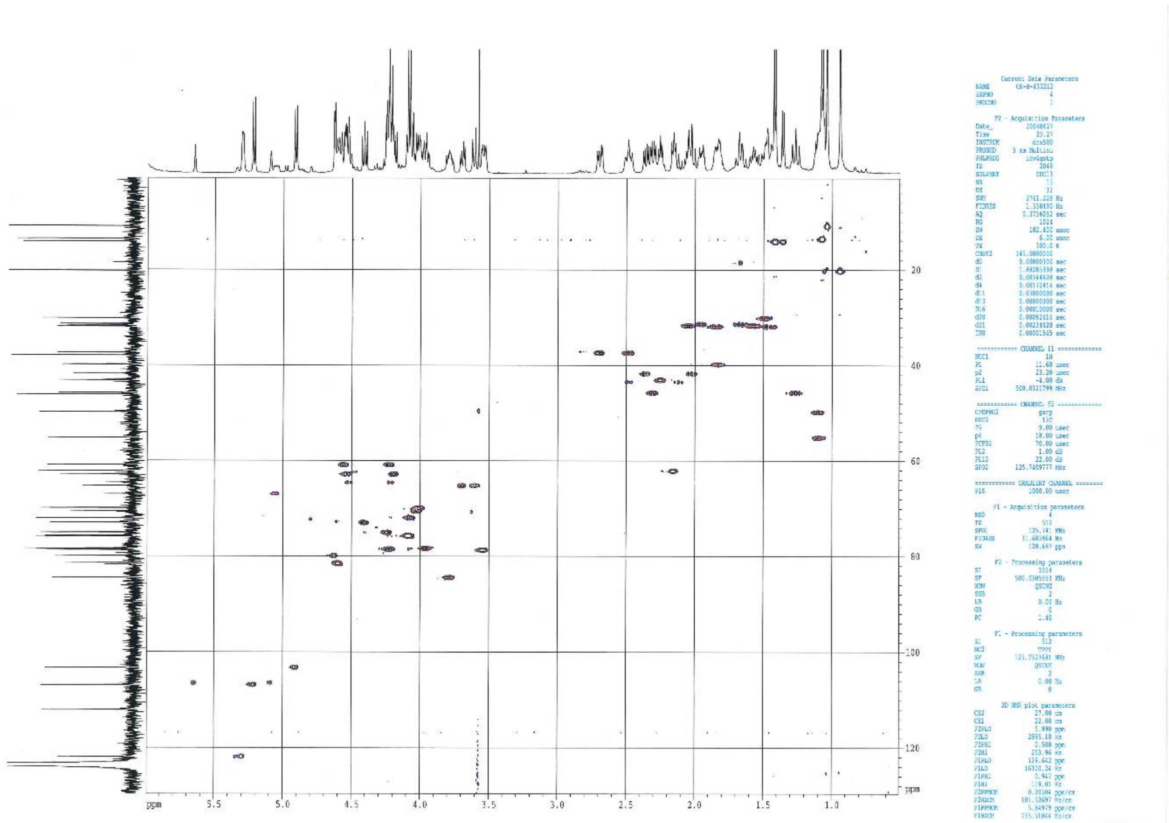

Figure S4. HMQC spectrum of **1**

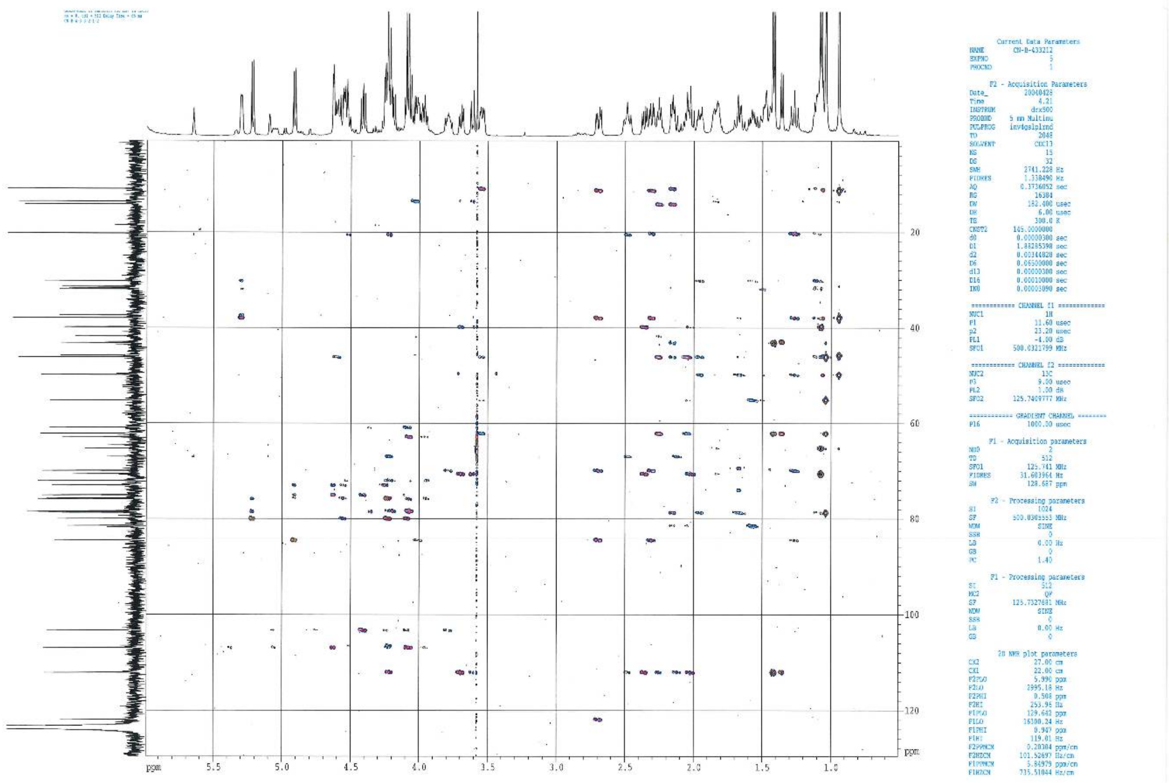

Figure S5. HMBC spectrum of **1**

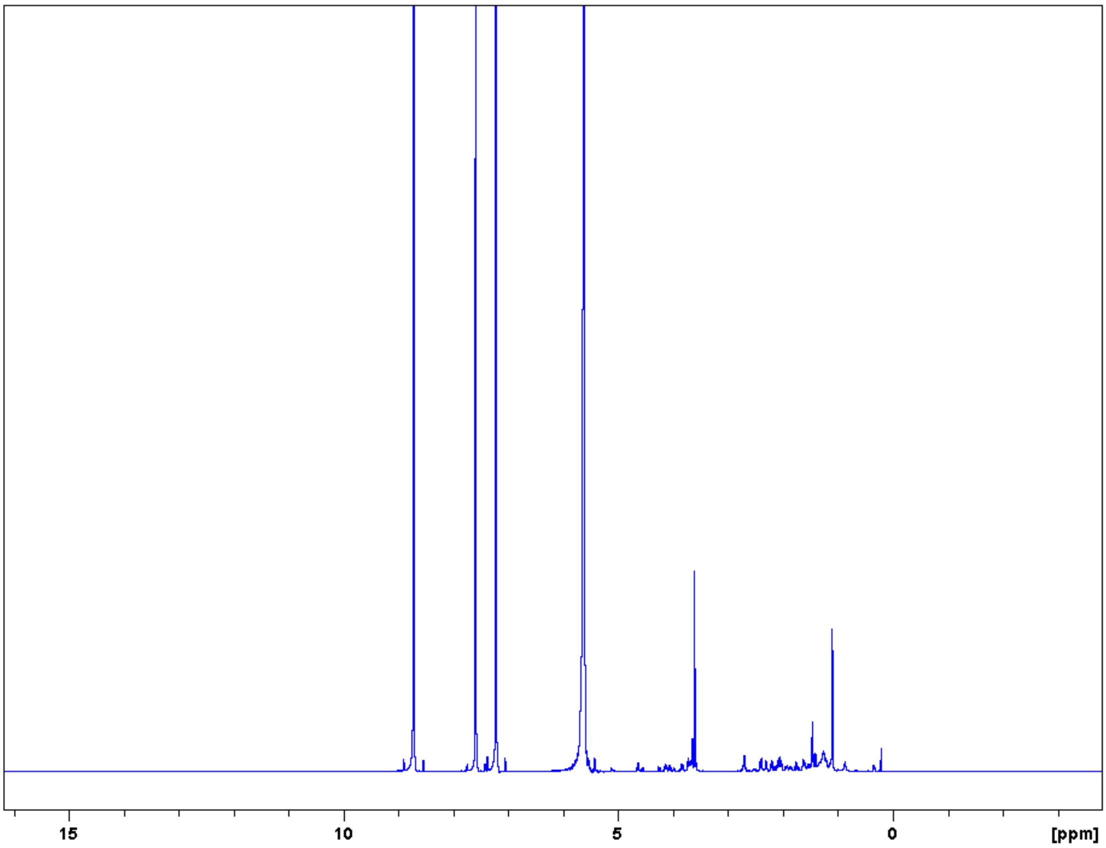

Figure S6. ^{1}H-NMR spectrum of **1a**

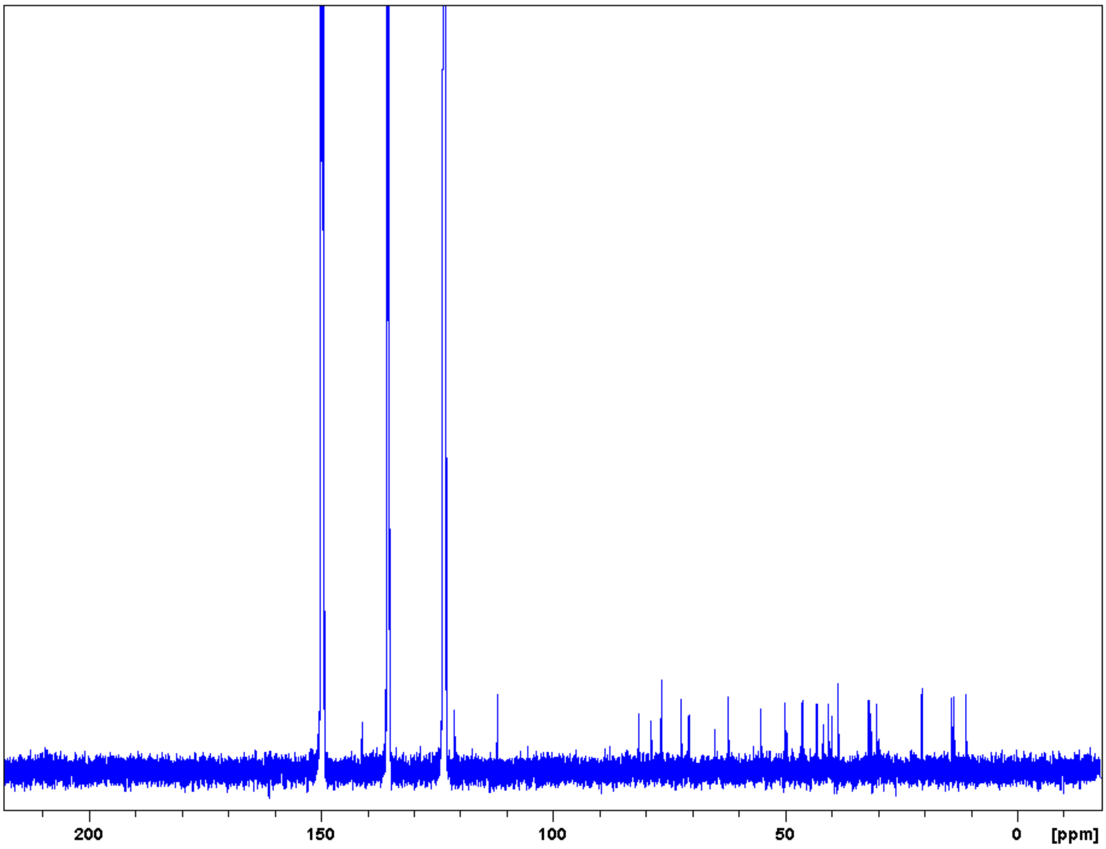

Figure S7. ^{13}C-NMR spectrum of **1a**

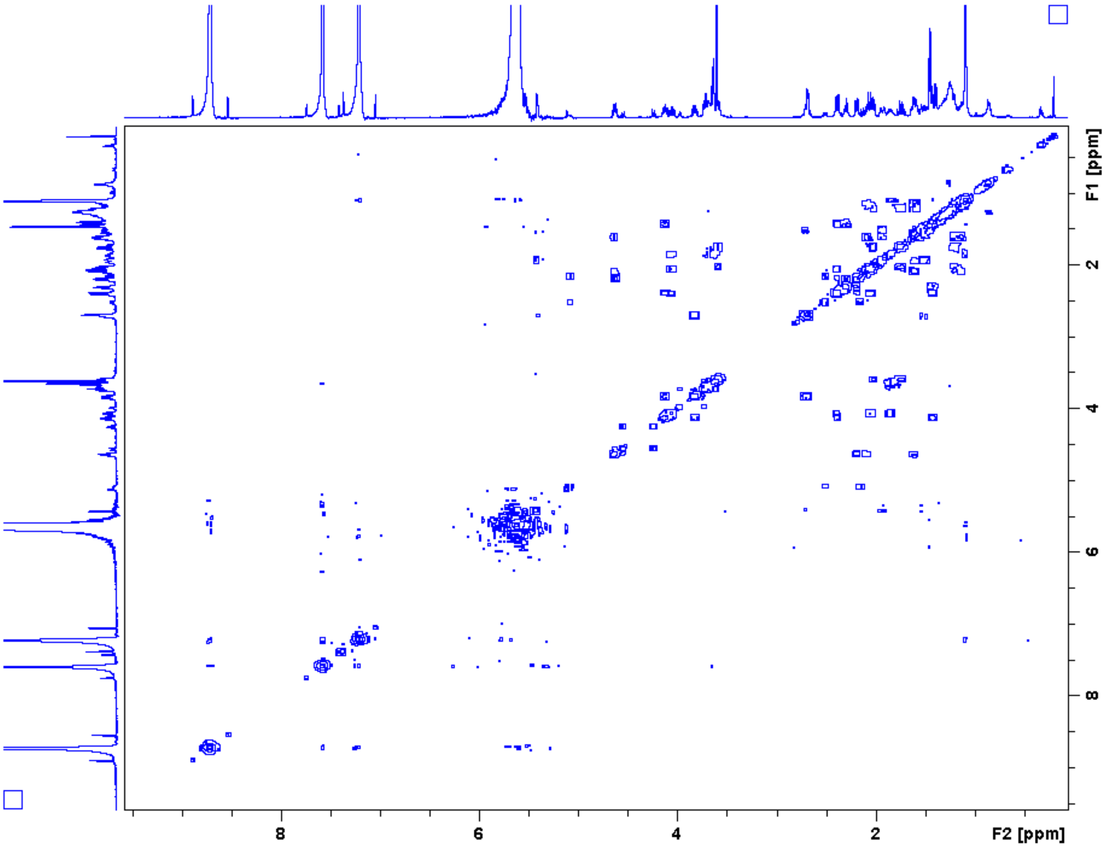

Figure S8. ^{1}H-^{1}H COSY spectrum of **1a**

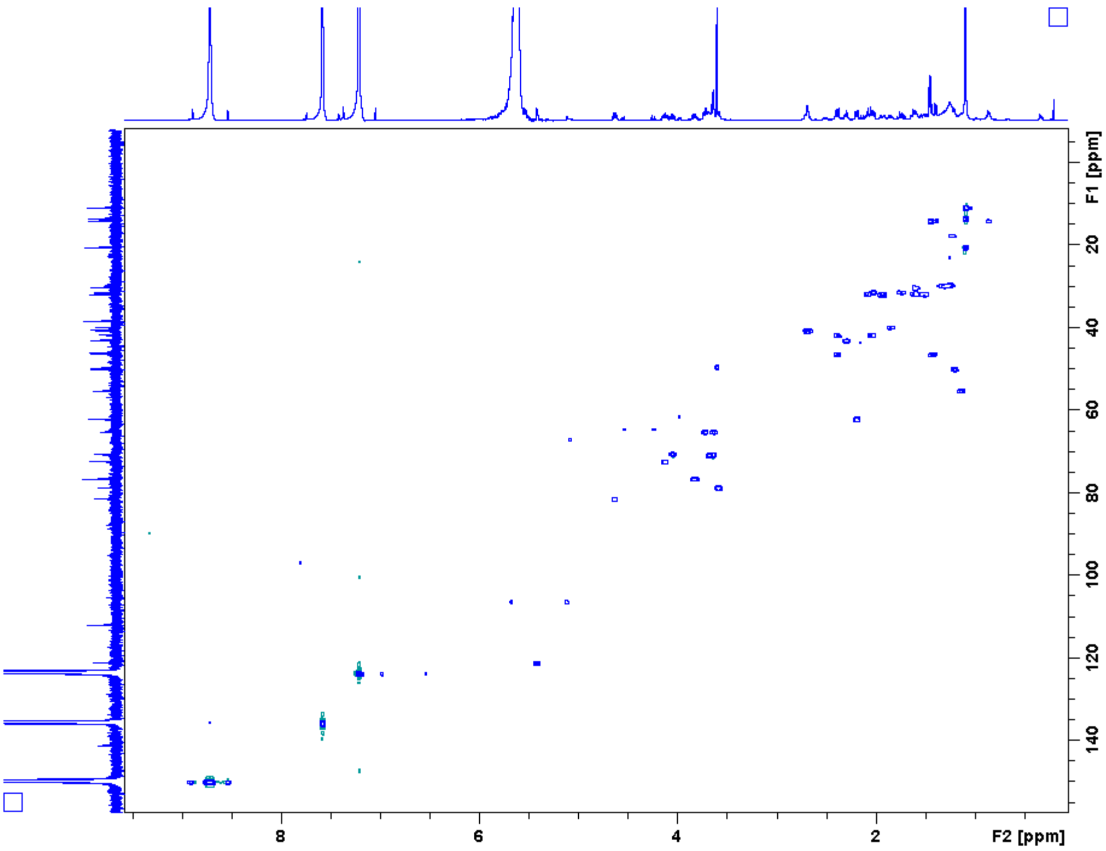

Figure S9. HMQC spectrum of **1a**

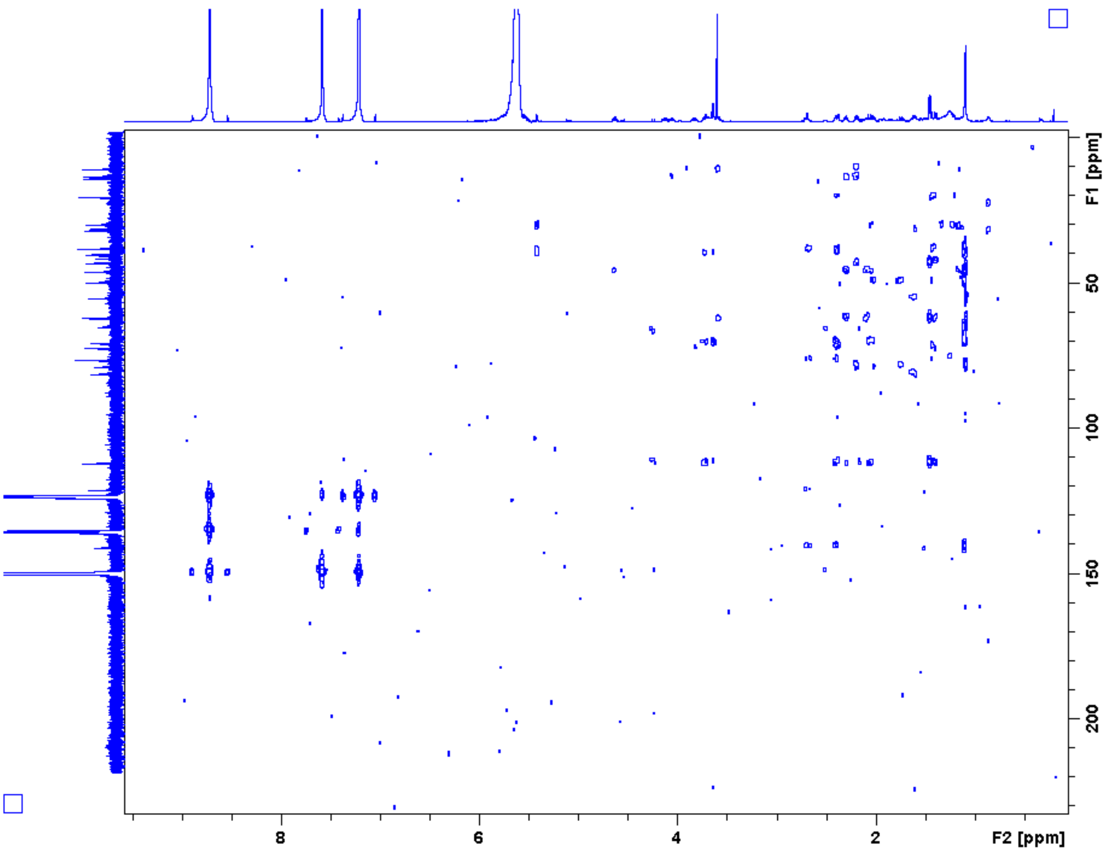

Figure S10. HMBC spectrum of **1a**

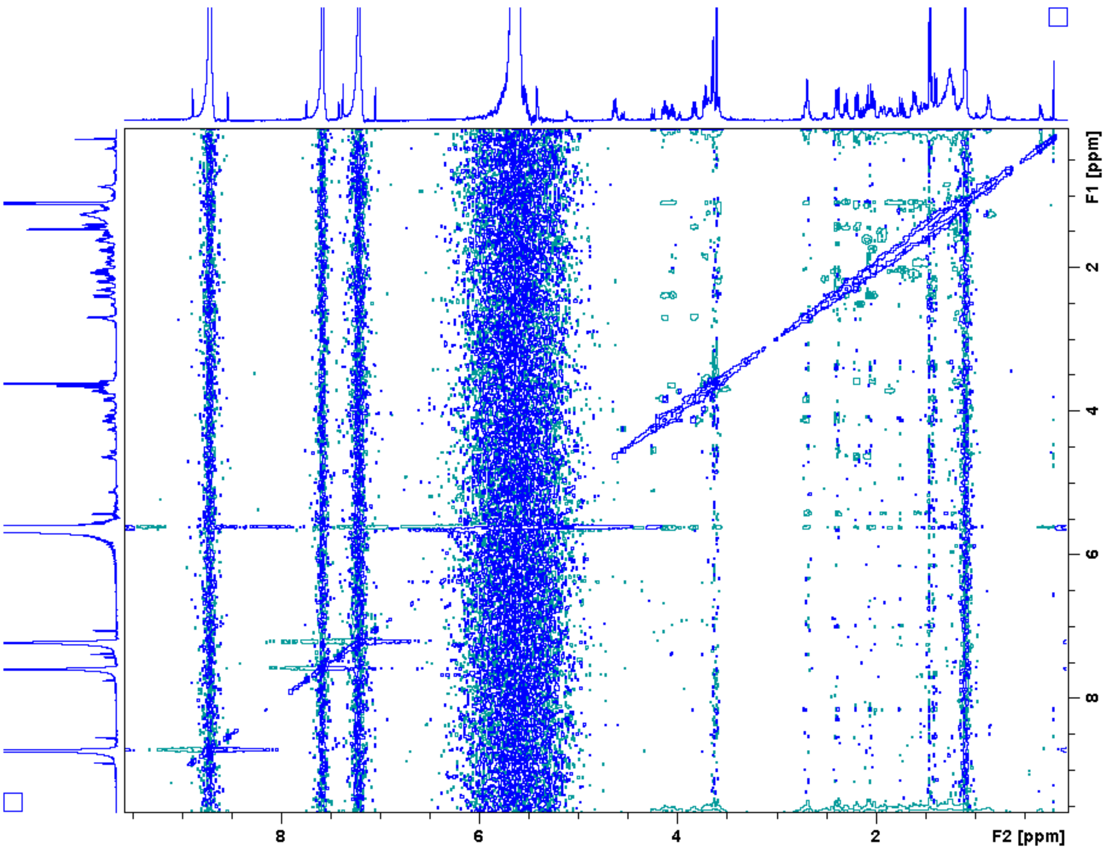

Figure S11. NOESY spectrum of **1a**

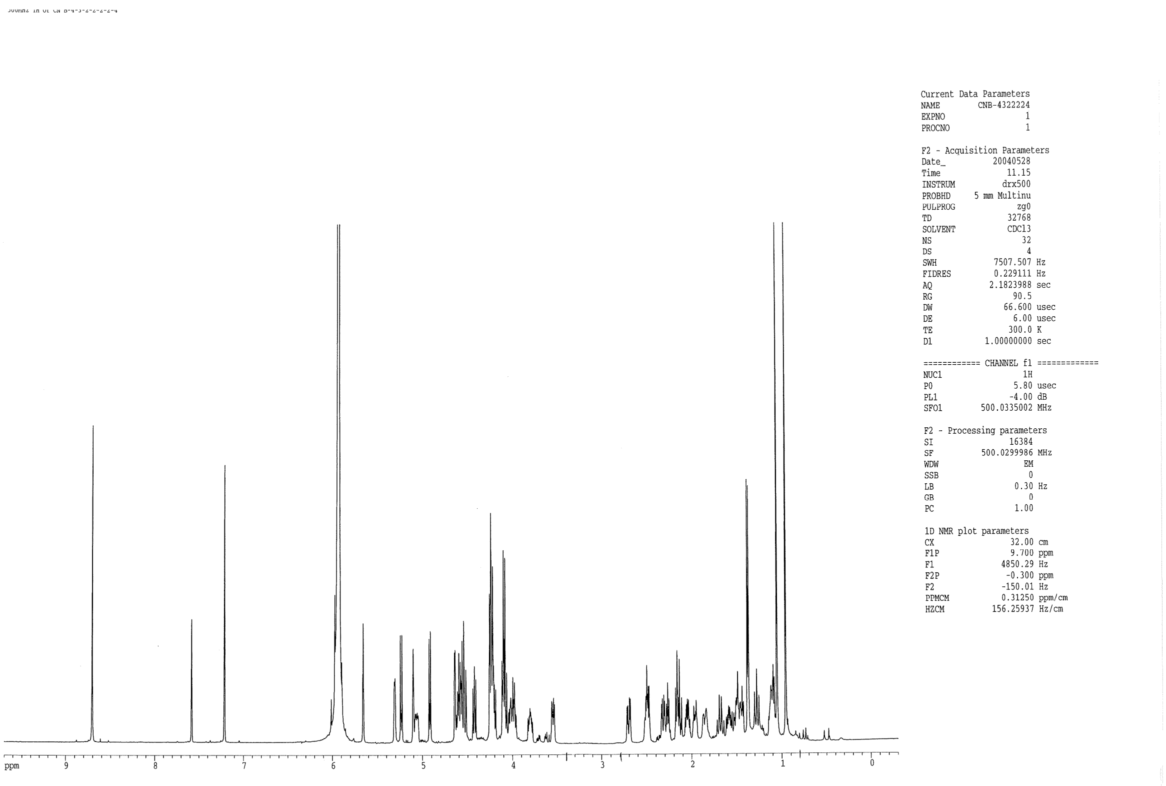

Figure S12. ^{1}H-NMR spectrum of **2**

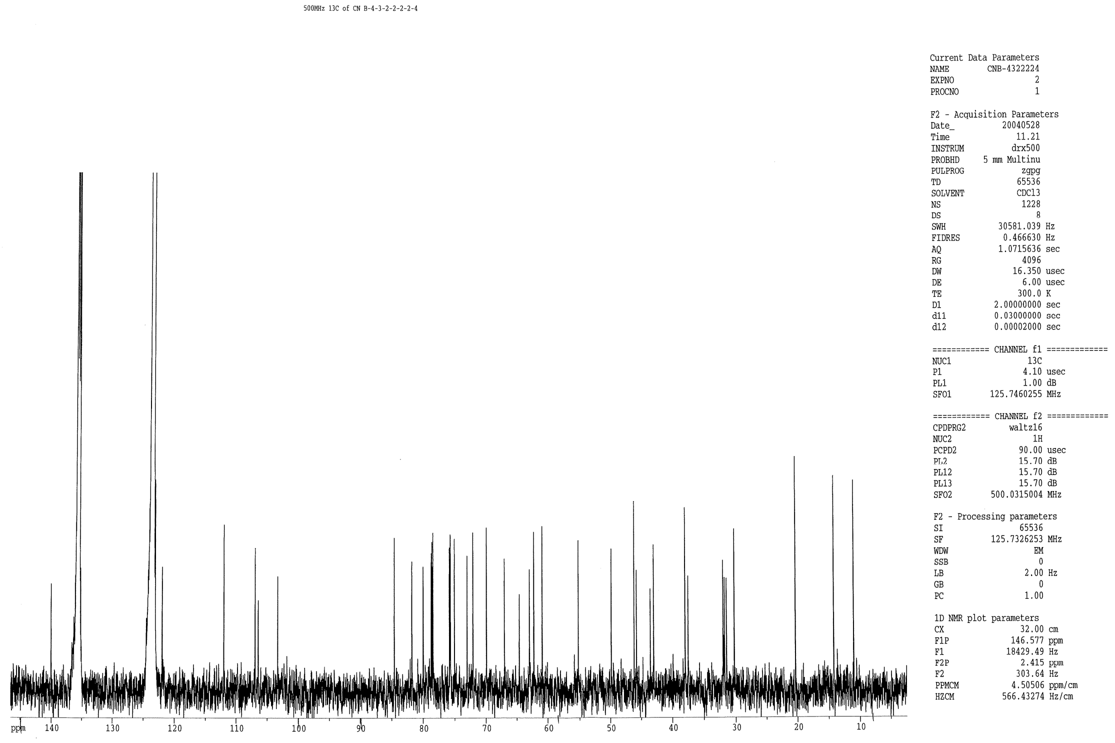

Figure S13. ^{13}C-NMR spectrum of **2**

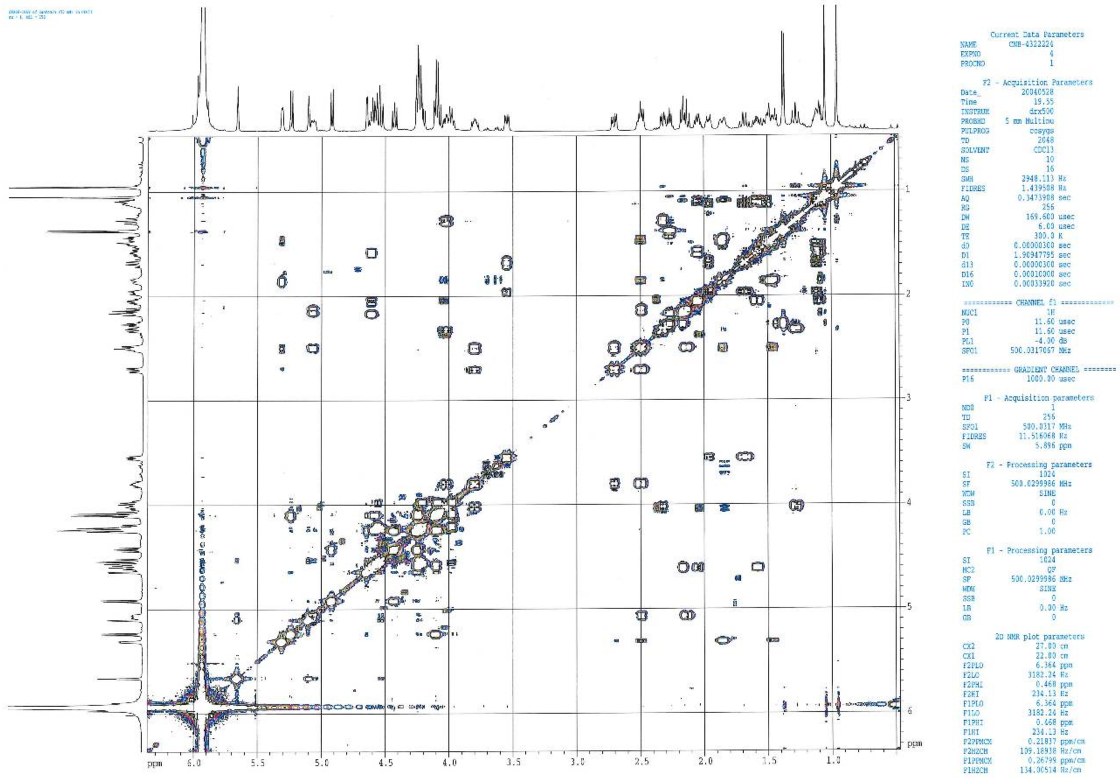

Figure S14. ^{1}H-^{1}H COSY spectrum of **2**

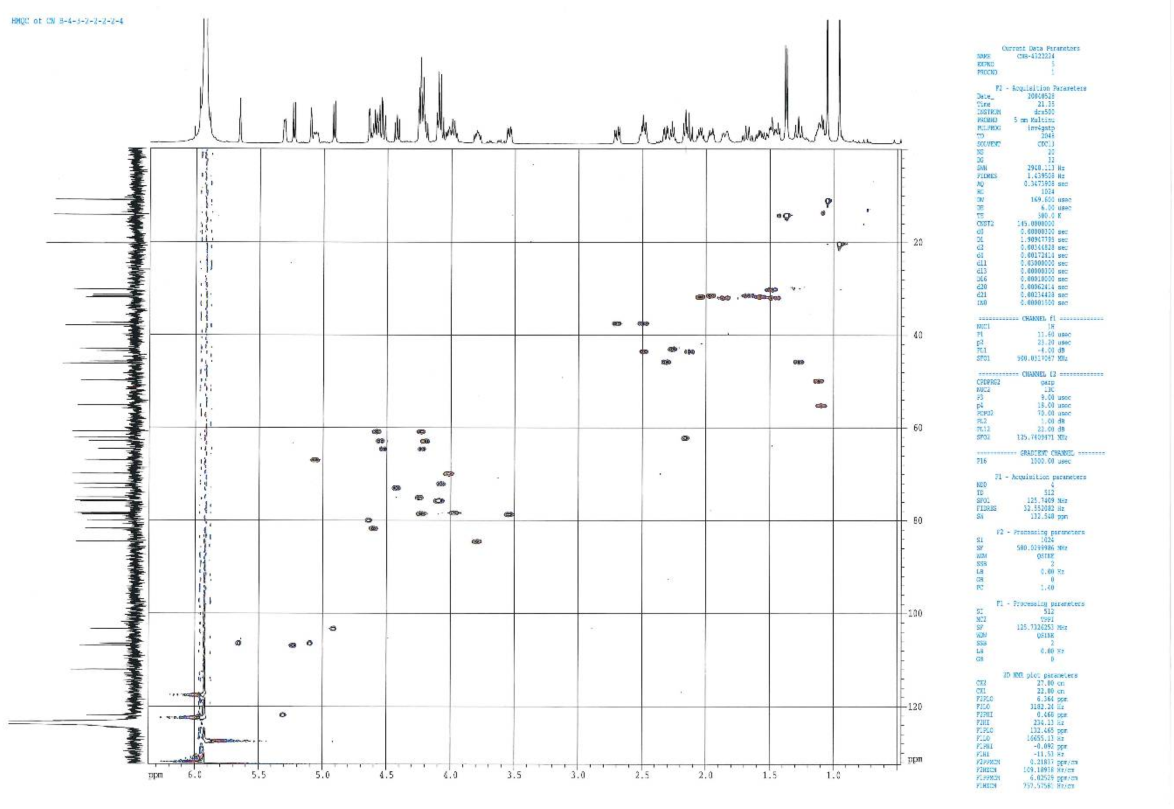

Figure S15. HMQC spectrum of **2**

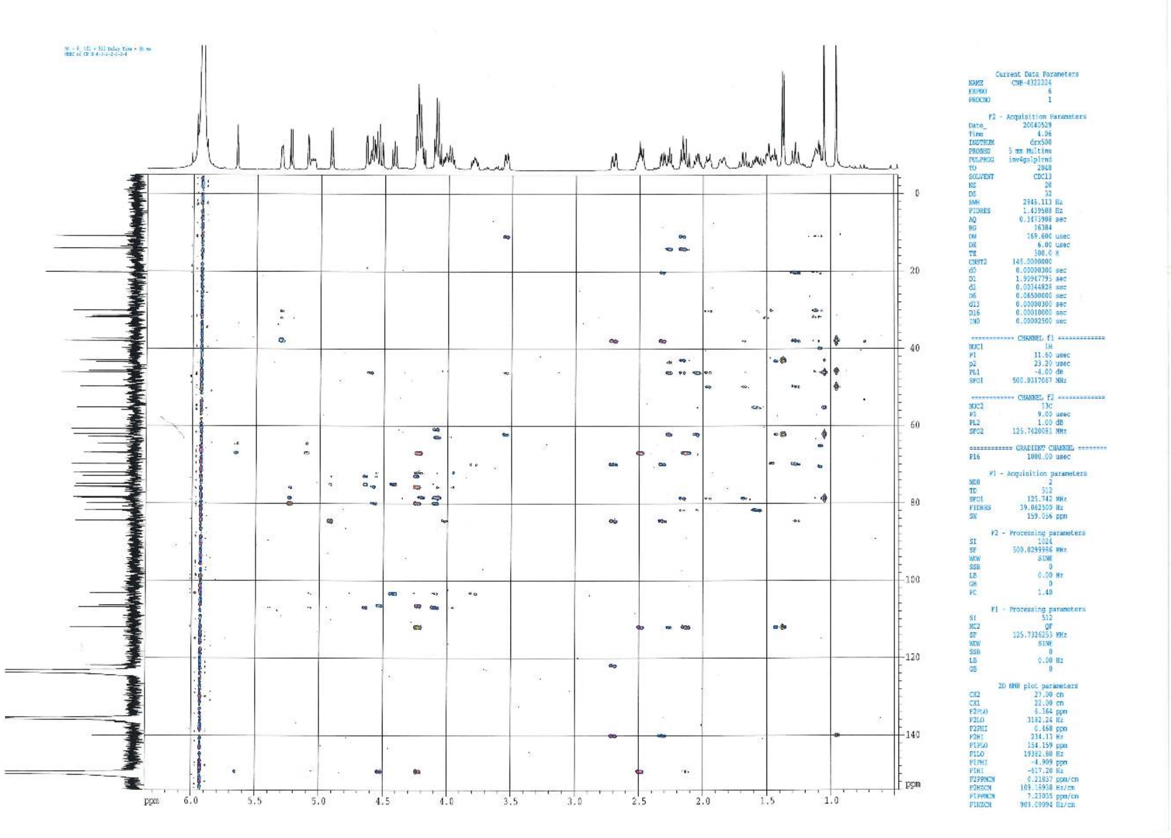

Figure S16. HMBC spectrum of **2**

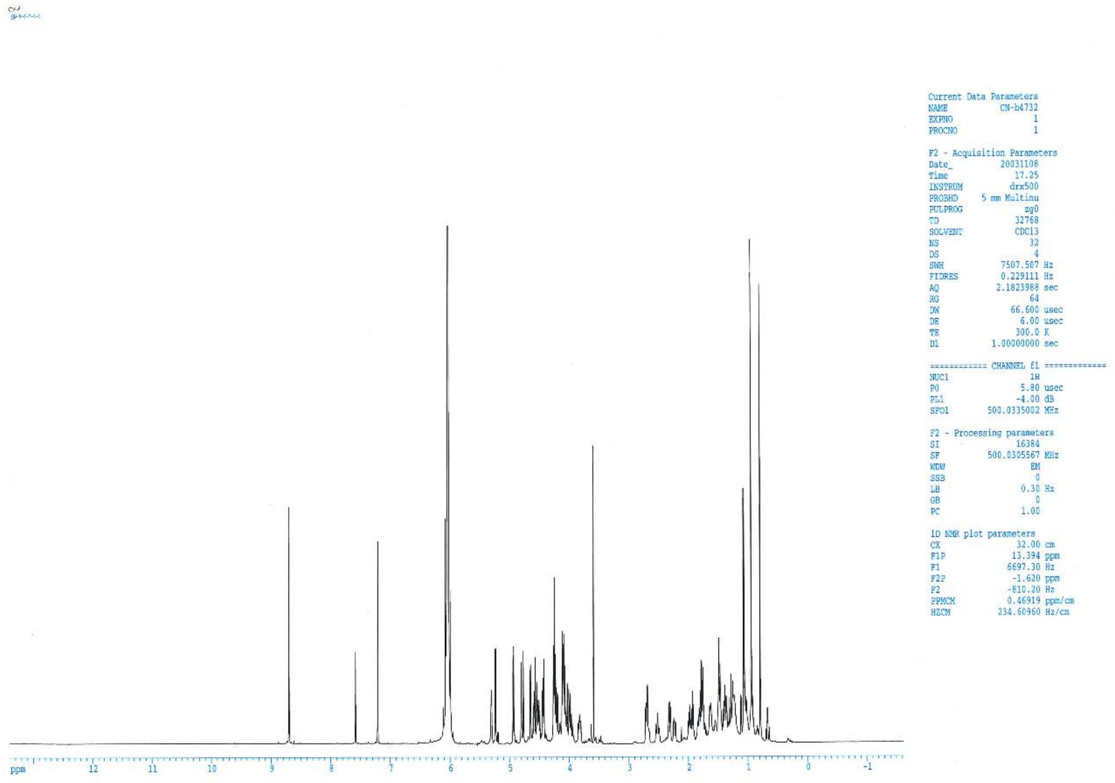

Figure S17. ^{1}H-NMR spcetrum of **3**

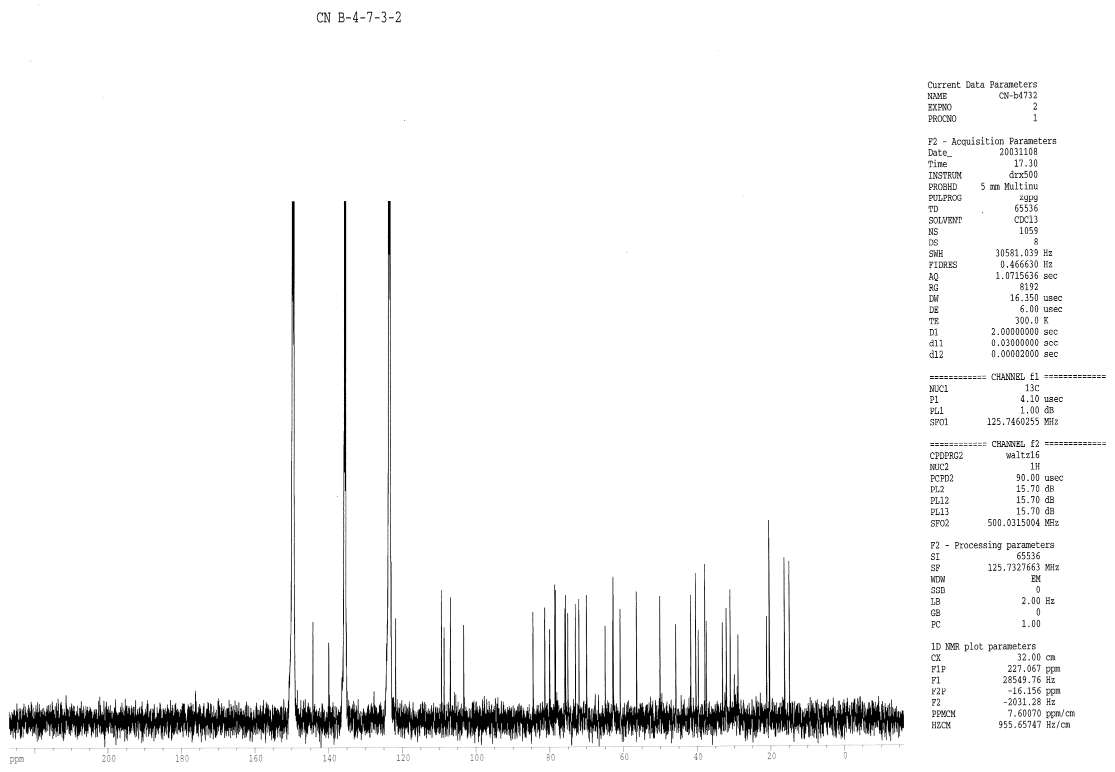

Figure S18. ^{13}C-NMR spectrum of **3**

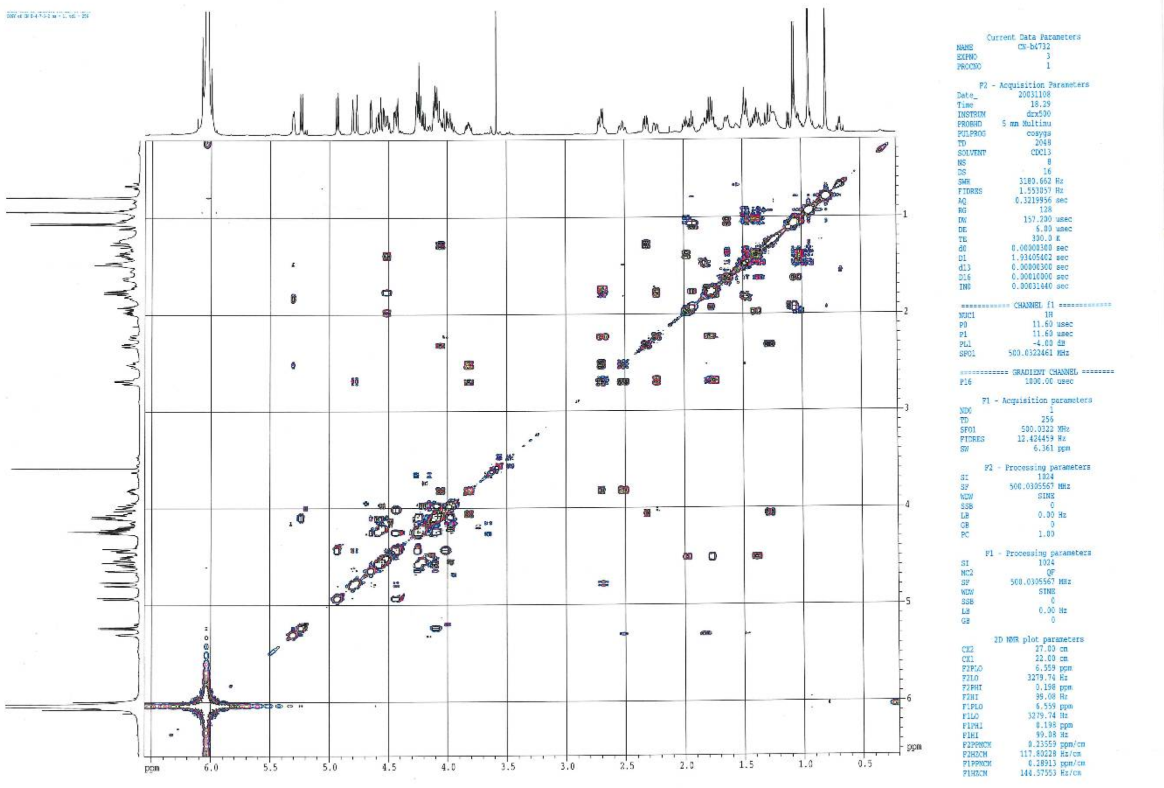

Figure S19. ^{1}H-^{1}H COSY spectrum of **3**

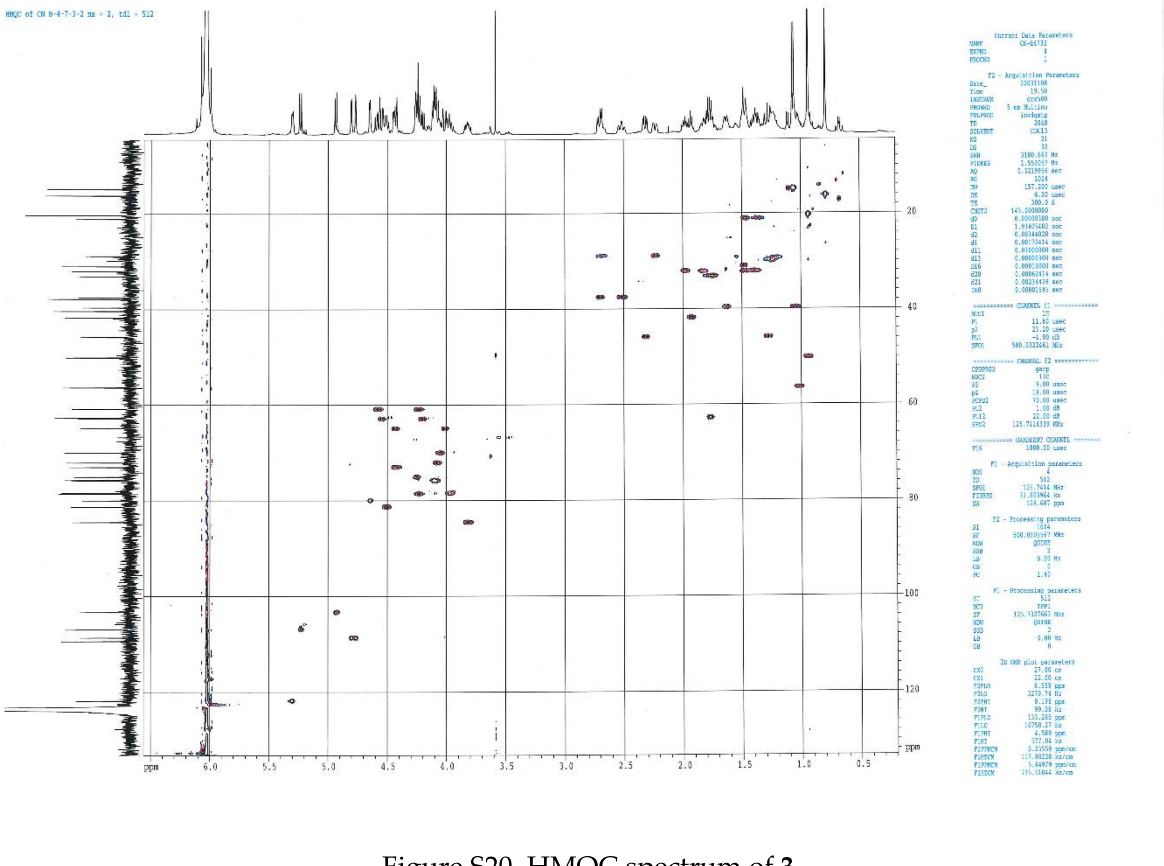

Figure S20. HMQC spectrum of **3**

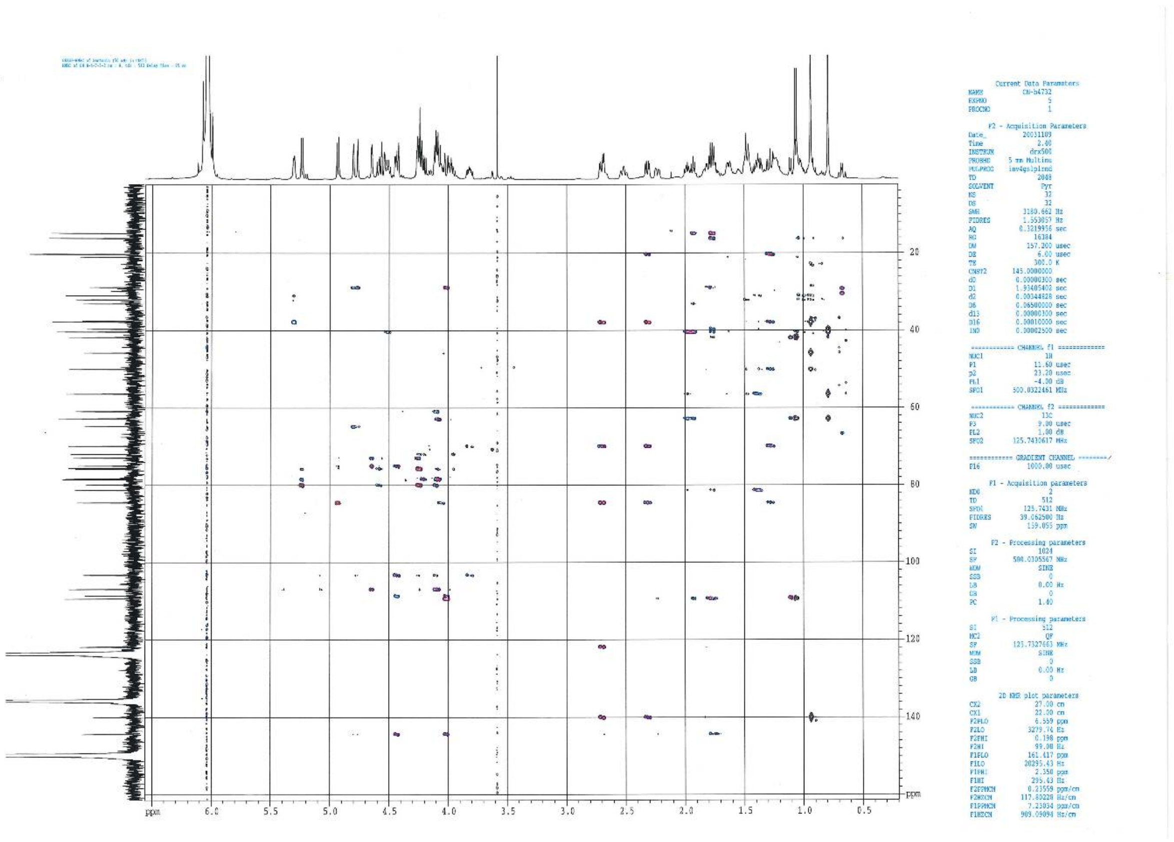

Figure S21. HMBC spectrum of **3**

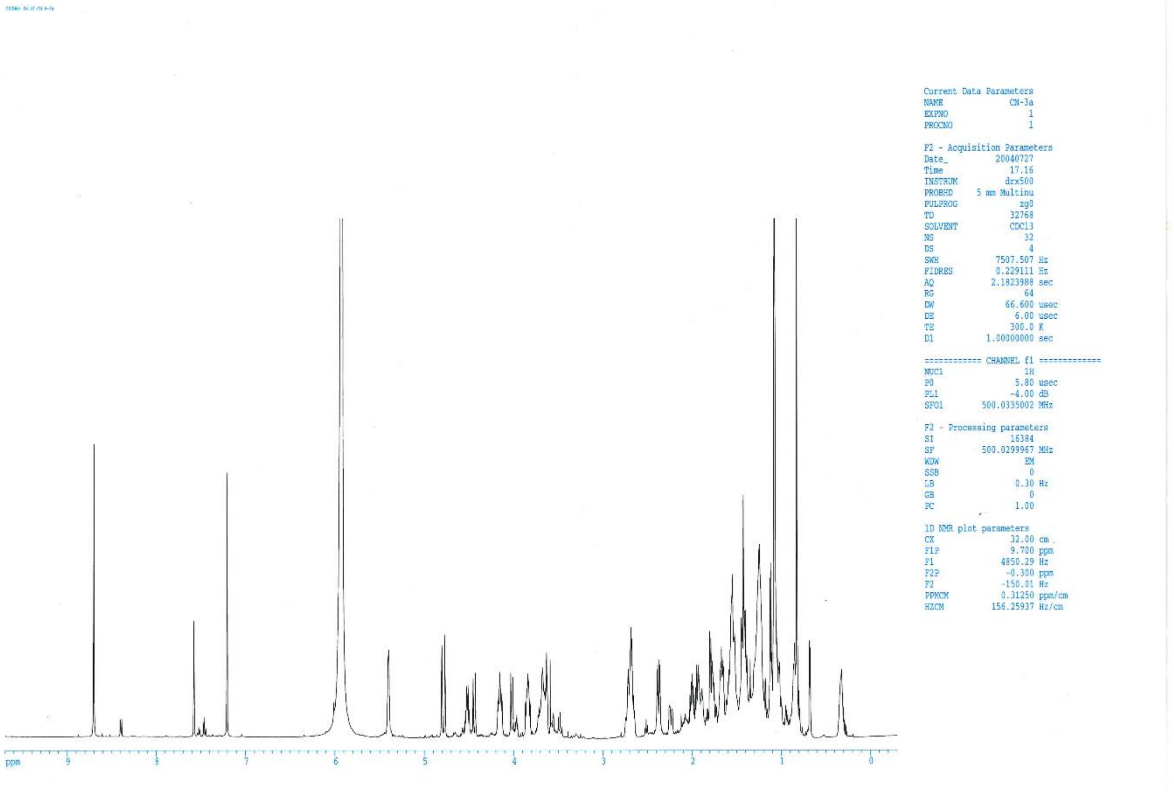

Figure S22. ^{1}H-NMR spectrum of **3a**

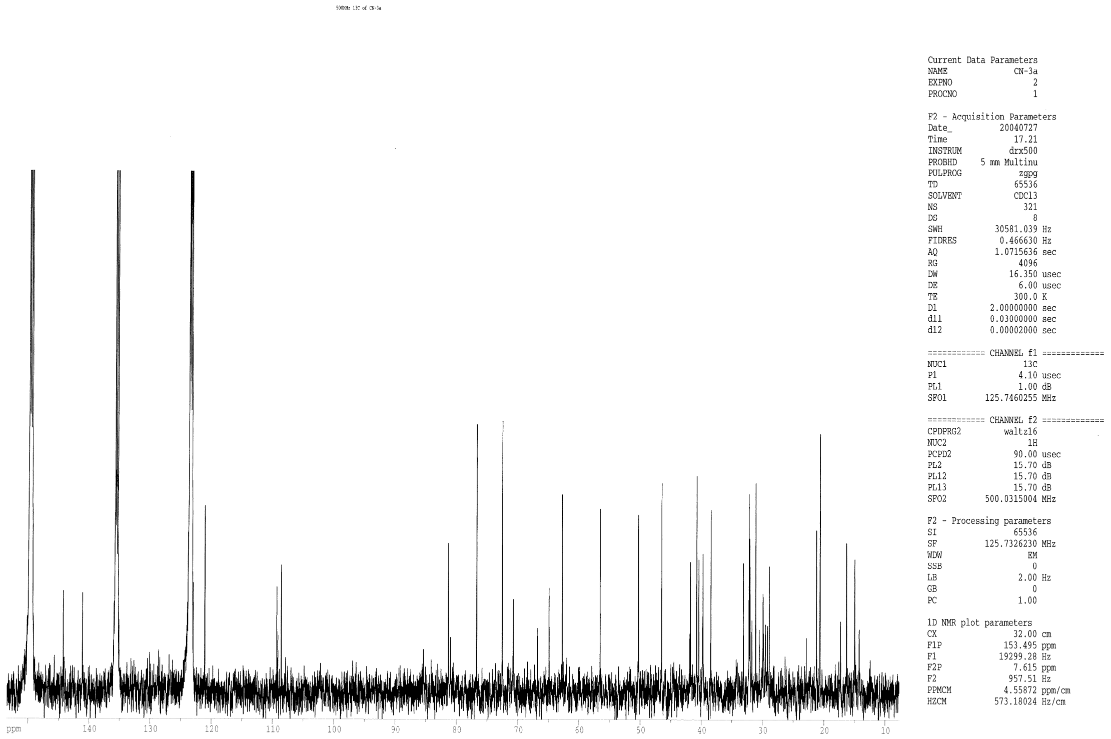

Figure S23. ^{13}C-NMR spectrum of **3a**

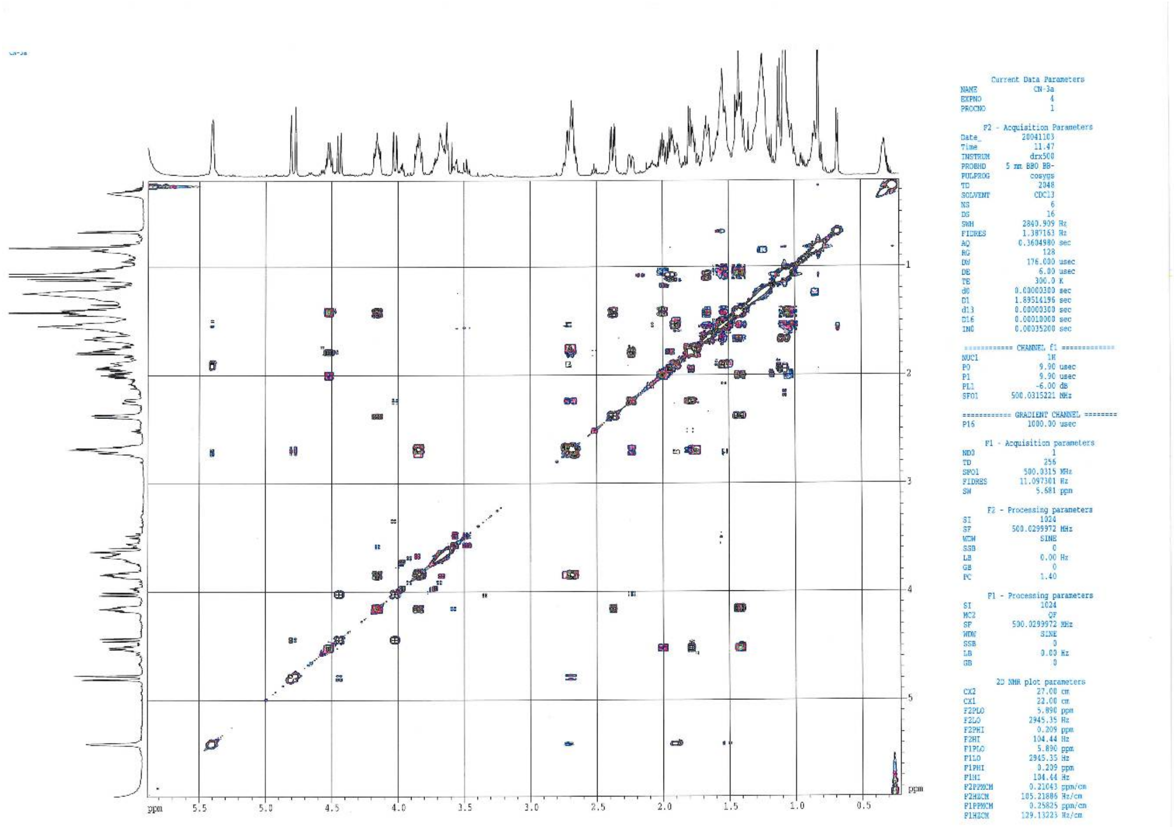

Figure S24. ^{1}H-^{1}H COSY spectrum of **3a**

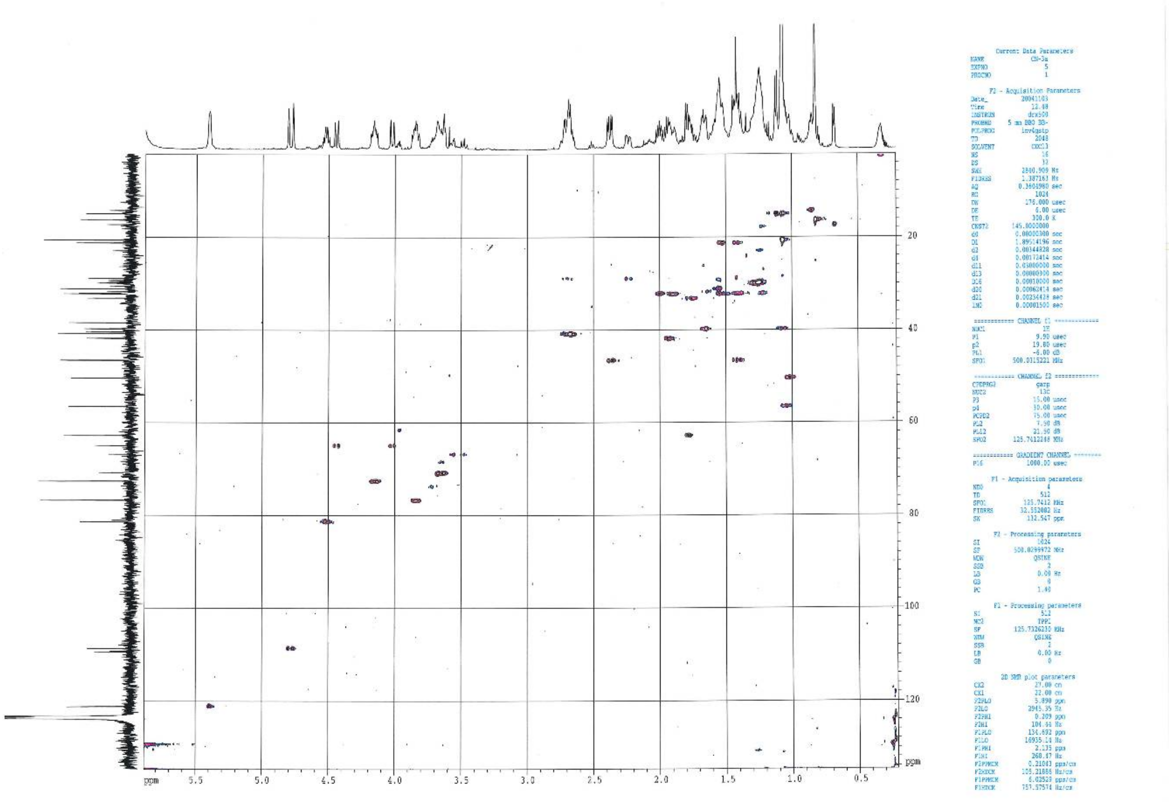

Figure S25. HMQC spectrum of **3a**

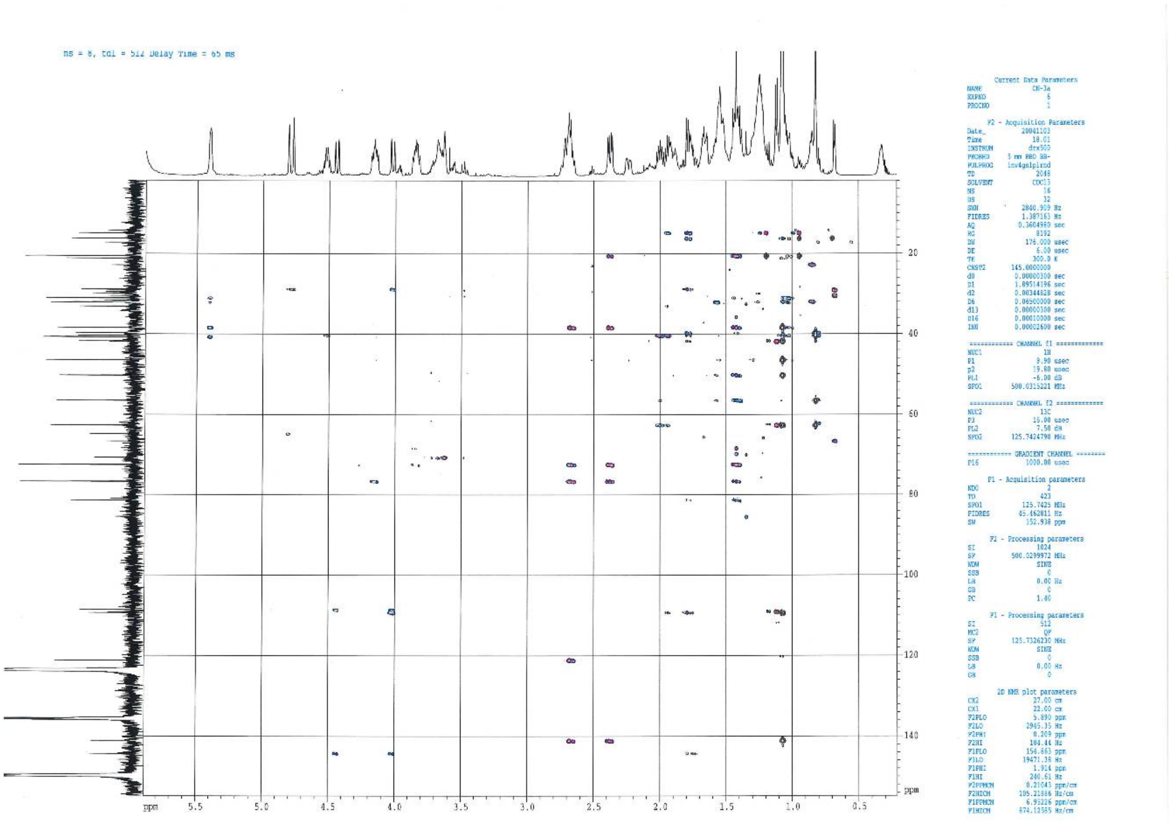

Figure S26. HMBC spectrum of **3a**

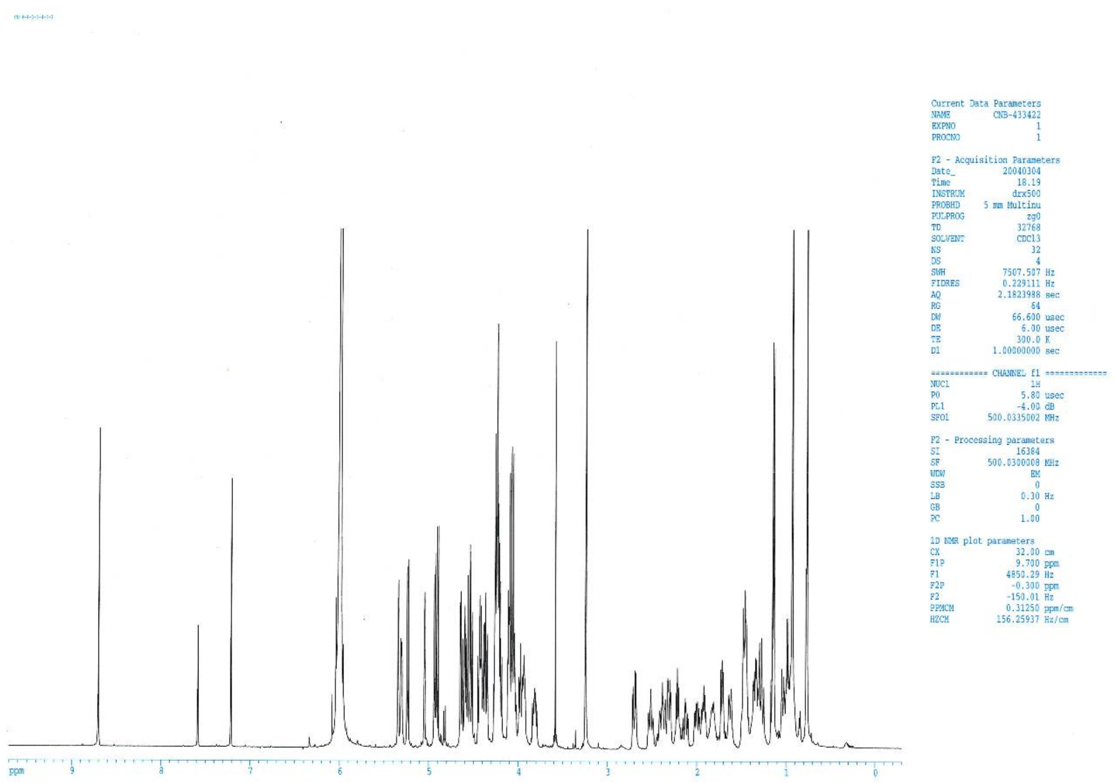

Figure S27. ^{1}H-NMR spectrum of **4**

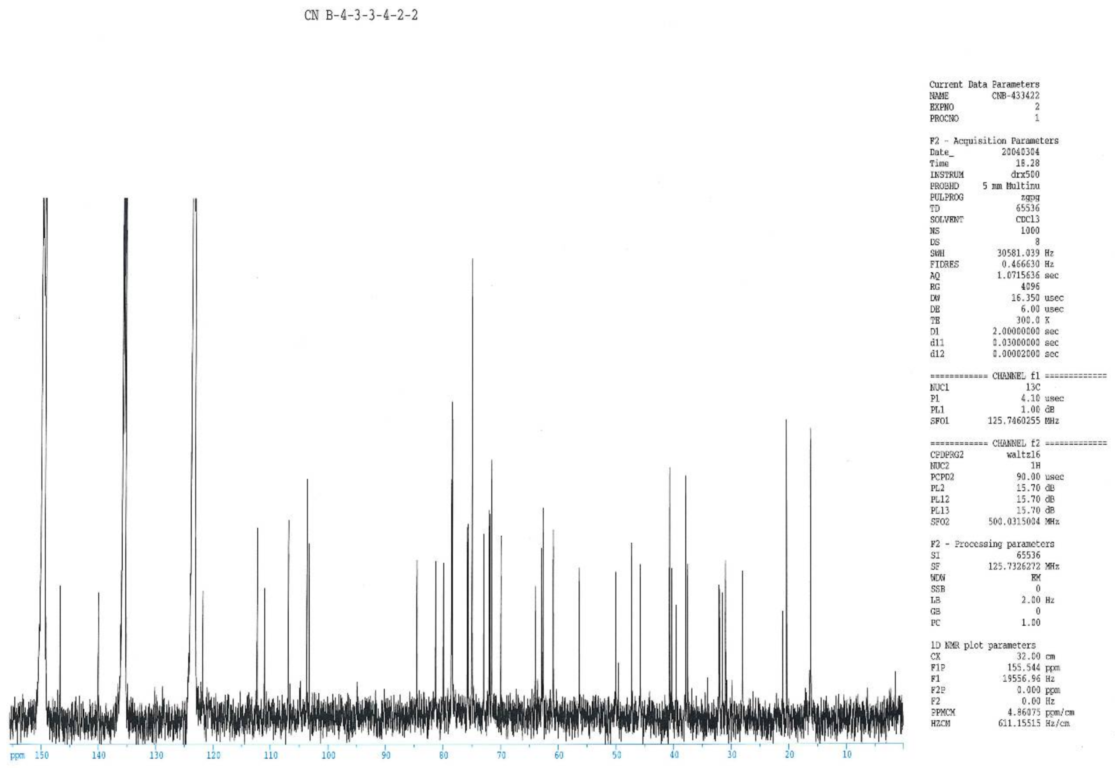

Figure S28. ^{13}C-NMR spectrum of **4**

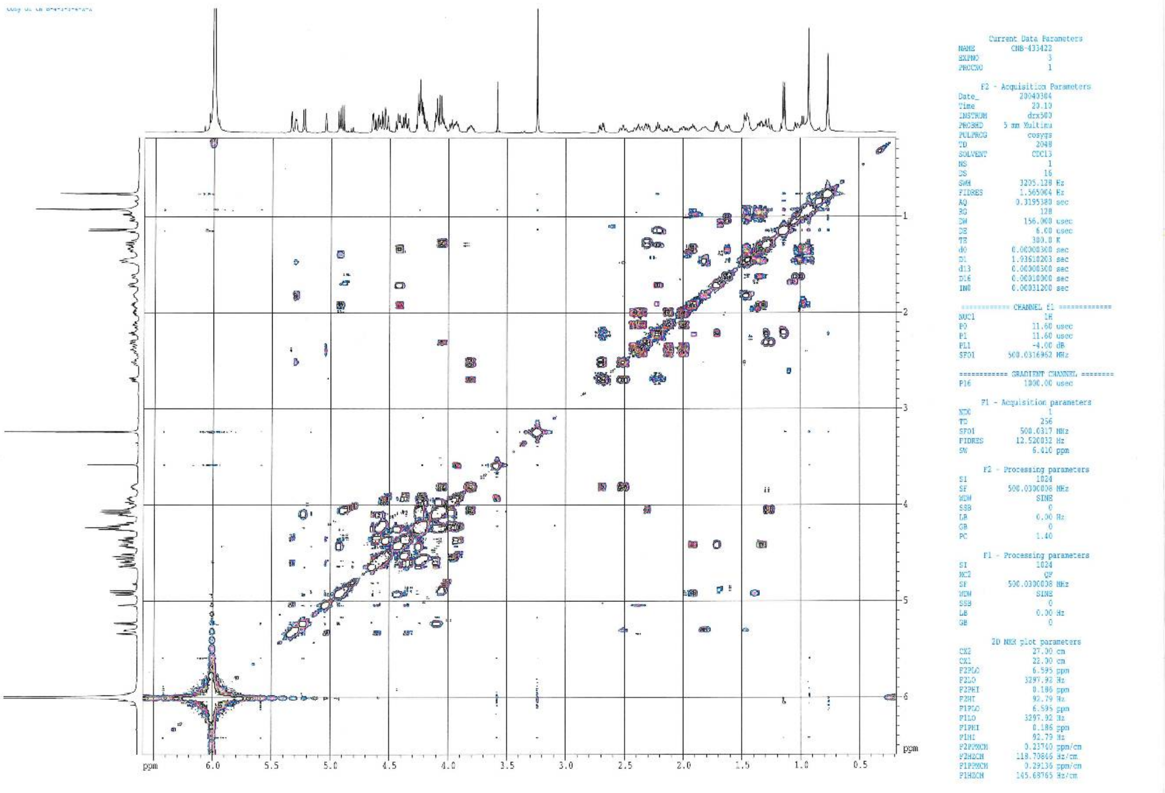

Figure S29. ^{1}H-^{1}H COSY spectrum of **4**

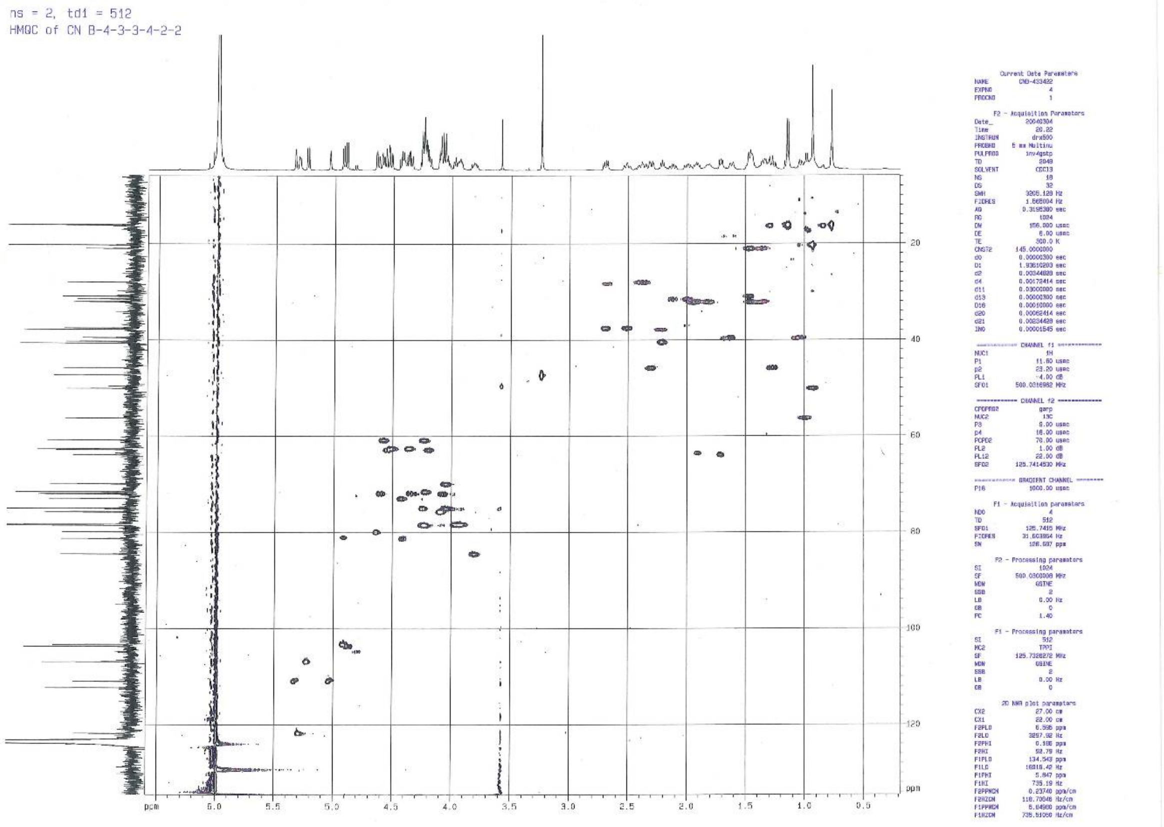

Figure S30. HMQC spectrum of **4**

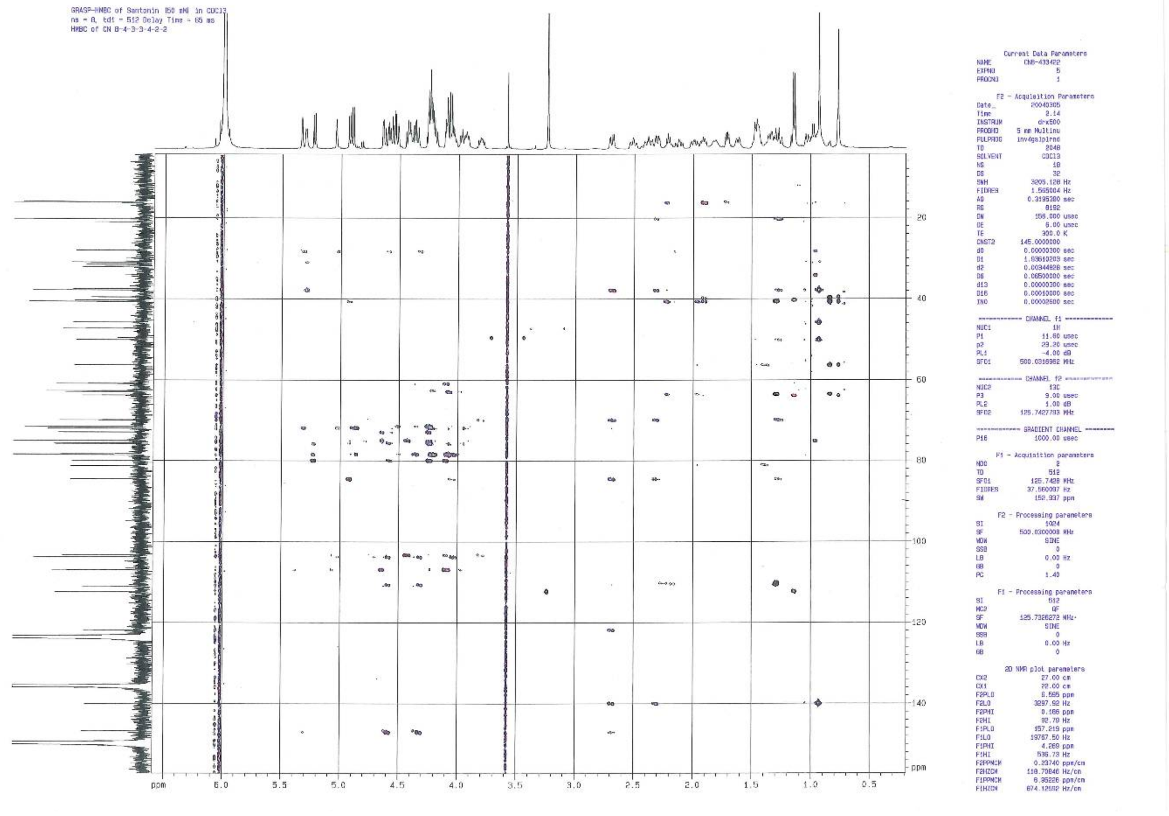

Figure S31. HMBC spectrum of **4**

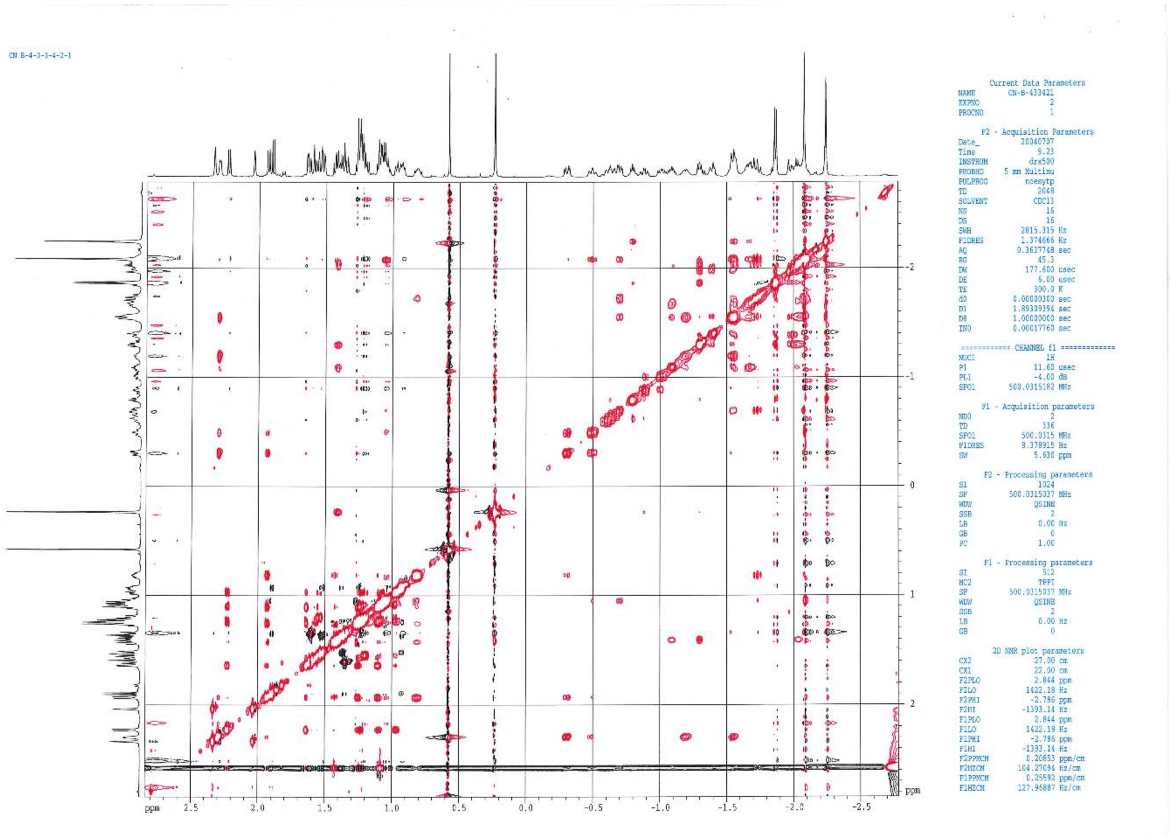

Figure S32. NOESY spectrum of **4**

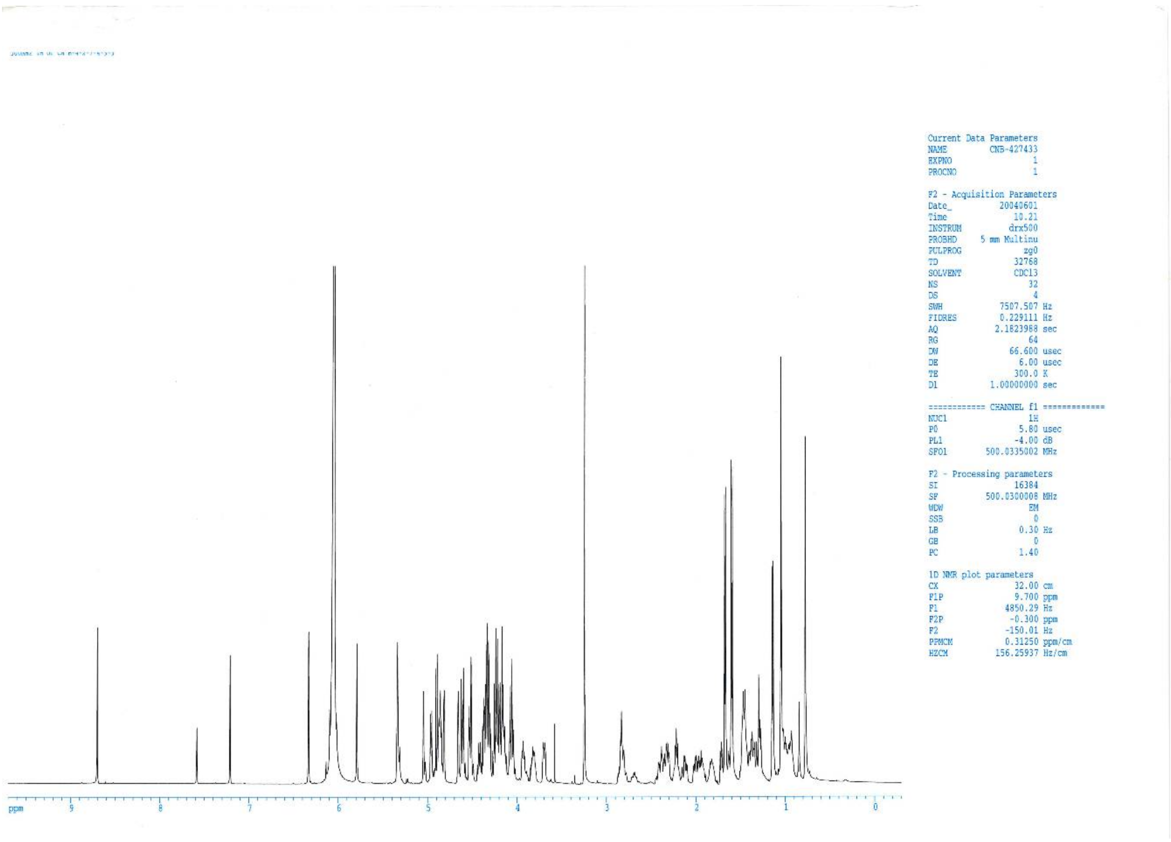

Figure S33. ^{1}H-NMR spectrum of **5**

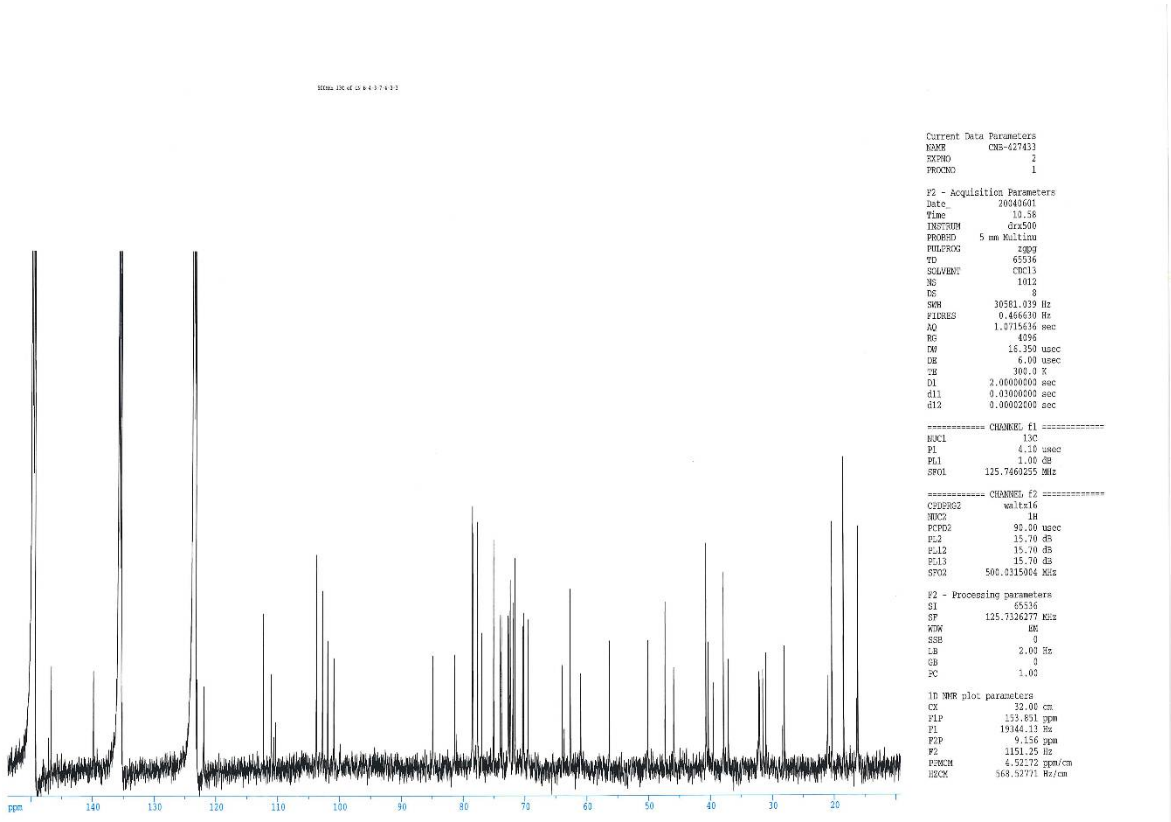

Figure S34. ^{13}C-NMR spectrum of **5**

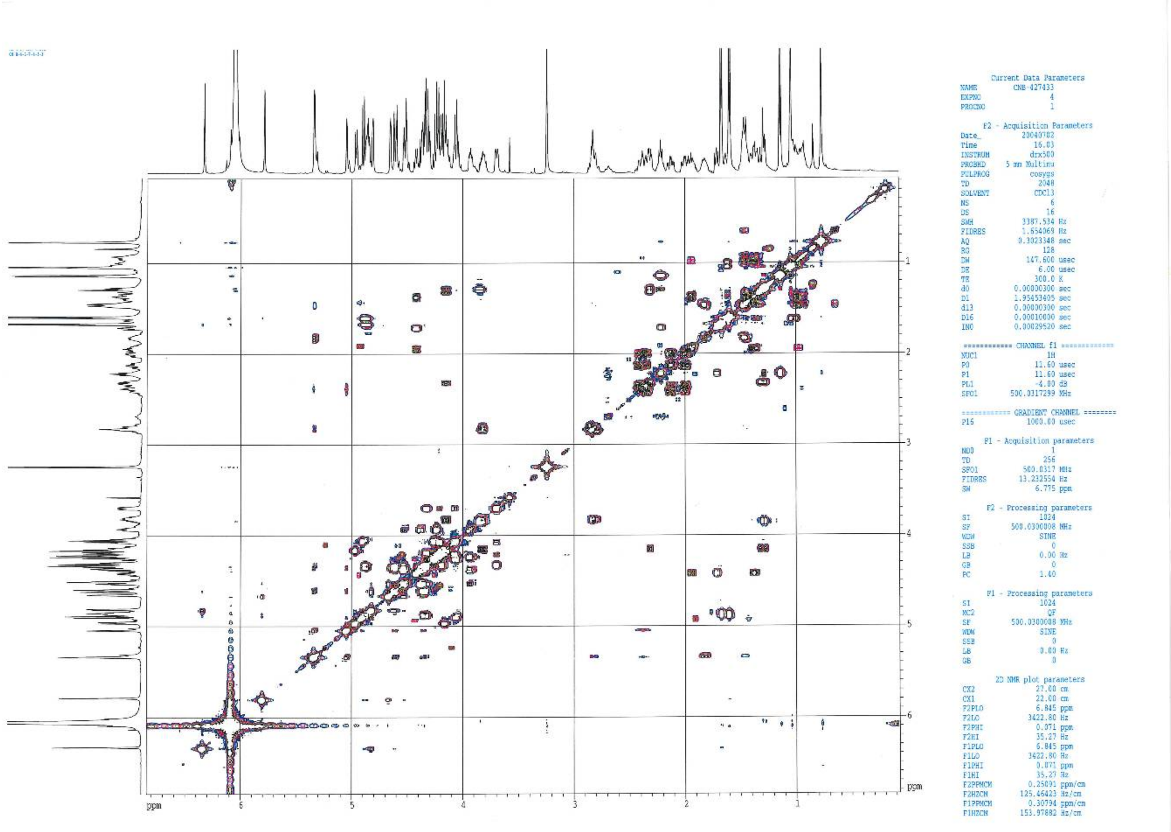

Figure S35. ^{1}H-^{1}H COSY spectrum of **5**

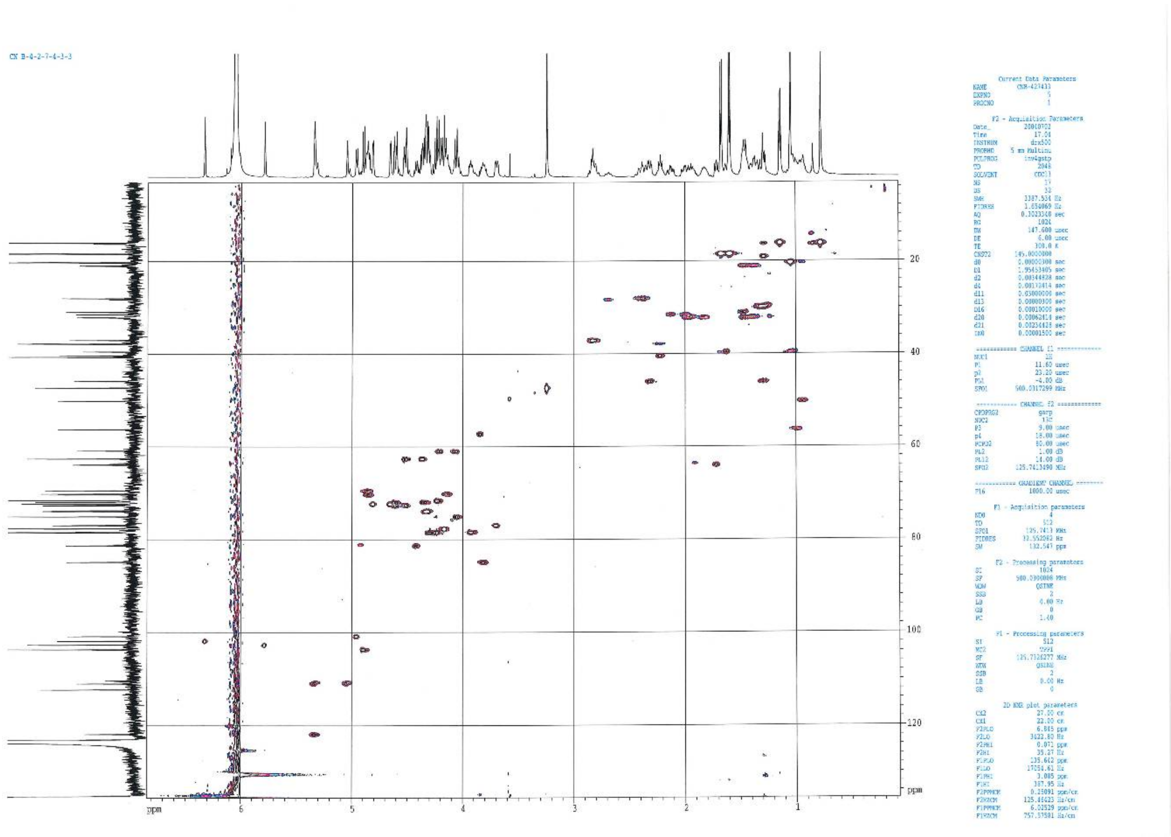

Figure S36. HMQC spectrum of **5**

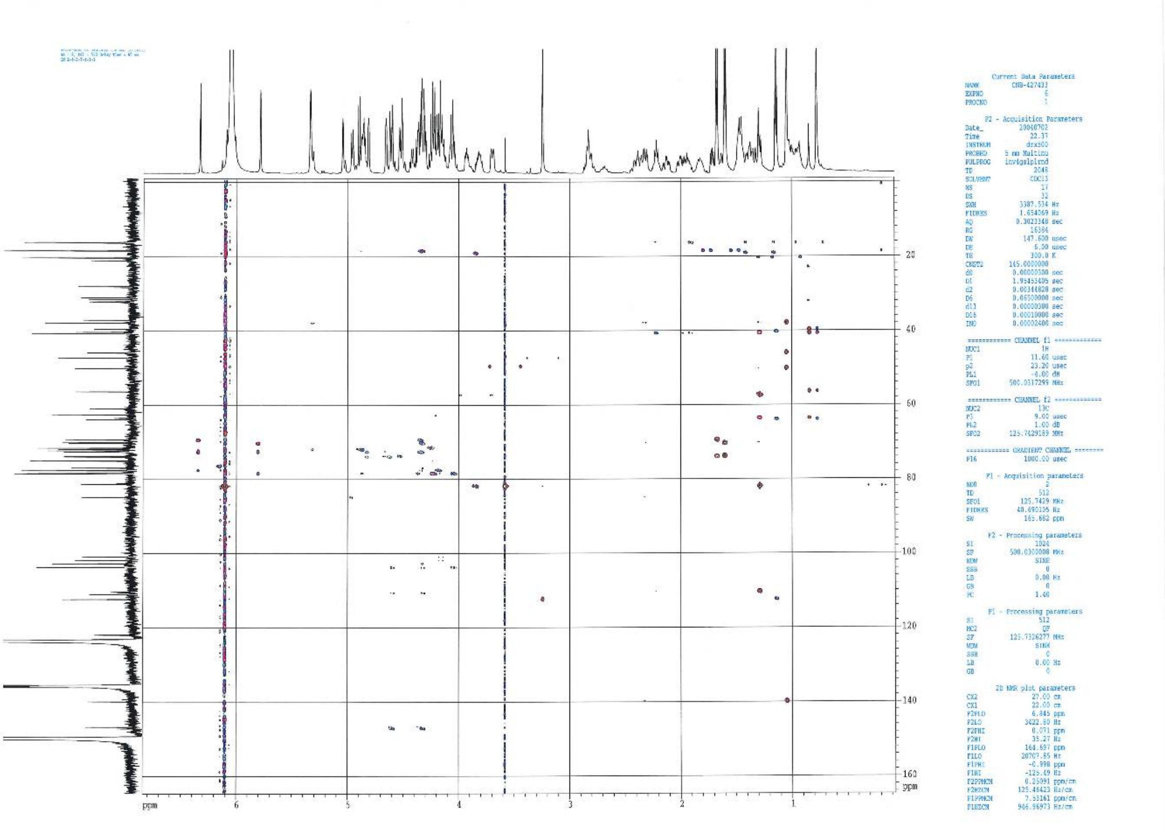

Figure S37. HMBC spectrum of **5**

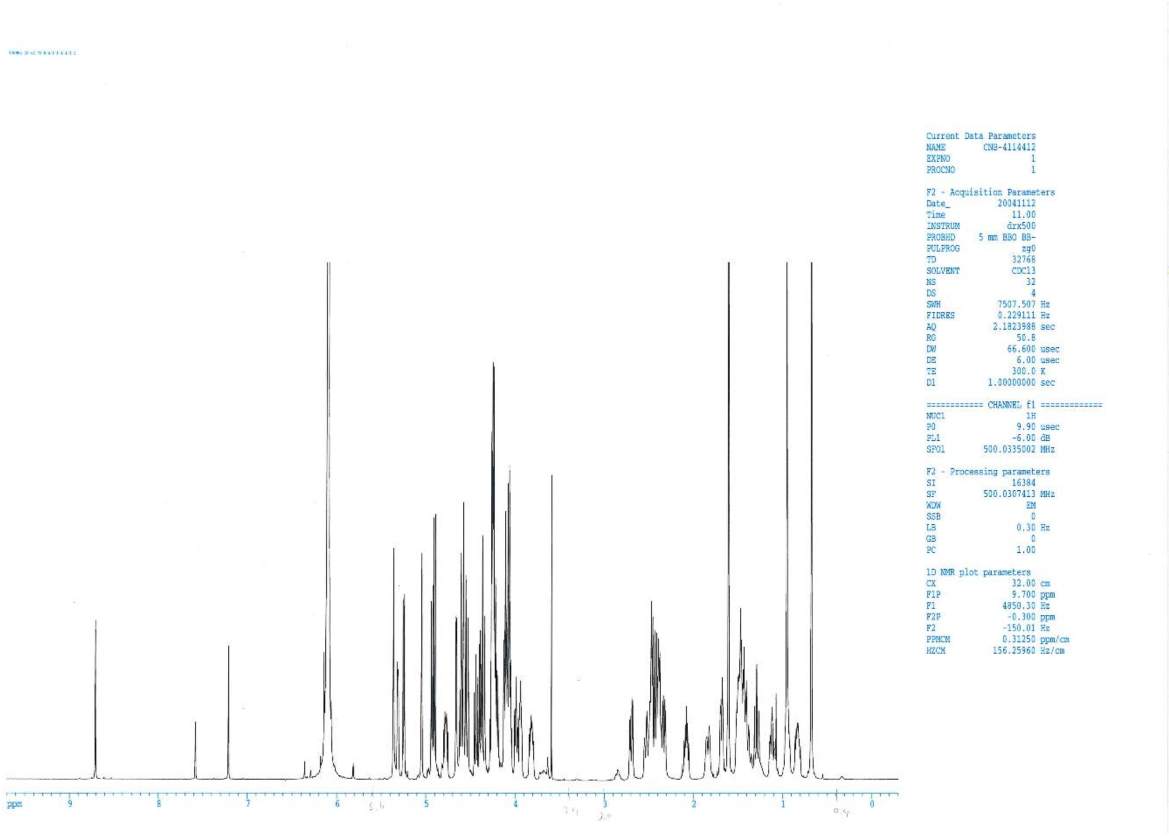

Figure S38. ^{1}H-NMR spectrum of **6**

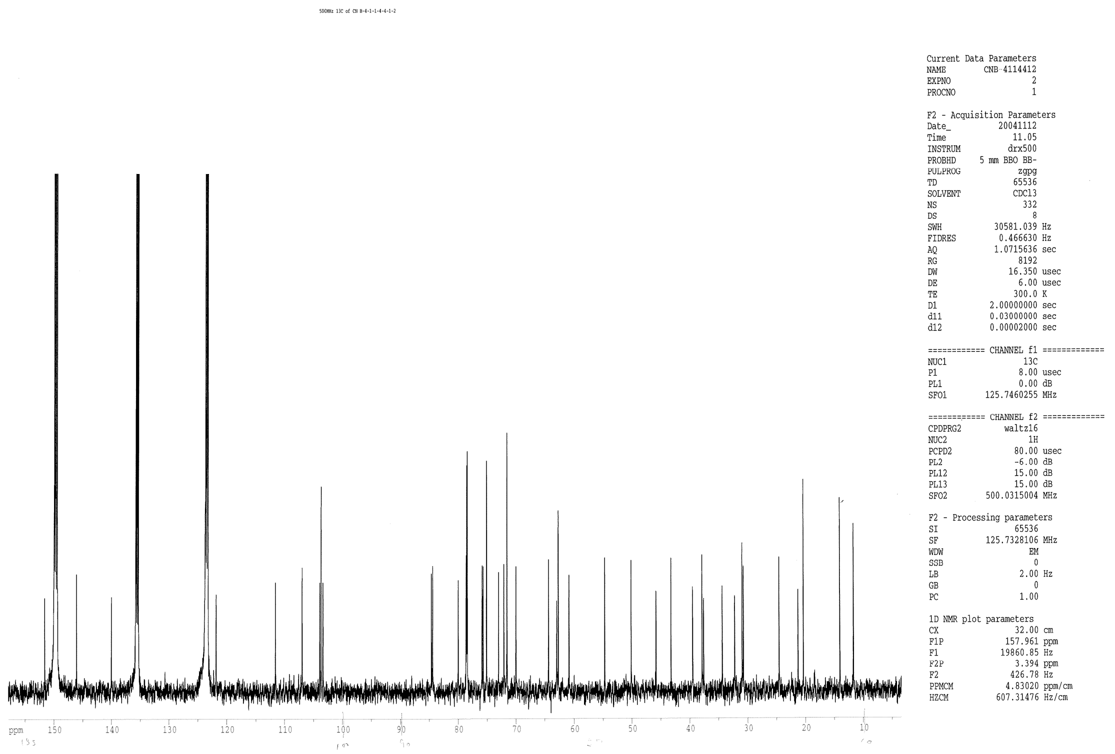

Figure S39. ^{13}C-NMR spectrum of **6**

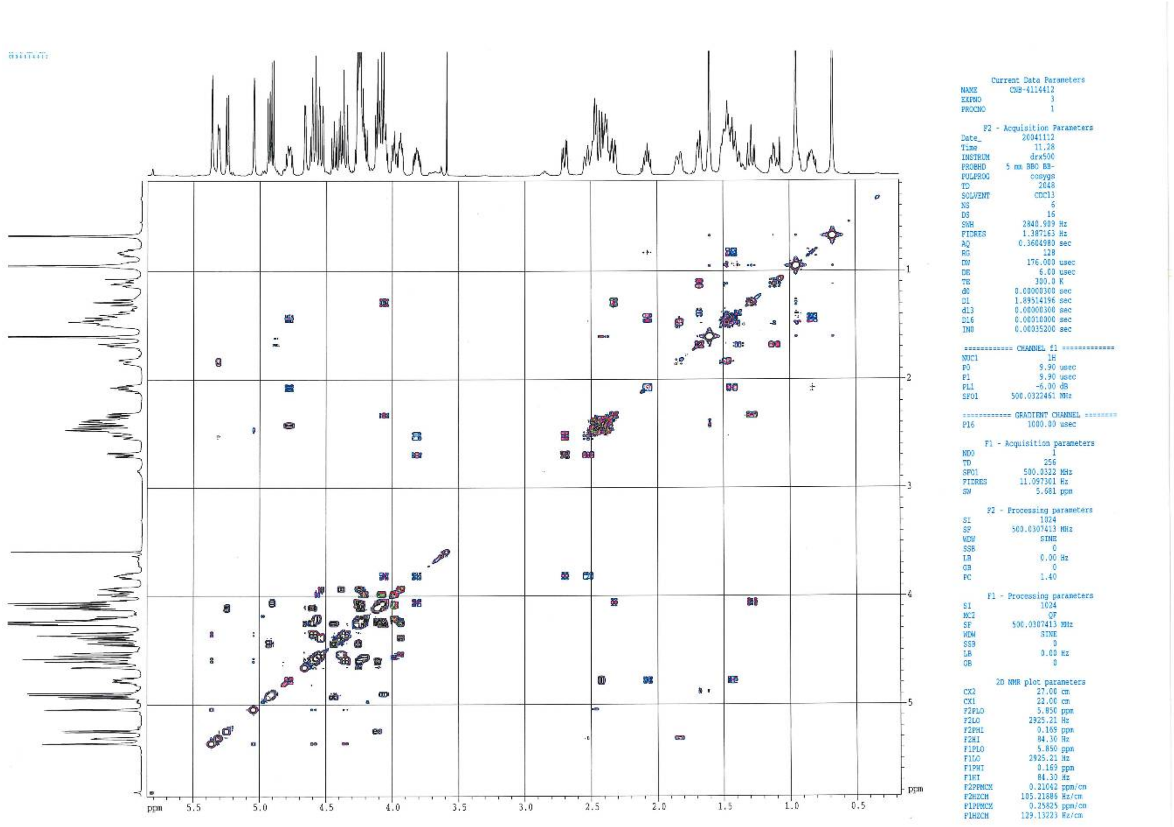

Figure S40. ^{1}H-^{1}H COSY spectrum of **6**

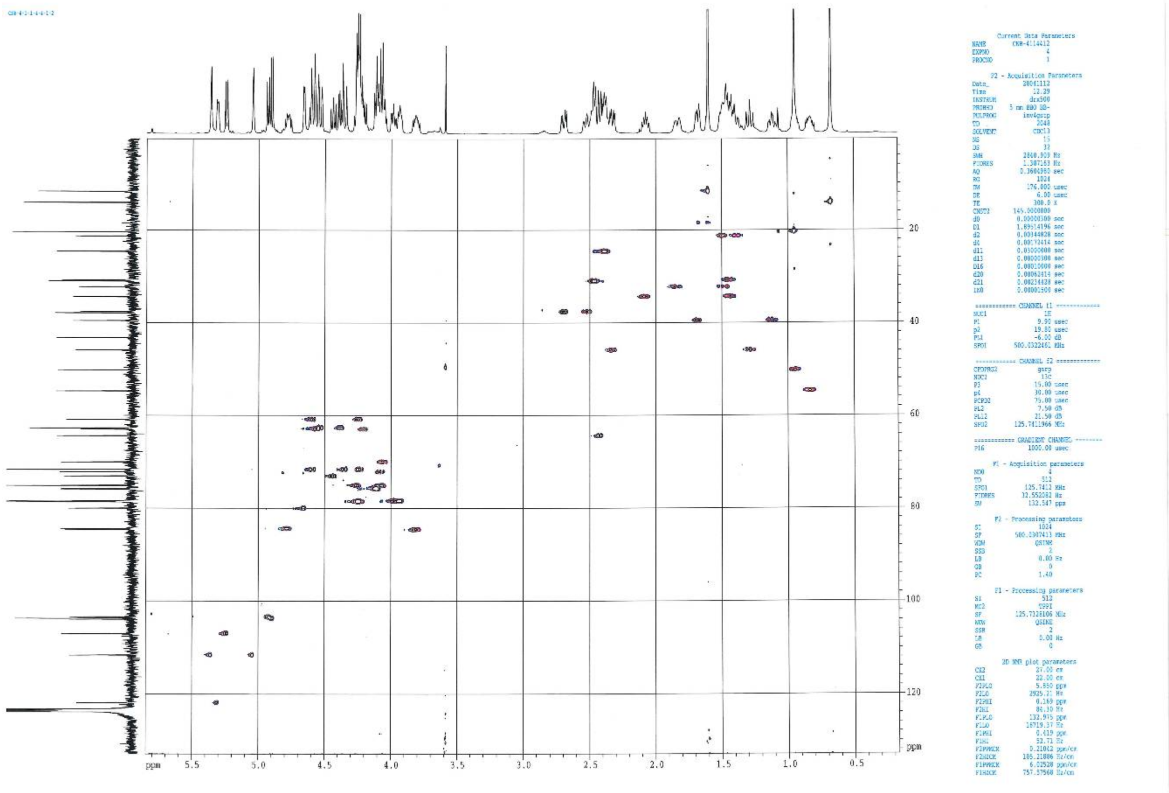

Figure S41. HMQC spectrum of **6**

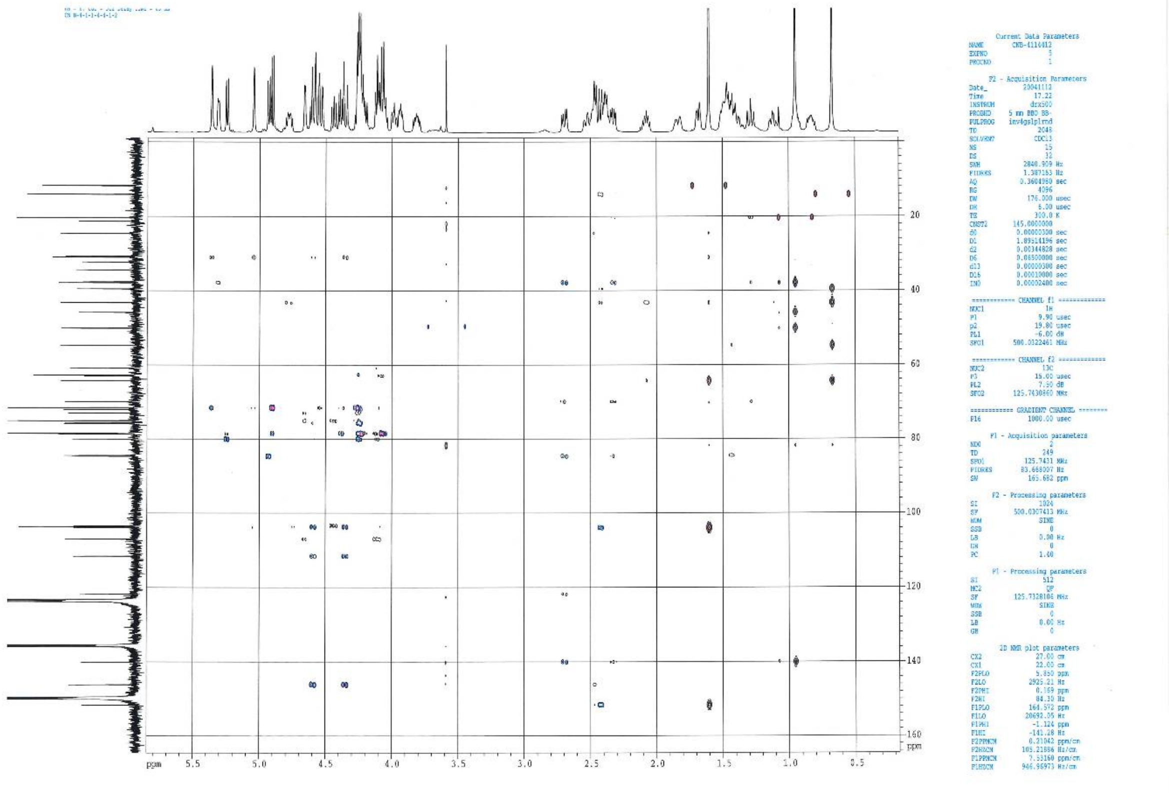

Figure S42. HMBC spectrum of **6**

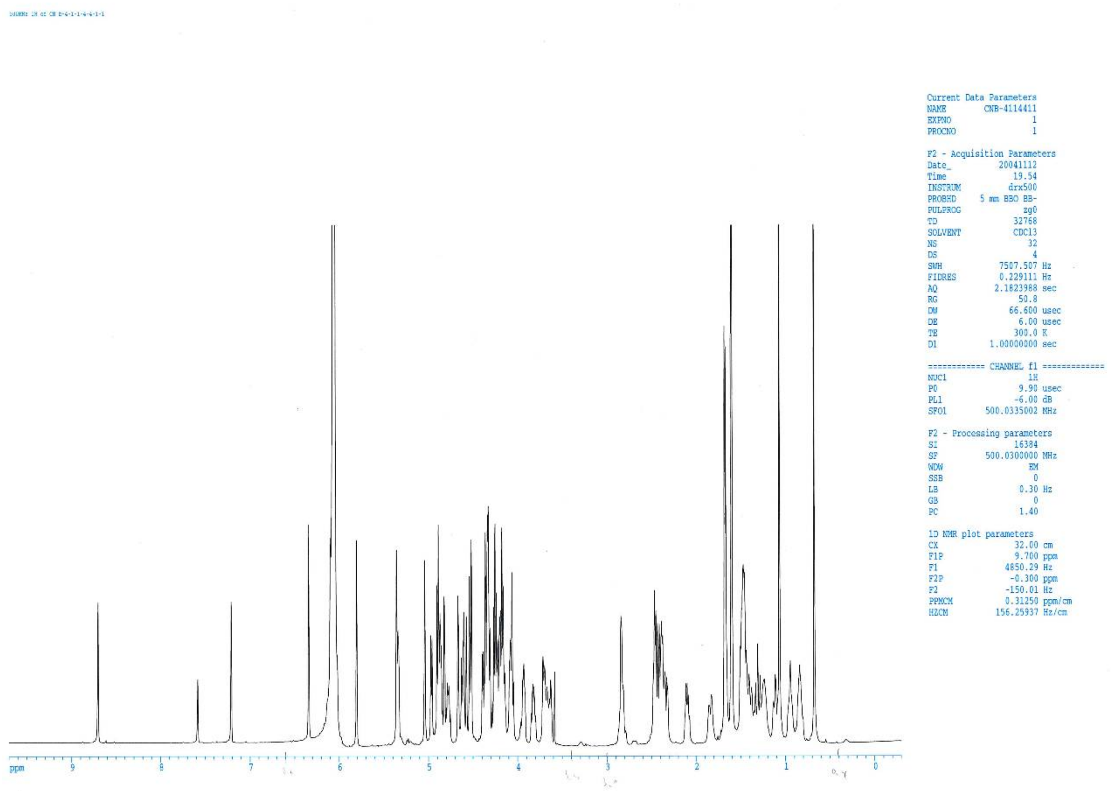

Figure S43. ^{1}H-NMR spectrum of **7**

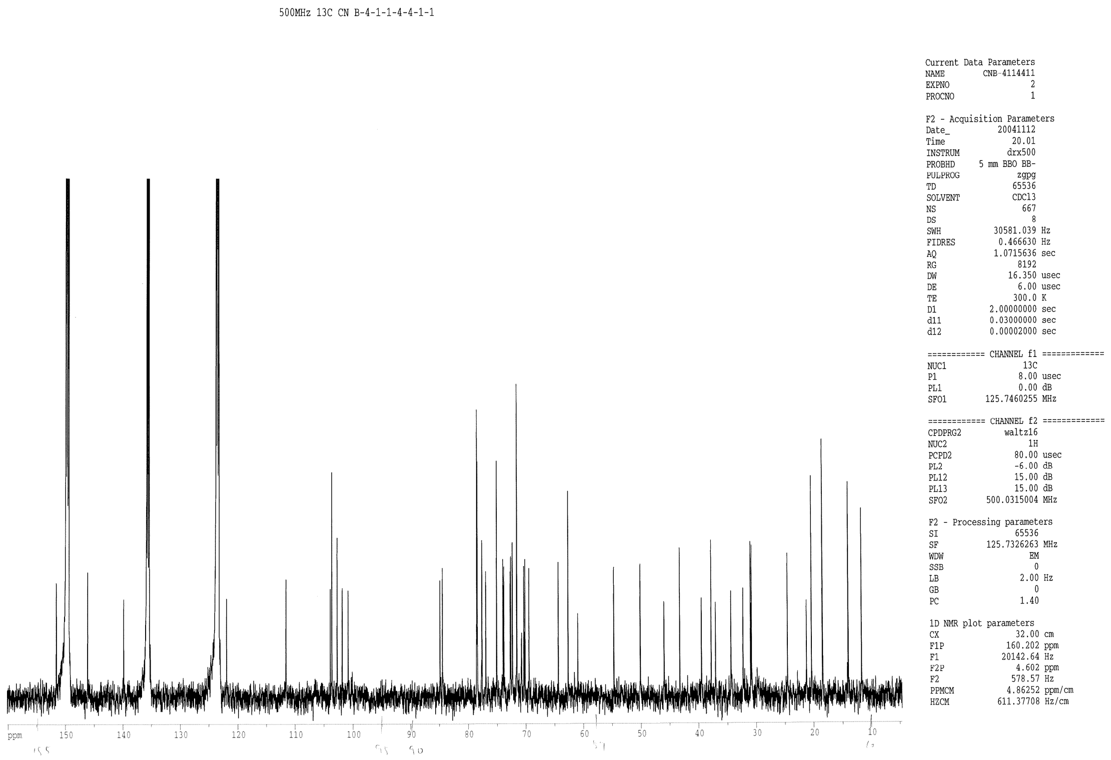

Figure S44. ^{13}C-NMR spectrum of **7**

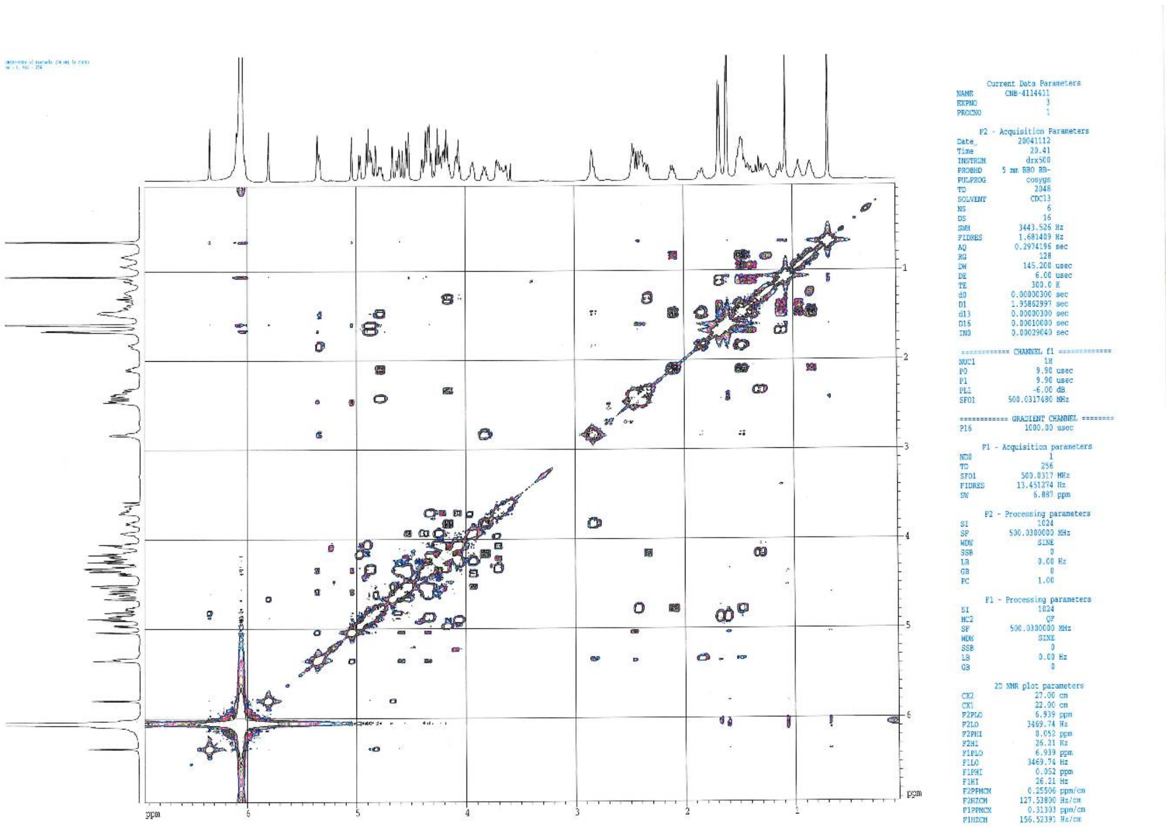

Figure S45. ^{1}H-^{1}H COSY spectrum of **7**

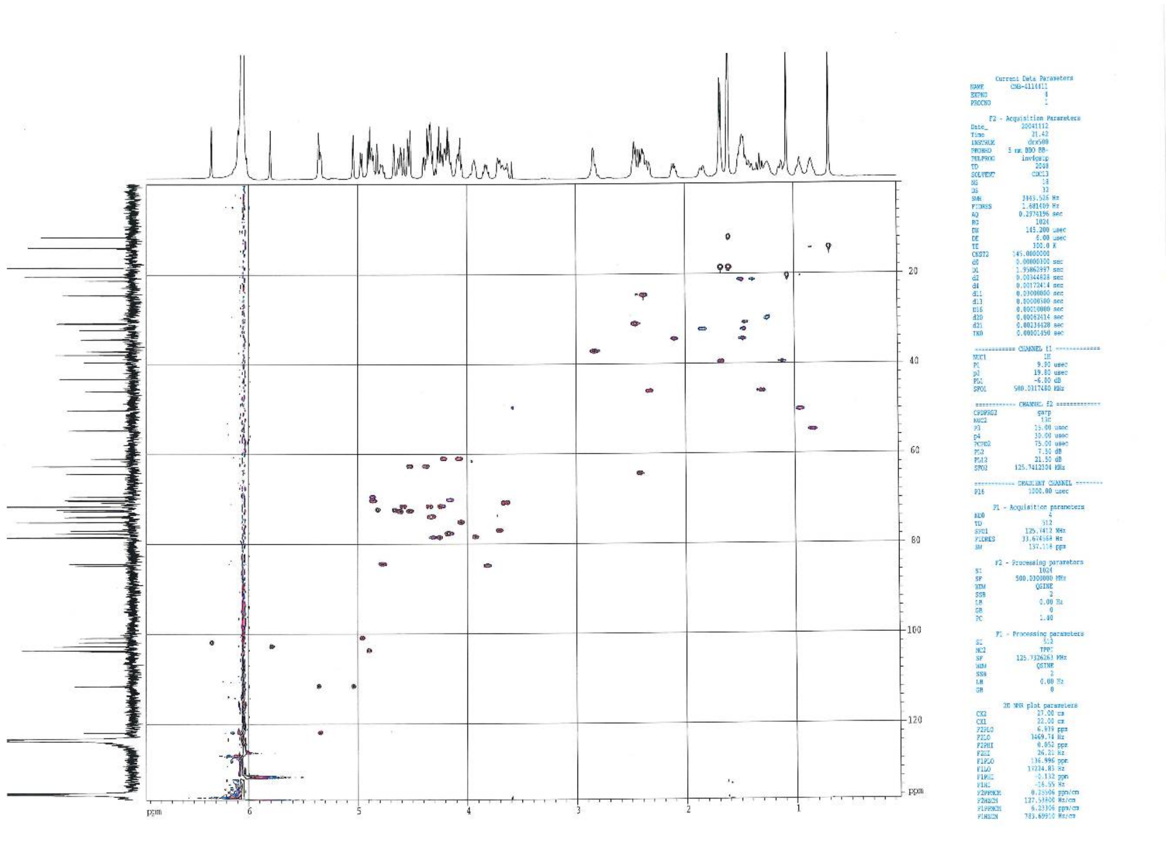

Figure S46. HMQC spectrum of **7**

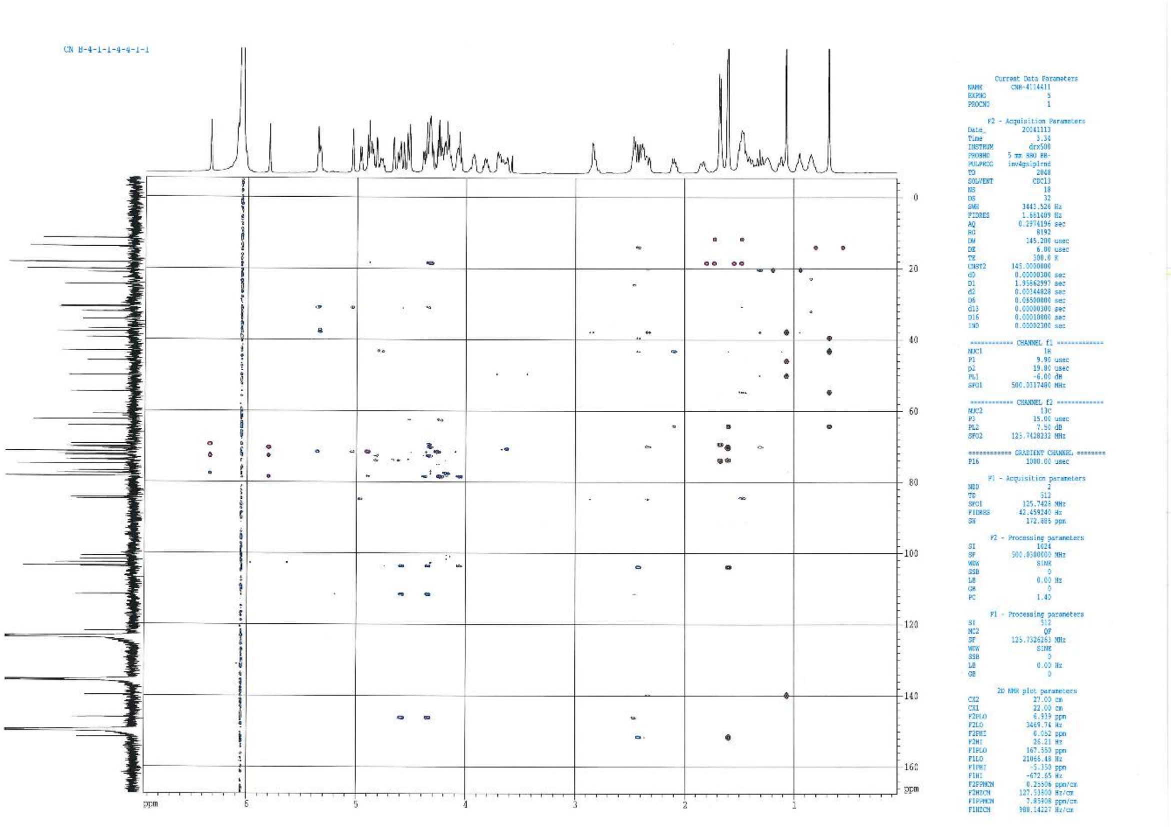

Figure S47. HMBC spectrum of **7**

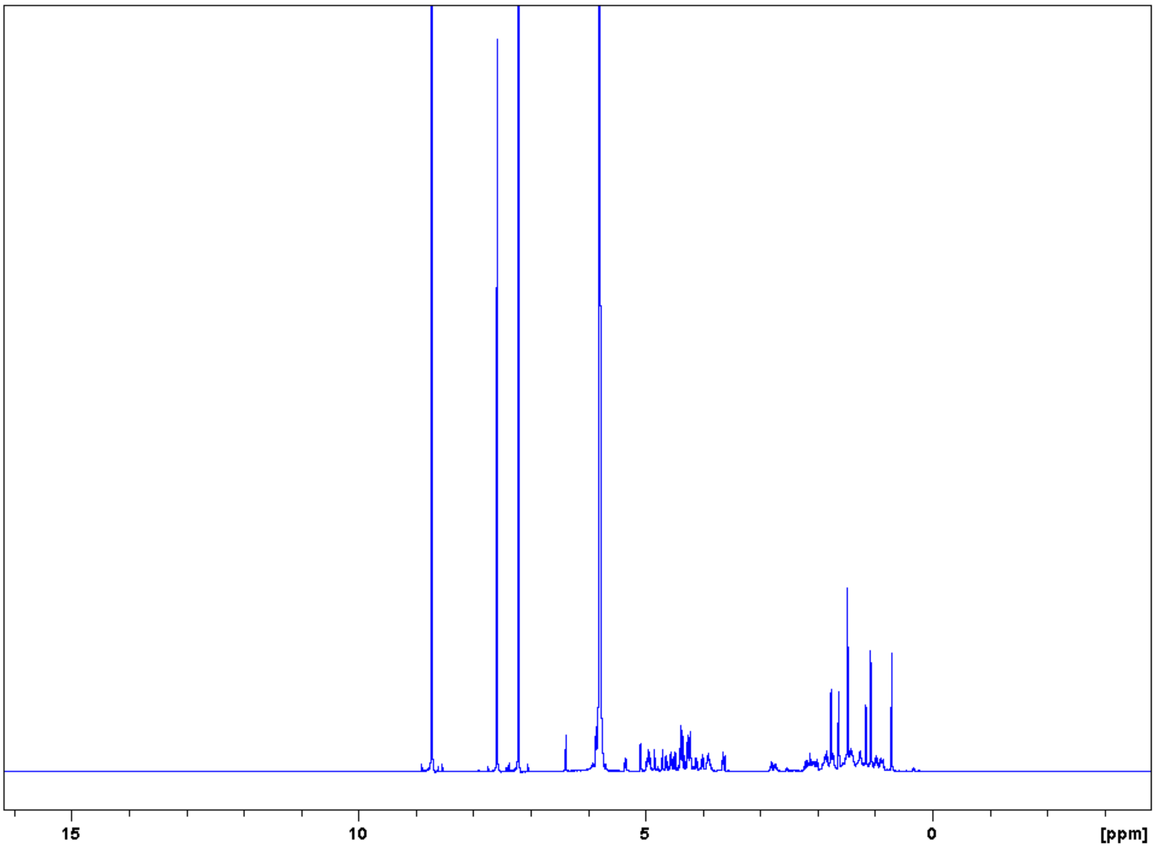

Figure S48. ^{1}H-NMR spectrum of **8**

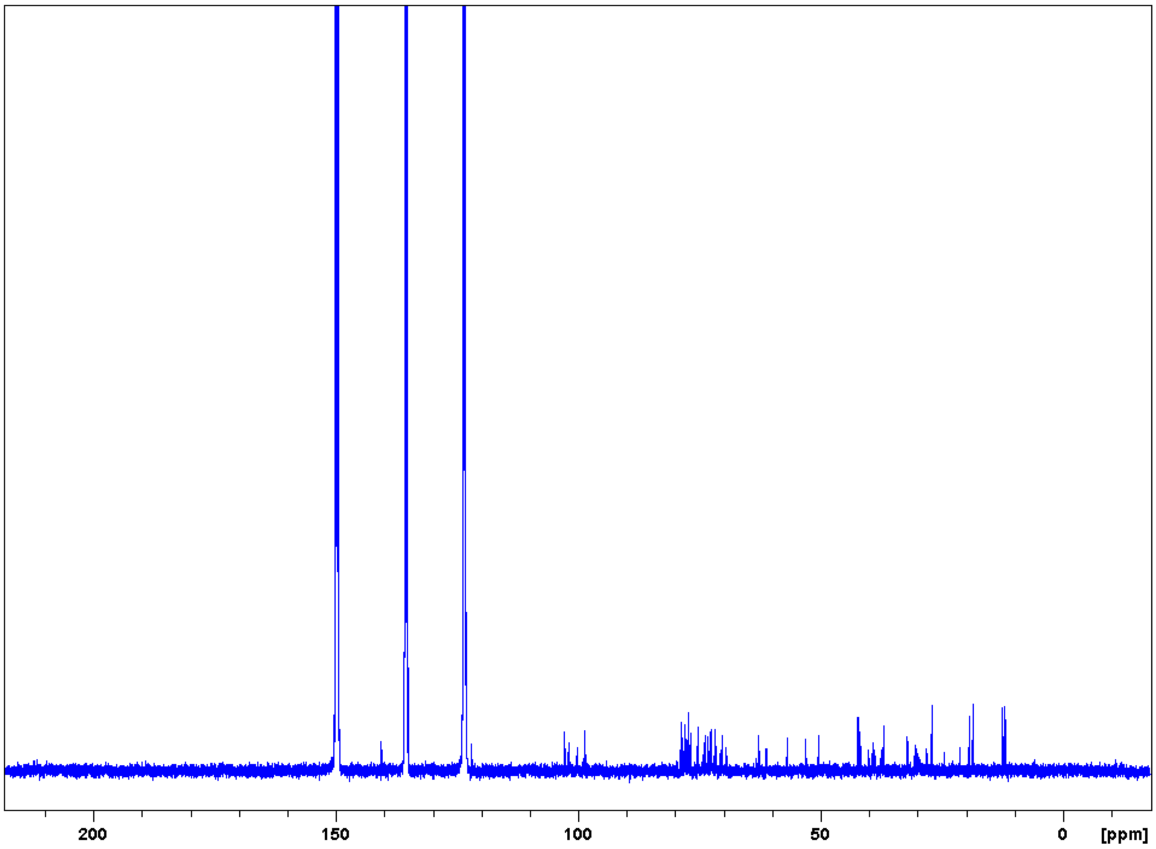

Figure S49. ^{13}C-NMR spectrum of **8**

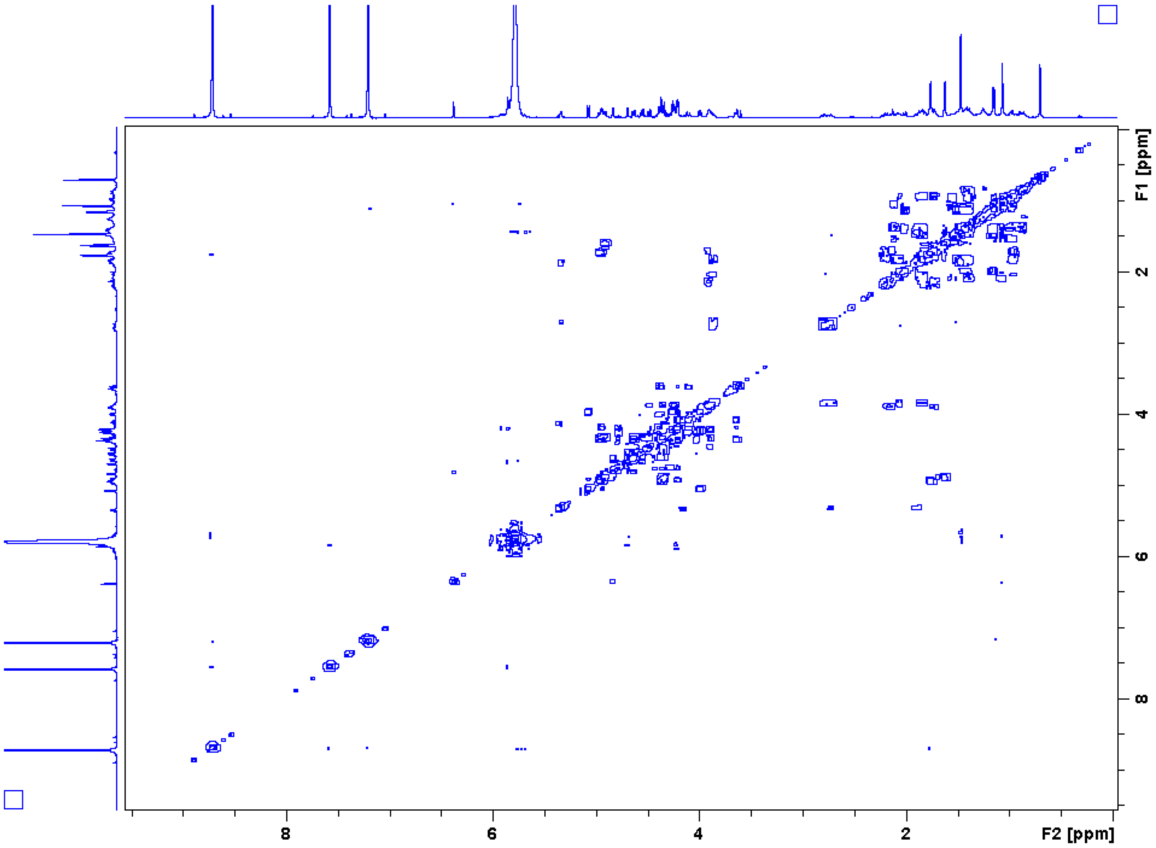

Figure S50. ^{1}H-^{1}H COSY spectrum of **8**

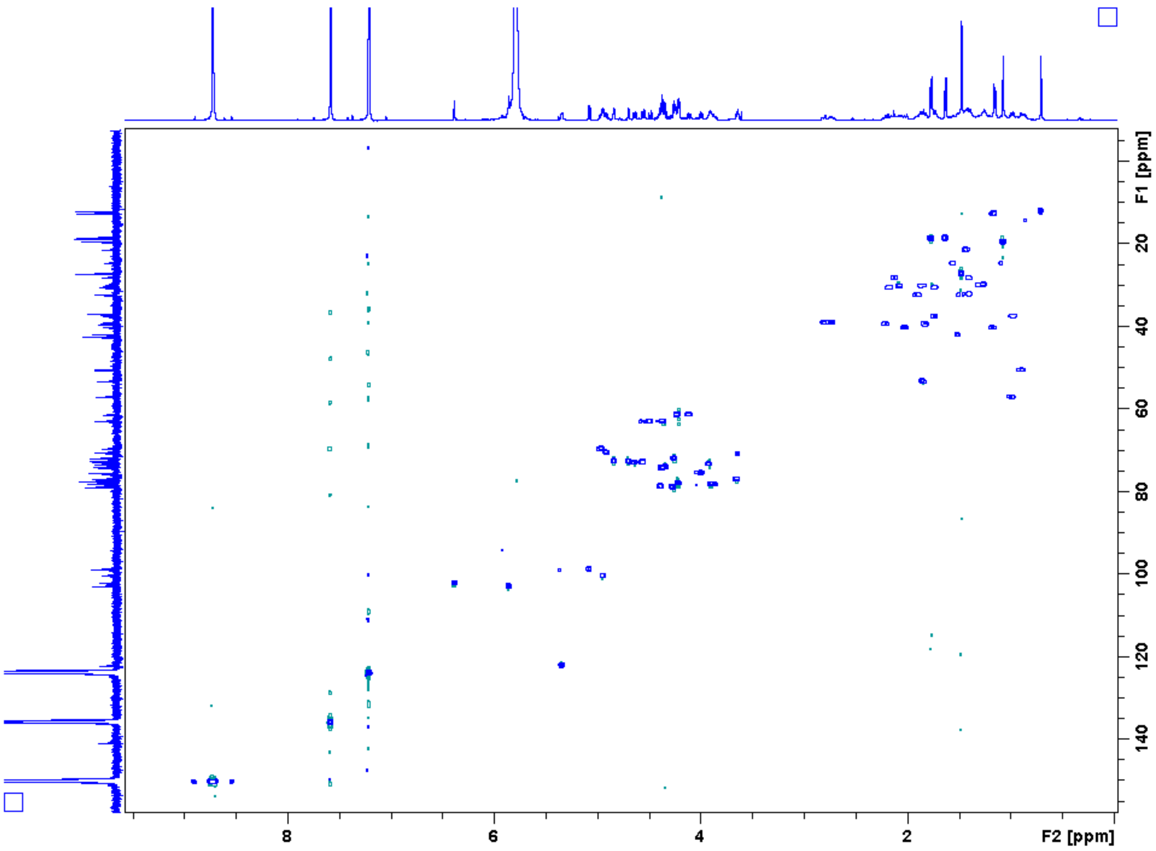

Figure S51. HMQC spectrum of **8**

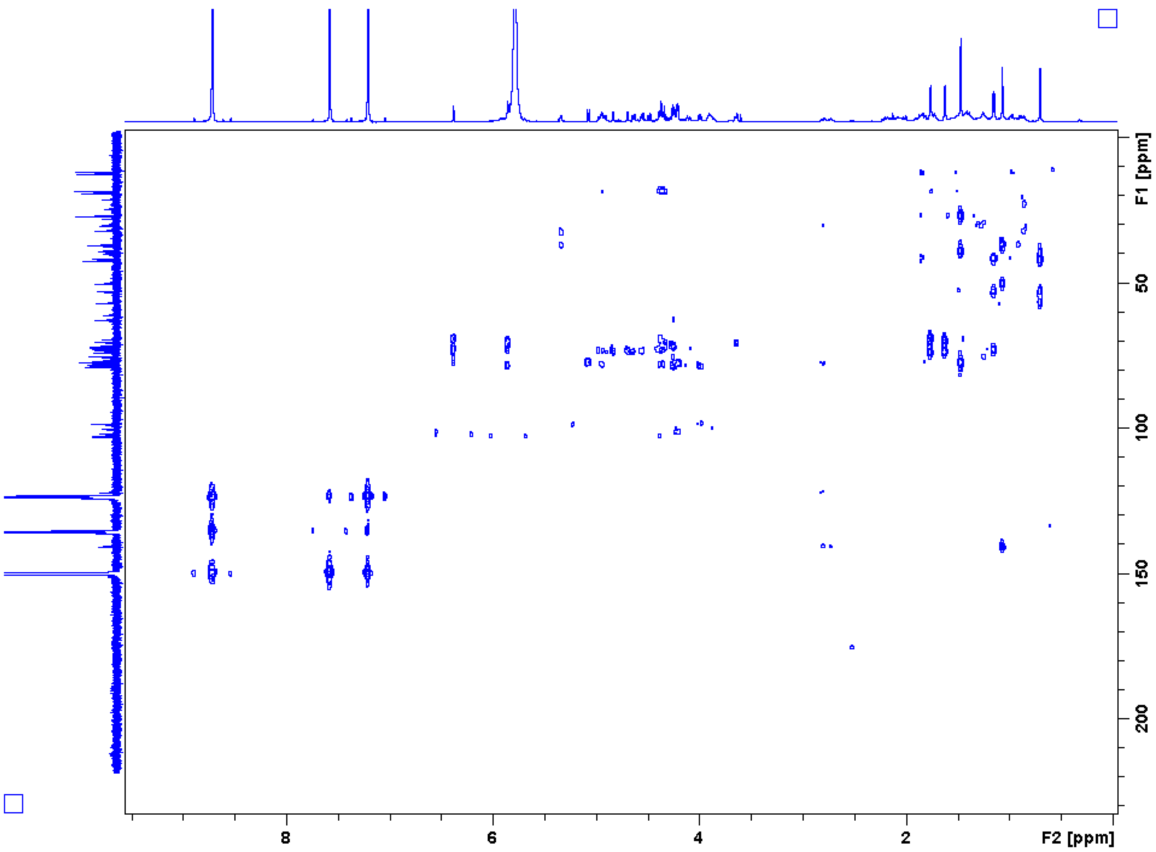

Figure S52. HMBC spectrum of **8**
